# Effect of visual imagery in COVID-19 social media posts on users’ perception

**DOI:** 10.7717/peerj-cs.1153

**Published:** 2022-11-15

**Authors:** Waleed M. Al-nuwaiser

**Affiliations:** Department of Computer Science, College of Computer and Information Sciences, Al-Imam Mohamed Ibn Saud Islamic University, Riyadh, Saudi Arabia

**Keywords:** Imagery posts, Social media, COVID-19, SAM, Valence, Arousal, Dominance, Risk perception, Credibility and engagement

## Abstract

People receive a wide variety of news from social media. They especially look for information on social media in times of crisis with the desire to assess the risk they face. This risk assessment, and other aspects of user reactions, may be affected by characteristics of the social media post relaying certain information. Thus, it is critical to understand these characteristics to deliver information with the reactions in mind. This study investigated various types of imagery used as thumbnails in social media posts regarding news about the COVID-19 pandemic. In an experimental design, 300 participants viewed social media posts containing each of the three types of imagery: data visualization (directly about risk information), advisory (not containing direct risk information, but instead help on how to lower risk), or clickbait (containing no risk-related information, just generic visuals). After observing the social media posts, they answered questionnaires measuring their emotions (valence, arousal, and dominance), risk perception, perceived credibility of the post, and engagement. The participants also indicated their emotions towards and interest in COVID-19 news coverage, age, gender, and how often and actively they use social media. These variables acted as controls. The data were analysed using mixed linear models. Results indicated that advisory imagery positively influenced valence, arousal, dominance, credibility, and (lower) risk perception. Alternatively, imagery showing data visualizations yielded low valence, arousal, dominance, credibility, and high risk perception. Clickbait-styled thumbnails which carry no information are usually measured between the other two types. The type of imagery did not affect the motivation to engage with a post. Aside from visual imagery, most variables were affected by COVID sentiment and the usual activity on social media. These study results indicate that one should use advisory imagery for more comfortable news delivery and data visualization when the poster wishes to warn users of existing risks.

## Introduction

Social media have become prominent sources of information for many people (Westerman, Spence & Van Der Heide, 2014). Therefore, it was a matter of time before social media started being used as a tool for swaying public opinion through fake news (Deutsch, 2017; English, 2017; Ghanem, Rosso & Rangel, 2020; Grech & Masukume, 2017; Guess, Nagler & Tucker, 2019) and even trying to win over elections (Allcott & Gentzkow, 2017; Barthel, Mitchell & Holcomb, 2016; Shane, 2017). While these are bad influences, social media may exert good influences as well—it provides rapid communication in times of crisis, such as during the cholera outbreak in Haiti (Sutter, 2010). Therefore, it is crucial to investigate how social media posts may be framed and shown to users to show credible information and have them see it as credible.

## Background

### The three-component emotion model

Ever since the pioneering work of Wundt (1913), there has been a line of thought in psychology that emotions can be assessed through three dimensions. Wundt termed them *lust* (pleasure), *spannung* (tension), and *beruhigung* (inhibition). In later years, other scholars determined that three dimensions precisely describe emotions, and although they used slightly different names, they referred to very similar phenomena. For instance, a group of researchers (Osgood, Suci & Tannenbaum, 1957) utilized factor analysis on participants’ estimations of words on 50 bipolar scales (for example, hot or cold). They determined that three factors can successfully explain them—evaluation, activity, and potency. They have also shown that these aspects of emotion are usable in different cultures and for non-verbal stimuli (Osgood, Suci & Tannenbaum, 1957). Further research found that the three dimensions assess body parts, facial gestures (Mehrabian, 1970), and human interactions (Mehrabian & Russell, 1974). Henceforth, the semantic differential scale developed by Mehrabian & Russell (1974) was widely used to measure the three dimensions of emotion. It contains 18 pairs of opposite adjectives (for instance, happy—unhappy), and between the adjectives, there is a nine-point scale (ranging from −4 to 4), on which the participant can indicate how close they are to either of the poles. Then, they calculate a composite score to measure the degree to which each of the three dimensions of emotion is present in a participant. This methodology was and is still widely used, but there was also room for improvement. If many stimuli need to be measured, the test can be very time-consuming, and it is language-dependent.

Those shortcomings are why Prof. Peter Lang and his colleagues developed the self-assessment mannequin (SAM) method (Bradley & Lang, 1994; Lang, 1980). This method, which will be described in more detail later, only utilizes three scales and measures the same three dimensions of emotion, albeit named slightly differently—valence, arousal, and dominance. The valence dimension relates to how positive or negative an emotion is. The arousal dimension relates to how excited or calm the person is. Finally, the dominance dimension relates to how much in control the person feels, compared to feeling like they do not influence the situation. As will be shown more extensively, researchers have successfully used this methodology to measure these dimensions of emotion in a fast and exact manner in various contexts (Isomursu et al., 2007).

### Social effects of the COVID-19 pandemic

The emergence of the COVID-19 pandemic has had incredibly destructive effects on people worldwide, both in terms of physical illness and death and regarding psychological and social effects. As any other crisis does, the pandemic changed how people lived. They stayed at home much more, made less contact with their friends and loved ones, and changed how they worked or studied (through online media). This behaviour brought about many issues with mental health (AlAzzam et al., 2021; Almandoz et al., 2021; Biro-Hannah, 2021; Cao et al., 2020; Liang et al., 2020; Pietrabissa et al., 2021; Vahedian-Azimi et al., 2020) as societal changes influenced people.

To alleviate these changes, people commonly turned to social media networks to connect with others and find information, which is typical for crises. For example, the Zika virus outbreak has caused people to use social media for information and assurance. More specifically, a study (Gui et al., 2017) demonstrated that social media was the primary channel people used to assess the risk associated with the Zika virus outbreak in 2016. Similarly, the ongoing COVID-19 pandemic inspired a lot of news coverage, fear, and risk in everyday lives. Analyses of social media post sentiments showed that, during the beginning of the pandemic, people were experiencing multiple pandemic-related threats, including illness, secondary health conditions, economic, socio-behavioural, and institutional risks. They also used social media to find information which would help them with their day-to-day decision making (Pine et al., 2021). Therefore, it is crucial to understand how different ways of shaping information on social media may influence users’ perceptions of and reactions to such information.

## Related work

In recent years, research has explored various aspects of social media experiences. A critical aspect of reading information on social media is knowing who posted it and deliberating on whether that source is trustworthy. To understand the factors which contribute to (not) trusting a source, Kim & Dennis (2019) conducted an experimental study. They manipulated the alleged source of an article and tested whether it nudged the readers towards taking a closer look at the source. The result of the nudging was that the users were more sceptical when attention was brought to the source but were more trusting towards the article (rated it with higher believability) when the source had a high rating. Increased believability encouraged higher engagement with the article. Researchers measured this engagement through interaction with the post—liking, sharing, or commenting upon it. In the remainder of this article, engagement means interacting with a post. They also had more trust in articles that aligned with their views, thus manifesting confirmation bias (Kim, Moravec & Dennis, 2019; Kim & Dennis, 2019).

As mentioned earlier, social media is a vital source of information during crises (Gui et al., 2017; Pine et al., 2021). One of the main types of information people seek during an emergency is risk information—How much in danger are we, and should we be scared? Throughout the COVID-19 pandemic, especially at the beginning, people were frightened and saw the risk of dangerous health outcomes happening to them and everyone else as very high (Dryhurst et al., 2020). People both expressed anxiety and sought answers and knowledge online, which would help them deal with the new situation, as shown in a study on the Korean social media space at the beginning of the pandemic (Jo et al., 2020). As already mentioned, a large *corpus* of research (*e.g*., Bica et al., 2019; Gui et al., 2017; Pine et al., 2021) shows that risk assessment occurs primarily through social media, which means that health officials should strive to utilize social media to communicate risk precisely. They should do it knowing how every element of a social media post affects the potential reader.

However, only a handful of studies have investigated the effects that various aspects of social media posts have on users. One line of research by Kim, Moravec & Dennis (2019) focused on fake news and how reliability ratings can push users towards being more sceptical. However, none of the mainstream social media platforms (Facebook, Instagram, Twitter, YouTube, and TikTok) have credibility ratings. Another line of research is investigating how headlines used in social media posts affect user perceptions. In that vein, Janét, Richards & Landrum (2022) investigated three types of headlines about environmental topics: traditional ones (with straightforward information), those that ask questions, and forward-referencing headlines (‘You’ll never believe what…’). They found that headlines did not affect story engagement or selection but did affect credibility, and the question-formatted headlines seemed the least credible. The participants’ political views and scientific curiosity also affected the engagement and credibility of the stories (Janét, Richards & Landrum, 2022). Another study found that users typically share news on social media through more interpersonal and subjective methods. Recent studies have called for more investigation into how they share news and which determinants lead to which outcomes in their perception (Welbers & Opgenhaffen, 2019).

Another study aimed to answer this question investigated how the emotional tone of a news social media post affects users’ engagement. Choi, Lee & Ji (2021) examined ~15,000 social media posts using software to recognize emotions in the thumbnails and the text. They determined that social media posts with negative text were more commented on and shared, while positive texts earned more reactions. Furthermore, sadness was the thumbnail emotion that yielded the most engagement. Other researchers have shown that visual aspects of a social media post can also be important in relaying risk-related information regarding natural disasters (Bica et al., 2019).

Aside from the text in the post, the visual aspects of social media posts influence users as well. Also, social media is a vital space to disseminate information in times of crisis in general and during the COVID-19 pandemic specifically. Thus, this study aimed to investigate the effect of various types of imagery used in COVID-related social media posts. The study’s primary research question is to determine the effects that different types of visual stimuli used in thumbnails have on how social media users perceive social media posts regarding information about COVID-19. The three types of visual stimuli are (a) those containing charts with information about the pandemic to convey risk information; (b) those with advice, which do not provide any information about the risk of the pandemic but instead just advise on how to reduce personal and community risks; and (c) those with no information and just striking visuals that help the user identify the topic of the article.

There are six specific research questions:

RQ1: What is the effect of the three types of visual stimuli on the valence of emotions that users feel after looking at a COVID-related social media post?

RQ2: What is the effect of the three types of visual stimuli on the arousal that users feel after looking at a COVID-related social media post?

RQ3: What is the effect of the three types of visual stimuli on the dominance that users feel after looking at a COVID-related social media post?

RQ4: What is the effect of the three types of visual stimuli on the users’ perception of risk after looking at a COVID-related social media post?

RQ5: What is the effect of the three types of visual stimuli on the users’ engagement with a COVID-related social media post?

RQ6: What is the effect of the three types of visual stimuli on the users’ perception of the credibility of a COVID-related social media post?

Aside from the primary independent variable (type of visual stimuli), the study will record additional variables to serve as controls. These variables include the confirmation bias measure (due to the finding of Kim, Moravec & Dennis (2019) that it has effects on post-perception), age, and social media usage (both through time and activity).

## Methods

### Participants

Three hundred participants (40.7% female) completed the survey, collected through Amazon’s Mechanical Turk (MTurk) platform. Each participant in the study received a payment of $1.5. The average age of participants was 37.09 (ranging from 21 to 78, SD = 9.896). The country of the participants appears in Table 1. Participants were sampled using convenience sampling, as this was the only way to collect participants online during the pandemic within the resources available to the researcher.

**Table 1:** Absolute and relative frequency of participants from various countries.

Country	Frequency	Percent
United States	217	72.33
India	57	19
Saudi Arabia	11	3.67
Brazil	3	1
Romania	3	1
Germany	2	0.67
Italy	2	0.67
Singapore	1	0.33
United Kingdom	1	0.33
Missing	3	1
Total	300	100

### Design

An experimental design measured the effect that viewing each type of imagery has on participants. Before participating in the questionnaire, participants signed an online consent form. All participants looked at posts containing three types of visual stimuli: advisory, clickbait, or data visualization. Then, they gave estimates on their emotional state, perception of risk, engagement with the post, and its credibility. The creation of three parallel survey formats avoided confounding the effect of visuals with the headline content. The headlines were counterbalanced across three parallel survey forms by first choosing nine headlines and labelling them randomly from 1 to 9. Then, a random number generator created a random sequence of numbers 1 to 9. The design used the headlines corresponding to the first three numbers in the sequence for the three stimuli with generic COVID-19 imagery. It used the second three headlines for the three stimuli with informative, advisory imagery. It used the final three headlines for the three stimuli with data visualizations. Thus, it created the first parallel set of stimuli. Then, the Latin square method counterbalanced the headline sets across the remaining two parallel forms. The second form swapped the places of the three sets of headlines among the three groups of visual stimuli. The first set now used the informative, advisory imagery, the second used the data visualization stimuli, and the third used the generic COVID-19 imagery. The creation of the third parallel form utilized the same logic, with an additional step of swapping places. The first set of headlines now used the data visualization stimuli, the second used generic COVID-19 imagery, and the third used informative advisory imagery. This method ensured that the registered effect is from the types of imagery and not from the headlines. The process of counterbalancing appears in Table 2. As the table illustrates, all the headline sets appear together with each type of imagery exactly once.

**Table 2:** Headline sets used in each of the parallel forms of the surveys.

Type of imagery	First parallel form	Second parallel form	Third parallel form
Generic	Set 1	Set 3	Set 2
Advisory	Set 2	Set 1	Set 3
Data visualization	Set 3	Set 2	Set 1

## Materials

### Social media posts

**Figure 1:**
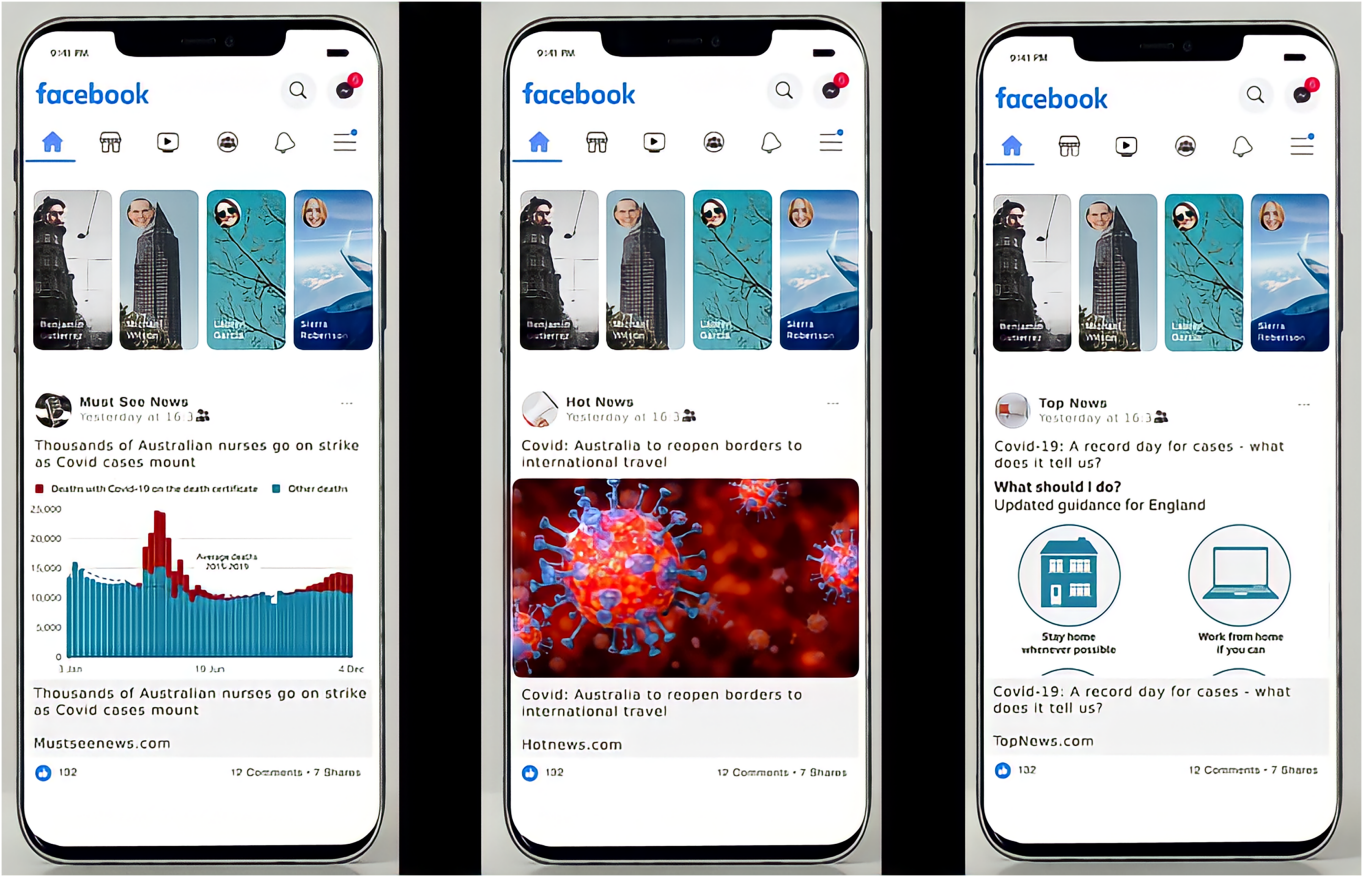
Examples of used imagery, including data visualization (A), clickbait (B), and advice (C).

When creating fake social media posts, there were two elements to consider: imagery and headlines. The imagery came from the following sources: advice imagery from the BBC’s webpage titled ‘Coronavirus information: Four posters’ (BBC, 2020a); data visualisations from the BBC’s webpage titled COVID: 12 charts on how COVID changed our lives (BBC, 2020b); and generic Coronavirus imagery (clickbait) from various articles posted by BBC. The headlines were from the BBC’s website to keep them as descriptive as possible without mention of any famous people. Examples of all three types of imagery appear in Fig. 1. The social media pages that posted the content were completely made-up, and only reflected the idea that they were posted by a news website, as has been done in previous studies (Kim, Moravec & Dennis, 2019). Although it is possible that the news website has an effect on credibility in the eyes of the participants, it was expected that all websites would have a similar effect. Furthermore, they were counterbalanced in the same manner as the titles (title-poster pairs were not switched when imagery was switched for the three parallel studies). As a result, there was no reason to expect that the website titles would have an effect on user perceptions that had not been controlled for by the study design. Mobile social media inspired the format of the posts, as studies show that most people use social media on their mobile phones. For example, in 2019, 79% of social media usage in the US was through mobile devices (Dixon, 2022).

### Self-assessment mannequin (SAM)

**Figure 2:**
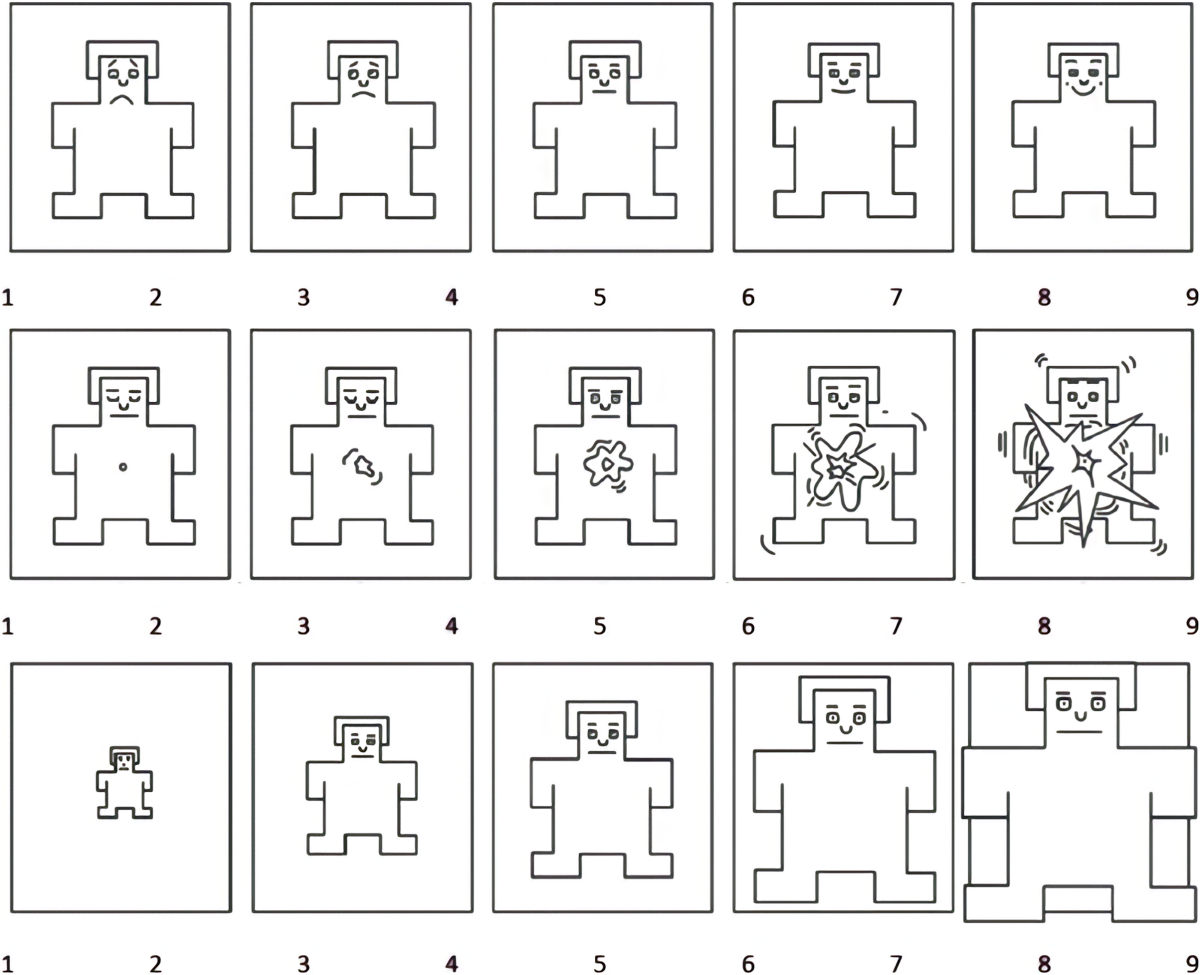
Self-assessment mannequin—valence, arousal, and dominance scale, in order. Figure source: © Elsevier, Bradley & Lang (1994).

This method used the self-assessment mannequin (Lang, 1980) to measure participants’ emotional states after looking at social media posts. It measures three dimensions of emotion—pleasure, arousal, and dominance (Bradley & Lang, 1994), using non-verbal estimation on a nine-point Likert-type scale. This assessment tool is widely used for quickly and reliably determining users’ reactions to technology, as it is informative for the researcher and easy to use for the participants (Dormann, 2001, 2003; Isomursu et al., 2007). The scale appears in Fig. 2.

### Risk perception

A new questionnaire measured participants’ risk perception after looking at social media posts. Previous literature contained various surveys measuring multiple types of risk perception, but none of them was fully applicable to the current research context. Therefore, we created a survey with five items, answered through a five-point Likert-type (ranging from 1 to 5) scale by adapting questions from previous studies (Alsubaie et al., 2019; Kim, Moravec & Dennis, 2019; Qin et al., 2021). The final scale showed excellent internal reliability (α ranging from 0.89 to 0.904). An example item is ‘After seeing this news post, how much anxiety do you feel about the COVID-19 pandemic?’.

### Post credibility

This method adapted a previously created survey by Janét, Richards & Landrum (2022) to measure participant perception of the credibility of social media posts. The survey consists of ten items, answered with a six-point Likert-type scale ranging from 1 (disagree) to 6 (strongly agree). An example item is ‘Based on this post, I expect that the content in the article…can be trusted.’ (The part before the ellipsis is the same for all questions, and the part after it differs for each item). The scale showed good reliability both in the study of Janét, Richards & Landrum (2022) (α = 0.88) and in the present study (α ranging from 0.781 to 0.815).

### Engagement

The survey of Janét, Richards & Landrum (2022) measured how much the participants engaged with the post. It asked participants whether they ‘would likely share the article, read the article, comment on the article, or ignore the article.’ The participants selected all that applied, and the number of activities (ranging from 0 to 3) was an indicator of engagement.

### COVID sentiment

A COVID sentiment index measured the participants’ bias towards COVID-related social media posts. The index took inspiration from previous research on cognitive bias (Kim, Moravec & Dennis, 2019) by asking the participants two questions: the first question, ‘What’s your opinion on news’ reportage on COVID-19?’, was answered on a scale between −3 and 3; the second question, ‘Do you find the COVID-19 pandemic important?’ was answered on a scale ranging from 1 to 7. By multiplying the two answers, an index which simultaneously indicates how strongly and in what direction a participant feels about the pandemic was created. The index ranged from −21 to 21.

## Procedure

**Figure 3:**
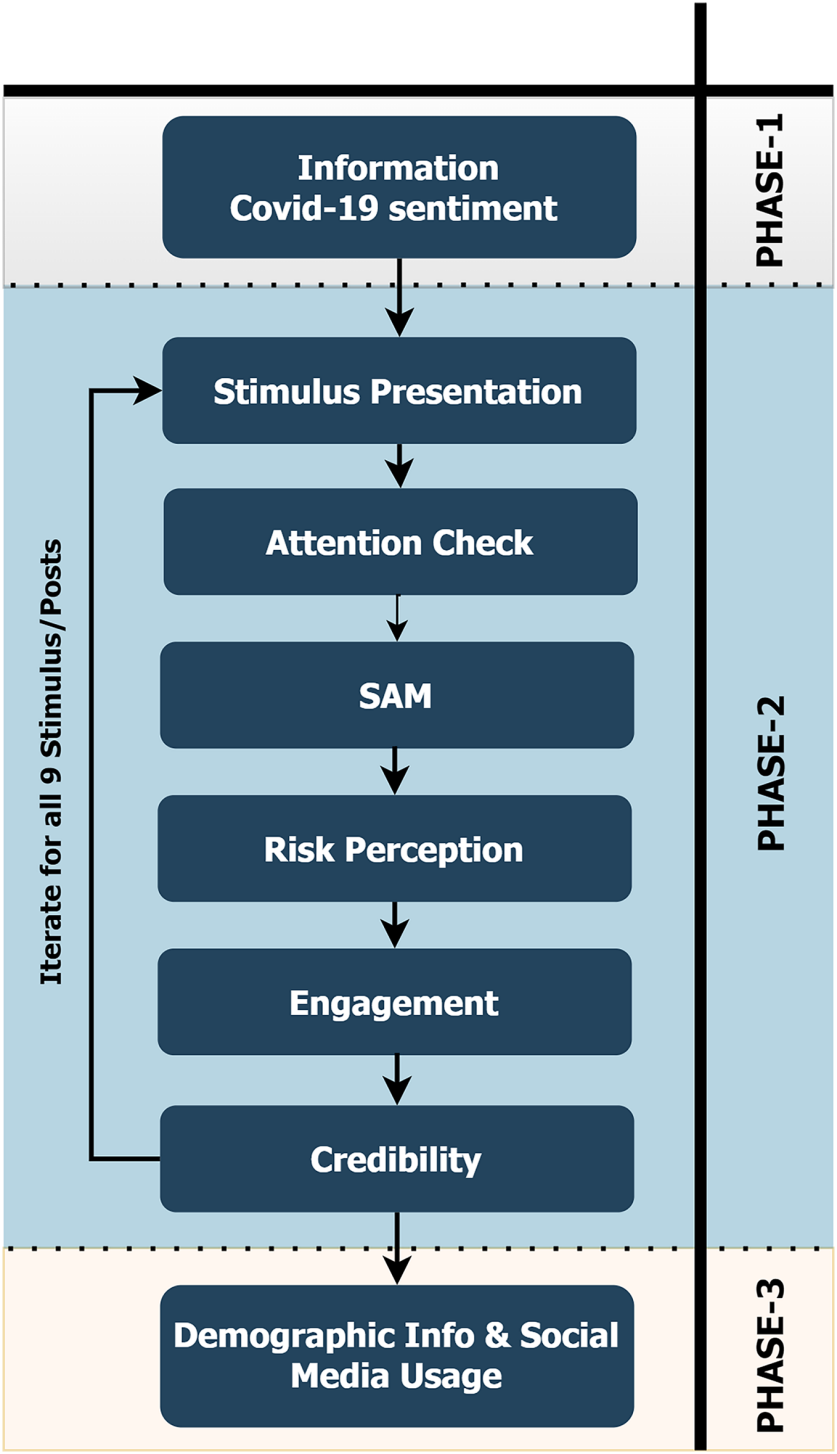
Diagram representing the process of taking the survey.

The researcher followed the standard ethics procedures throughout this study. Initially, participants have been thanked by the researcher for taking part in the questionnaire and informed them about the details of the study. Participants signed an online consent form. They learned that their information will not be analysed individually but only at a group level, and their data will be treated confidentially and anonymously. The researcher informed them that they were free to quit taking the survey at any point and received a contact e-mail address of the researcher if they had any inquiries. Then, they answered questions regarding their sentiment toward COVID-19 news reporting (see the materials section). Afterwards, they received a (fake) social media post, which they were instructed to view carefully. The next screen presented a question which tested whether they were paying attention to the stimulus. This question was an attention check, and participants who wrongly answered four or more questions were discarded from subsequent data analysis. There were 55 such cases, reducing the final sample to 245 participants. After the attention check, the participants answered the SAM, credibility, risk perception, and engagement surveys about the stimulus. Then, this process repeated for the following eight stimuli. After finishing all nine stimuli, they answered questions about their demographic information and social media usage. In the end, they were thanked for their participation. The whole process through which the participants went appears in Fig. 3.

## Results

### Descriptive statistics

Brief descriptive statistics of the six main study variables appear in Table 3. Kurtosis values denote negative excess kurtosis. Valence and arousal scores were on average only slightly higher than the middle value of the SAM (5). Dominance was in the same range, albeit slightly higher than the other two. The standard errors and standard deviations of all three variables were comparable. Furthermore, risk perception was slightly elevated, with the mean answer nearly a full point above the theoretical middle (2.5). It also showed low standard deviation and standard error. The mean of engagement indicates that it is exactly in line with the expected average of 1.5, with comparably slightly higher SE and SD, in comparison to risk. Finally, credibility also showed an expected mean close to the theoretical (3.5), with very low standard deviation and standard error values. The three SAM dimensions show similar means and standard deviations, and their distributions are skewed to the left and platykurtic. An exception is the dominance dimension, which is mesokurtic. Furthermore, risk perception is skewed to the left and mesokurtic, engagement is slightly skewed to the left and platykurtic, and credibility is skewed to the left and platykurtic as well. These deviations from the normal distribution agree with both Kolmogorov–Smirnoff and Shapiro–Wilk tests, which indicated significant deviations for all six variables (all *p* < 0.001).

**Table 3:** Descriptive statistics of the six main study variables.

Variable	M	SE (M)	SD	SE (SD)	Skewness (SE)	Kurtosis (SE)
Valence	5.51	0.073	1.98	0.089	−0.169 (0.090)	−0.648 (0.180)
Arousal	5.55	0.068	1.86	0.084	−0.167 (0.090)	−0.566 (0.180)
Dominance	6.00	0.072	1.97	0.089	−0.543 (0.090)	−0.282 (0.180)
Risk	3.35	0.034	0.93	0.042	−0.674 (0.090)	−0.139 (0.180)
Engagement	1.53	0.036	0.98	0.044	0.203 (0.090)	−01.066 (0.180)
Credibility	3.56	0.021	0.56	0.025	−0.597 (0.090)	1.949 (0.180)

The reliability of the three types of stimuli was determined using Cronbach’s alpha coefficients. Cronbach’s alpha coefficients across the three stimuli for each of the three types of stimuli were above 0.8, as shown in Table 4. This result indicates that the types of stimuli were similar enough inside the clusters of stimuli and could reliably be measured against other variables.

**Table 4:** Cronbach’s alpha across three stimuli for each stimuli type.

Variable	Clickbait	Visual	Advice
Valence	0.827	0.905	0.874
Arousal	0.856	0.869	0.877
Dominance	0.879	0.899	0.840
Risk	0.916	0.946	0.933
Engagement	0.816	0.941	0.946
Credibility	0.942	0.806	0.882

### Results of general linear mixed models

The effects of the primary variable of interest, the type of visual material used in the thumbnails, along with the remaining predictors, including age, sentiment towards COVID-19, hours on social media, and activity on social media, will be investigated using general linear mixed models. Due to differences in some of the dependent variables based on country of origin, this variable was included in the models as well, but only as a control variable, and will not be interpreted, as this study did not aim to measure cultural differences. The researcher chose this model instead of mixed ANOVA models due to its higher robustness and customizability.

The goal of the present article was not to investigate the interactions of the visual type of thumbnails with the remaining predictors. Instead, it tests for its effect when the other variables are controlled. Therefore, the general linear mixed models were deemed more suitable. As it is unlikely that either of the between-subject variables varies inside the subjects, all of them were used as fixed effects. Furthermore, the visual type of covariance structure was determined as unstructured, as it is natural to expect high covariance among the different types of visual stimuli, and there is no reason to assume a specific pattern in which that covariance will occur. The overall significant fixed effects of each of the six criterion variables appear in Table 5. Subsequent sub-sections will investigate each of these predictors in further detail.

**Table 5:** Results of tests of fixed effects for all six criterion variables.

Predictor variables	Valence		Arousal		Dominance		Risk		Engagement		Credibility	
	F	Sig.	F	Sig.	F	Sig.	F	Sig.	F	Sig.	F	Sig.
Intercept	225.950	0.000	210.026	0.000	261.755	0.000	332.494	0.000	35.432	0.000	625.947	0.000
Visual type	19.343	0.000	4.301	0.014	14.708	0.000	11.079	0.000	0.749	0.474	12.283	0.000
Hours per day on SM	0.920	0.527	1.002	0.447	0.624	0.821	2.341	0.007	1.160	0.312	0.502	0.913
Activity on SM	11.583	0.000	16.931	0.000	11.077	0.000	18.009	0.000	17.652	0.000	3.111	0.016
Age	2.281	0.132	0.088	0.767	3.171	0.076	0.952	0.330	0.730	0.394	0.693	0.406
COVID sentiment	31.761	0.000	18.562	0.000	18.013	0.000	20.962	0.000	6.276	0.013	13.897	0.000
Country	4.858	0.028	1.496	0.222	0.013	0.909	0.586	0.445	0.090	0.764	0.067	0.0795

#### Valence

As seen in Table 5, the valence of the reaction to the different types of visual stimuli has been successfully predicted by the type of stimulus, social media activity, and COVID sentiment. Pairwise comparisons using the Bonferroni correction showed that all pairwise differences among the visual types were significant. The advice thumbnail type had higher valence than both the bait (MD = 0.186, *p* = 0.026) and the visual (MD = 0.380, *p* < 0.001) thumbnails, and the bait had higher valence than the visual (MD = 0.380, *p* < 0.001) thumbnail. Furthermore, valence had been positively affected by activity on social media and sentiment towards COVID, indicating that participants who were more active on social media and had a more positive sentiment towards the pandemic reporting also experienced higher valence while observing the social media posts.

#### Arousal

As Table 5 shows, the arousal of the reaction to the different types of visual stimuli has been successfully predicted by the type of stimulus, social media activity, and COVID sentiment. Pairwise comparisons using the Bonferroni correction showed that advice thumbnails yielded higher arousal than both bait (MD = 0.171, *p* = 0.035) and visual (MD = 0.192, *p* = 0.024) types. There were no differences between bait and visual types of visuals (*p* = 1). Arousal was positively affected by activity on social media as well as sentiment towards COVID. This result indicates that participants who were more active on social media and had a more positive sentiment towards the pandemic reporting also experienced higher arousal while observing the social media posts.

#### Dominance

As Table 5 shows, the experienced dominance upon viewing the different types of visual stimuli has been successfully predicted by the type of stimulus, social media activity, and COVID sentiment. Participants experienced higher dominance upon viewing the advice thumbnail, in comparison to both the bait (MD = 0.254, *p* = 0.001) and the visual (MD = 0.453, *p* < 0.001) thumbnails, while the bait thumbnail yielded a higher effect on dominance than the visual (MD = 0.199, *p* = 0.011) thumbnail. Dominance had been positively affected by activity on social media and sentiment towards COVID. This result indicates that participants who were more active on social media and had a more positive sentiment towards the pandemic reporting also experienced higher dominance while observing the social media posts.

#### Risk perception

Table 5 shows the perception of risk upon viewing the different types of visual stimuli has been successfully predicted by the type of stimulus, social media activity, hours spent on social media, and COVID sentiment. Participants estimated risk as higher upon viewing the visual thumbnail, in comparison to both the bait (MD = 0.099, *p* < 0.001) and the advice (MD = 0.122, *p* < 0.001) thumbnails, while there was no significant difference between the bait and the advice (MD = 0.022, *p* = 1) thumbnail. Activity on social media, hours spent on social media, and sentiment towards COVID had positive effects on risk perception. This result indicates that participants who were more active on social media, spent more time on social media, and had a more positive sentiment towards the pandemic reporting also experienced higher dominance while observing the social media posts.

#### Engagement

As Table 5 shows, engagement with posts, including the different types of visual stimuli, has been successfully predicted by social media activity and COVID sentiment. Both this activity on social media and sentiment towards COVID had positive effects on engagement. This result indicates that participants who were more active on social media and had a more positive sentiment towards the pandemic reporting also engaged more with COVID-related social media content.

#### Credibility

Table 5 shows the perception of the credibility of posts containing the different types of visual stimuli has been successfully predicted by the type of stimulus, activity on social media, and COVID sentiment. Participants estimated credibility as higher upon viewing the advice thumbnail, in comparison to both the bait (MD = 0.106, *p* < 0.001) and the visual (MD = 0.117, *p* < 0.001) thumbnails, while there was no significant difference between the bait and the visual (MD = 0.083, *p* = 1) thumbnail. Both activity on social media and sentiment towards COVID had positive effects on risk perception. This result indicates that participants who were more active on social media and had a more positive sentiment towards the pandemic reporting also perceived articles related to COVID as more credible.

### Results summary

In summary, the participants seemed to have the most pleasant experience while observing the advisory visuals in the social media posts related to COVID-19. These posts yielded higher valence, arousal, dominance, credibility, and lower risk perception. Visual stimuli were not preferred, with the highest perception of risk and lower valence and dominance than the other types of stimuli. The clickbait type of visual stimulus was most commonly in the middle between the other two, never being the one with the highest or the lowest scores on a measure. Furthermore, the visual type of stimuli was not relevant to the engagement at all. Lastly, activity on social media and sentiment towards COVID-19 successfully predicted all criteria, indicating that they are relevant for the emotions, risk perception, engagement, and credibility estimation of social media posts relating to COVID-19.

## Discussion

This study investigated the effect of different types of visual stimuli in a COVID-related social media post on users’ reactions to that post. The study postulated six research questions to assess this effect, and the following part of the discussion will examine them one by one. Then, it will present an integration of all results along with the implications of the findings. This discussion will conclude with the study’s limitations.

RQ1: What is the effect of the three types of visual stimuli on the valence of emotions that users feel after looking at a COVID-related social media post?

The answer to the first research question is that advisory visuals yielded the most positive emotional reactions, while the data visualization type yielded the most negative emotions. Clickbait-type visuals were between these two in this regard. This result may be a consequence of the data visualization stimuli leading the users to perceive the most risk, which scares them and initiates negative emotions. The advisory stimuli may simply be more pleasant to observe than the clickbait imagery, thus making them feel more positive. Aside from visual stimuli type, this study demonstrated that activity on social media and COVID-19 sentiment affect valence as well. The effect of the COVID-19 sentiment is likely a confirmation bias, with people who have more positive views on COVID-19 coverage and find it more important to have more positive reactions towards COVID-related news stories, similar to the findings of Kim, Moravec & Dennis (2019). Furthermore, people who are more active on social media websites also experienced higher valence, which is likely due to a higher preference for viewing social media material, which leads them to feel higher valence in this study.

RQ2: What is the effect of the three types of visual stimuli on the arousal that users feel after looking at a COVID-related social media post?

The results of the study regarding arousal were similar to those regarding valence. Advisory imagery made the participants feel more aroused, and there were no differences in arousal between the other two types of imagery. Thus, advice on what to do during the pandemic seems to put the participants in a more awake, alert state, potentially by making them imagine themselves doing the recommended actions. Furthermore, like the previous topic, activity positively affected social media and COVID sentiment. The explanation for these factors may be the same as the previous question.

RQ3: What is the effect of the three types of visual stimuli on the dominance that users feel after looking at a COVID-related social media post?

Dominance exhibited an identical pattern of differences in the three types of imagery to valence: advisory imagery yielded the highest dominance, followed by bait, and data visualization in last place. This order is also logical, as the advisory imagery gives the participant a sense of control or effect on their surroundings. On the contrary, data visualizations show large numbers of people affected by something, which may make the person feel tiny and out of control. As SAM is a non-verbal measurement, it may be that the advice, which puts them in the ‘first plan’ made them feel bigger, while the data put them in the background, making them feel smaller. Furthermore, feeling more positively toward COVID-19 reporting and spending more time on social media also had positive effects on dominance, likely due to feeling more in control in this specific situation of looking at a COVID-related social media post. People who look at social media posts more frequently will logically feel more dominant when looking at another one, because they are doing something they do frequently and confidently. The same is the case for those who feel positively towards COVID and are used to reading social media posts about it.

RQ4: What is the effect of the three types of visual stimuli on the users’ perception of risk after looking at a COVID-related social media post?

Partially in line with the interpretation given for RQ1, the participants felt the most risk after looking at visualization-type imagery. As the introduction discussed, this type of imagery likely addresses risks explicitly by giving the users specific numbers (or visuals) which show how at-risk they, or some other people, are. Thus, it was natural that this type of imagery yielded the highest perception of risk. On the other hand, the remaining two types of thumbnails showed no differences in risk perception between them. This result is also in line with the findings on dominance, as the riskiest type of imagery is one in which the participants feel the least dominant. Previous research indicated that imagery in social media posts might be important for risk perception (Bica et al., 2019), which is in line with the findings of this study.

Those who are more active on social media and have a more positive sentiment towards COVID showed higher risk perception. This result indicates that they may trust social media more, as the credibility results have confirmed, which will be presented later. Since these participants trust the news on social media more, they take it more seriously and experience a higher perception of risk when interacting with it because it deals with the pandemic.

RQ5: What is the effect of the three types of visual stimuli on the users’ engagement with a COVID-related social media post?

Engagement is the only study variable on which the types of imagery had no effect. The reason may be that the content of the social media post may be the deciding factor whether a person interacts with the post or not and not the visuals. Previous studies did show that altering the types of headlines changes engagement in various ways (Janét, Richards & Landrum, 2022), but instead of trying to manipulate the headlines, this study kept the headlines under control. Thus, the only recorded effects on engagement were those of social media activity, which is a measure of typical engagement, and of COVID sentiment, which may be another instance of confirmation bias, in which people who feel more positively towards something feel more inclined to interact with it. The lack of differences in engagement despite changes in arousal and other aspects of emotions may be due to the method by which the engagement was measured. The score on engagement measures the number of ways in which a participant is willing to engage with the content, rather than their likelihood of engaging in general. As a result, it is possible that the measure was not sensitive enough to detect the subtle differences in users’ likelihood of engaging based on the type of visual imagery and potentially provoked by their differences in emotional states.

RQ6: What is the effect of the three types of visual stimuli on the users’ perception of the credibility of a COVID-related social media post?

The answer to the final research question is that advice seemed more credible than both clickbait and data visualization. This type of visualization may be the most straightforward, similar to how the traditional headlines were seen as the most credible in the study of Janét, Richards & Landrum (2022). Their study also showed that agreeing with the topic of the post was instrumental in seeing it as more credible. This study arrived at the same findings due to the effect of COVID sentiment on credibility perception.

In sum, the results paint a clear picture: advisory thumbnails make the users the most positive, aroused, in control, have them not feel too much risk, and believe in the credibility of a social media COVID-related post the most. On the other hand, visual data imaging builds risk perception, negative emotions, a lack of control, and inferior credibility. Clickbait thumbnails fall mostly in between the other two. All these effects are persistent beyond the numerous control variables included in the models. This result is consistent with previous literature indicating that different aspects of social media posts have systematic effects on the audience (Bica et al., 2019; Ghanem, Rosso & Rangel, 2020; Janét, Richards & Landrum, 2022; Kim, Moravec & Dennis, 2019; Kim & Dennis, 2019).

Such outcomes may be due to the nature of these types of imagery—advisory imagery may provide users with a sense of control, or at least controllability. They are instructed to do certain activities that will benefit them, or at least keep them risk-free. That could explain why they feel more positively, more aroused, in control, and perceive risk as lower. They may also see the posts as more credible because they appear to be reasonable advice from relevant authorities. Data presentation imagery, on the other hand, has the opposite effect, which can be explained by its nature. When looking at large numbers, people may feel less like they have any effect. Therefore, they feel worse, less aroused, less in control, and perceive a high level of risk—they are afraid and helpless. Furthermore, the lower credibility may be due to common statistical manipulation in the media. These interpretations are slight exaggerations of reality, with only minor differences in scores, but they explain the likely mechanisms by which the differences arose.

Health officials and others who want to share information regarding COVID-19 on social media would be wise to utilize advisory thumbnails, to keep their participants feeling as positive as possible and enhance their credibility. On the other hand, if their wish is to scare the user and make them realize that they are in danger and that they do not have control over a situation, they should opt for various forms of data visualization. Future studies may want to integrate the investigations of headlines by Janét, Richards & Landrum (2022). with examinations of imagery to determine the optimal conditions of information delivery in terms of how the users respond to it.

This study had two limitations. The first one was the usage of convenience sampling. The researcher’s resources did not allow for random sampling, which would be more representative of the population of social media users. However, there is no reason to believe that convenience sampling yielded a biased sample relevant to the study. Another limitation is that the study was not conducted in a naturalistic context—the participants did not observe the social media posts during their usual social media usage. Furthermore, the experiments in this study rely on questionnaires to assess the participants’ perception of social media posts. As a result, the findings are less credible than those of objective data analyses or analyses of actual social media posts. However, it is impossible to systematically alter the thumbnail content while controlling for other factors using more naturalistic designs, which is why this study used the experimental approach. It maximized the ecological validity by replicating the exact look of Facebook social media posts.

## Conclusions

This study investigated the effects of different types of thumbnails of COVID-related social media news posts on users’ reactions. It determined that advisory imagery positively affected valence, arousal, dominance, credibility, and (lower) risk perception. Furthermore, imagery showing data visualizations yielded low valence, arousal, dominance, credibility, and high risk perception. Because of the low emotionality and high risk perception, data visualizations may be best suited for calmly warnings users of high risk situations. Clickbait-styled thumbnails which carry no information tended to fall between the other two types. The type of imagery did not affect the motivation to engage with a post. Aside from visual imagery, this study showed that most variables were affected by COVID sentiment and usual activity on social media. These results can help health officials and other users utilize imagery to deliver information about the COVID pandemic or other crises to cause specific emotions in the users.

## Supplemental Information

10.7717/peerj-cs.1153/supp-1 Supplemental Information 1 Dataset of Survey 1.

10.7717/peerj-cs.1153/supp-2 Supplemental Information 2 Dataset of Survey 2.

10.7717/peerj-cs.1153/supp-3 Supplemental Information 3 Dataset of Survey 3.

10.7717/peerj-cs.1153/supp-4 Supplemental Information 4 Questionnaire Form 1.

10.7717/peerj-cs.1153/supp-5 Supplemental Information 5 Questionnaire Form 2.

10.7717/peerj-cs.1153/supp-6 Supplemental Information 6 Questionnaire Form 3.

## Additional Information and Declarations

### Data Availability

The following information was supplied regarding data availability: The data is available at figshare: Al-nuwaiser, Waleed (2022): Effect of visual imagery in COVID-19 social media posts on users’ perception. figshare. Dataset. https://doi.org/10.6084/m9.figshare.20627823.v2.
