# Supplementary material for: Effect of visual imagery in COVID-19 social media posts on users’ perception
Source: PeerJ Comput Sci. 2022 Nov 15;8:e1153. doi: 10.7717/peerj-cs.1153 (PMC9680878; doi:10.7717/peerj-cs.1153)
Supplement: Supplemental Information 4 [file peerj-cs-08-1153-s004.zip › PS1_Survey+Stimuli/survey_90434446_PS1.pdf]

SocialMediaRiskPerception-1

Survey Map

This survey is collecting data from you concerning the personal risk associated with browsing mobile-size social media posts about Covid-19. All questions are in the form of multiple-choice**.**

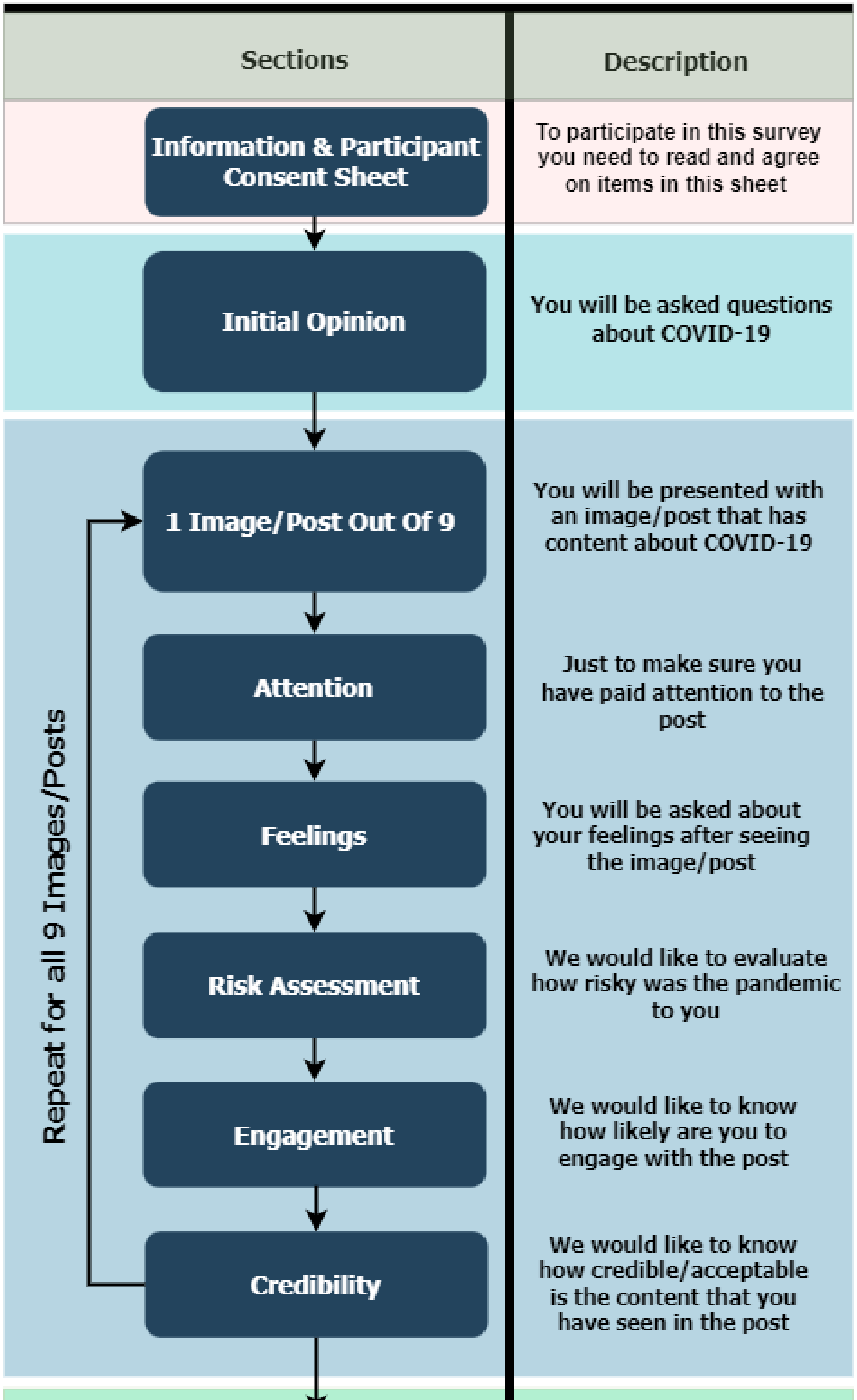

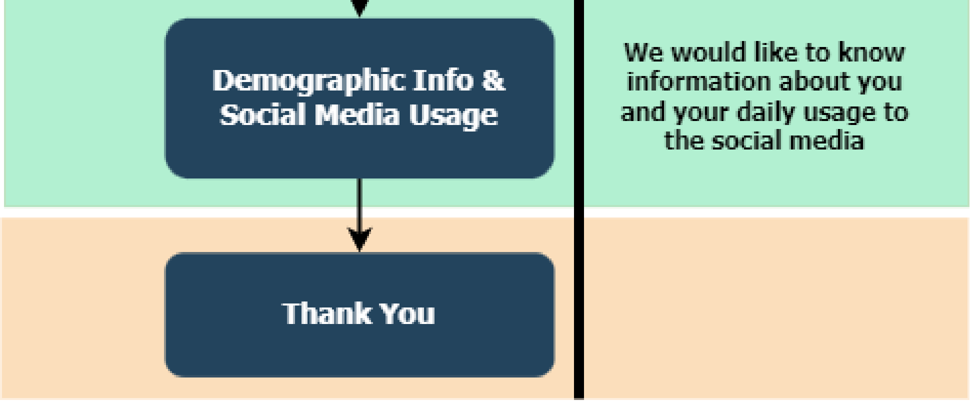

Information and Participant Consent Sheet

Please tick each point when you read it and agree on it: *

- ☐ **Purpose of the research:** Evaluating the associated personal risk when browsing mobile-size social media posts about Covid-19
- ☐ **Your role in this research:** if you decide to participate, you will complete a questionnaire based on your experience during the pandemic of Covid-19
- ☐ **Time Required:** The survey will take approximately 15 - 20 minutes to complete
- ☐ **Confidentiality:** Your responses will be kept anonymous. When research results are reported, responses will be aggregated (added together) and described in summary, with no single individual identifiable
- ☐ **Participation and Withdrawal:** Your participation would be highly appreciated. if you decide to participate, and you complete the survey, at the completion of the survey, you will get a reward code. You may refuse to participate or quite at any time and it is completely your choice
- ☐ **Repetition:** You may find some of the questions repetitive. However, due to the purpose of the research all questions must be answered
- ☐ **Attention:** You will be presented with 9 images/posts. Your attention and having a detailed look are required on each image/post before you proceed with the subsequent pages.

To contact the researcher: if you have any question or concerns about this research, please contact: Waleed Alnuwaiser, Email: waleednw@gmail.com

Initial Opinion Assessment

Do you find the COVID-19 pandemic important? *

	1	2	3	4	5	6	7	
**Not at all**	○	○	○	○	○	○	○	**Extremley**

What is your opinion on news' reportage on COVID-19? *

	-3	-2	-1	0	+1	+2	+3	
**Extremely Negative**	○	○	○	○	○	○	○	**Extremely Positive**

Have you been vaccinated against COVID-19? *

Yes	No
○	○

Image/Post

Please have a detailed look at the following image/post and tick 'I am done' when you finish:
*

- ☐ I am done

Attention test

Which human organ was presented in the last picture? *

- ○ Lungs
- ○ Heart
- ○ Stomach
- ○ Kidneys

Feelings

Rate how positive or negative this post made you feel, ranging from **'sad'** to **'happy'**:

*

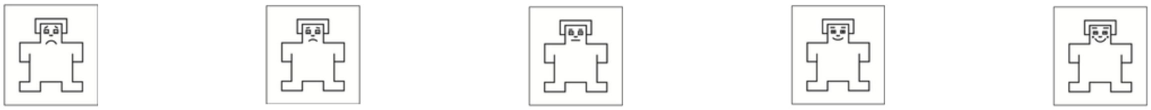

	1	2	3	4	5	6	7	8	9	
**Sad**	○	○	○	○	○	○	○	○	○	**Happy**

Rate how energetic this post made you feel, ranging from **'sleepy/apathetic'** to **'excited/energetic' :** *

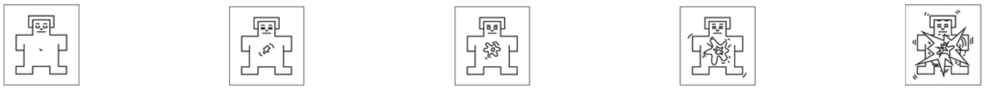

	1	2	3	4	5	6	7	8	9	
**Sleepy/Apathetic**	○	○	○	○	○	○	○	○	○	**Excited**

Rate how in control of the situation this post made you feel, ranging from **'no control'** to **'completely in control':** *

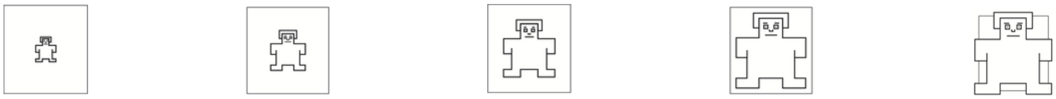

	1	2	3	4	5	6	7	8	9	
**No control**	○	○	○	○	○	○	○	○	○	**Completely in control**

Risk Assessment

After seeing this news post, how much anxiety do you feel in regards to the COVID-19 pandemic?*

	1	2	3	4	5	
**No anxiety at all**	○	○	○	○	○	**Very high anxiety**

After seeing the news post, how big of a risk do you think the COVID-19 pandemic poses to you or your family?*

	1	2	3	4	5	
**No risk at all**	○	○	○	○	○	**Very high risk**

Where ‘dread’ means to be in terror of, or fear intensely, how much do you dread the COVID-19 pandemic after seeing this news post?
*

	1	2	3	4	5	
**No dread at all**	○	○	○	○	○	**Very high dread**

After seeing the news post, what do you think is your likelihood of infection?

	1	2	3	4	5	
**Very unlikely**	○	○	○	○	○	**Very likely**

After seeing the news post, how harmful do you think it would be if you got infected?*

	1	2	3	4	5	
**Not harmful at all**	○	○	○	○	○	**Very harmful**

Engagement

Are you likely to… (check where applicable)?*

- ☐ Share the post
- ☐ Read the post
- ☐ Comment on the post
- ☐ Ignore the post

Credibility

... can be trusted *

	1	2	3	4	5	
**Strongly disagree**	○	○	○	○	○	**Strongly agree**

...separates facts from opinion *

	1	2	3	4	5	
**Strongly disagree**	○	○	○	○	○	**Strongly agree**

...is factual *

	1	2	3	4	5	
**Strongly disagree**	○	○	○	○	○	**Strongly agree**

...tells the whole strory *

	1	2	3	4	5	
**Strongly disagree**	○	○	○	○	○	**Strongly agree**

...is accurate *

	1	2	3	4	5	
**Strongly disagree**	○	○	○	○	○	**Strongly agree**

...is unbiased *

	1	2	3	4	5	
**Strongly disagree**	○	○	○	○	○	**Strongly agree**

...is fair *

	1	2	3	4	5	
**Strongly disagree**	○	○	○	○	○	**Strongly agree**

... demonstrates concern about public interest *

	1	2	3	4	5	
**Strongly disagree**	○	○	○	○	○	**Strongly agree**

...is sensationalized *

	1	2	3	4	5	
**Strongly disagree**	○	○	○	○	○	**Strongly agree**

...is immoral *

	1	2	3	4	5	
**Strongly disagree**	○	○	○	○	○	**Strongly agree**

Image/Post

Please have a detailed look at the following image/post and tick 'I am done' when you finish:
*

☐ I am done

Attention test

Which colors were presented in the last picture? *

- ○ Red and Blue/Gey
- ○ Yellow and Red
- ○ Black and White
- ○ Purple and Pink

Feelings

Rate how positive or negative this post made you feel, ranging from**'sad'** to **'happy'**:

*

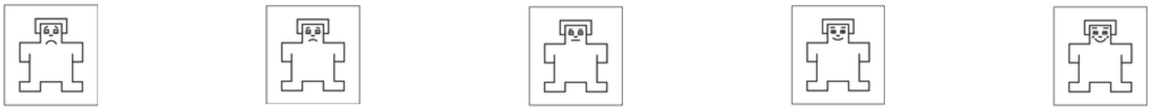

	1	2	3	4	5	6	7	8	9	
**Sad**	○	○	○	○	○	○	○	○	○	**Happy**

Rate how energetic this post made you feel, ranging from **'sleepy/apathetic'** to **'excited/energetic'** : *

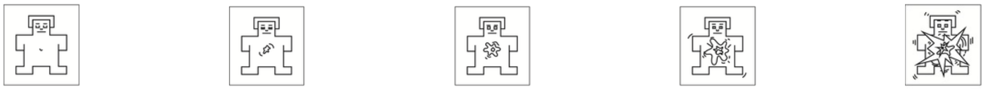

	1	2	3	4	5	6	7	8	9	
**Sleepy/Apathetic**	○	○	○	○	○	○	○	○	○	**Excited**

Rate how in control of the situation this post made you feel, ranging from **'no control'** to **'completely in control'**: *

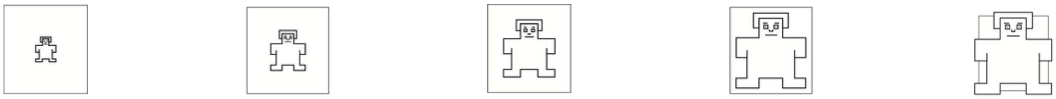

	1	2	3	4	5	6	7	8	9	
**No control**	○	○	○	○	○	○	○	○	○	**Completely in control**

Risk Assessment

After seeing this news post, how much anxiety do you feel in regards to the COVID-19 pandemic?*

	1	2	3	4	5	
**No anxiety at all**	○	○	○	○	○	**Very high anxiety**

After seeing the news post, how big of a risk do you think the COVID-19 pandemic poses to you or your family?*

	1	2	3	4	5	
**No risk at all**	○	○	○	○	○	**Very high risk**

Where 'dread' means to be in terror of, or fear intensely, how much do you dread the COVID-19 pandemic after seeing this news post?
*

	1	2	3	4	5	
**No dread at all**	○	○	○	○	○	**Very high dread**

After seeing the news post, what do you think is your likelihood of infection?*

	1	2	3	4	5	
**Very unlikely**	○	○	○	○	○	**Very likely**

After seeing the news post, how harmful do you think it would be if you got infected?*

	1	2	3	4	5	
**Not harmful at all**	○	○	○	○	○	**Very harmful**

Engagement

Are you likely to… (check where applicable)?*

- ☐ Share the post
- ☐ Read the post
- ☐ Comment on the post
- ☐ Ignore the post

Credibility

... can be trusted *

	1	2	3	4	5	
**Strongly disagree**	○	○	○	○	○	**Strongly agree**

...separates facts from opinion *

	1	2	3	4	5	
**Strongly disagree**	○	○	○	○	○	**Strongly agree**

...is factual *

	1	2	3	4	5	
**Strongly disagree**	○	○	○	○	○	**Strongly agree**

...tells the whole strory *

	1	2	3	4	5	
**Strongly disagree**	○	○	○	○	○	**Strongly agree**

...is accurate *

	1	2	3	4	5	
**Strongly disagree**	○	○	○	○	○	**Strongly agree**

...is unbiased *

	1	2	3	4	5	
**Strongly disagree**	○	○	○	○	○	**Strongly agree**

...is fair *

	1	2	3	4	5	
**Strongly disagree**	○	○	○	○	○	**Strongly agree**

... demonstrates concern about public interest *

	1	2	3	4	5	
**Strongly disagree**	○	○	○	○	○	**Strongly agree**

...is sensationalized *

	1	2	3	4	5	
**Strongly disagree**	○	○	○	○	○	**Strongly agree**

...is immoral *

	1	2	3	4	5	
**Strongly disagree**	○	○	○	○	○	**Strongly agree**

Image/Post

Please have a detailed look at the following image/post and tick 'I am done' when you finish:
*

☐ I am done

Attention test

What was the color of the spikes in the last picture? *

- ○ Green
- ○ Yellow
- ○ Blue
- ○ Black

Feelings

Rate how positive or negative this post made you feel, ranging from **'sad'** to **'happy'**:

*

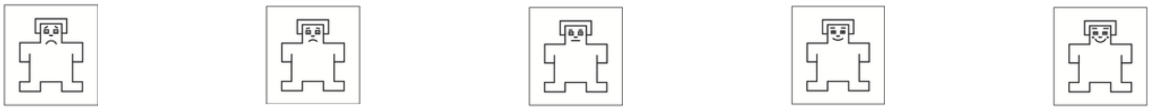

	1	2	3	4	5	6	7	8	9	
**Sad**	○	○	○	○	○	○	○	○	○	**Happy**

Rate how energetic this post made you feel, ranging from **'sleepy/apathetic'** to **'excited/energetic' :** *

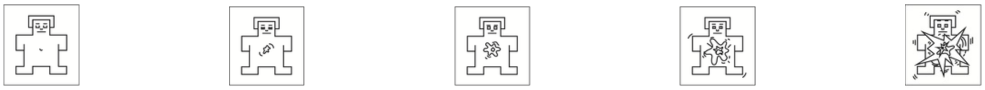

	1	2	3	4	5	6	7	8	9	
**Sleepy/Apathetic**	○	○	○	○	○	○	○	○	○	**Excited**

Rate how in control of the situation this post made you feel, ranging from **'no control'** to **'completely in control'**: *

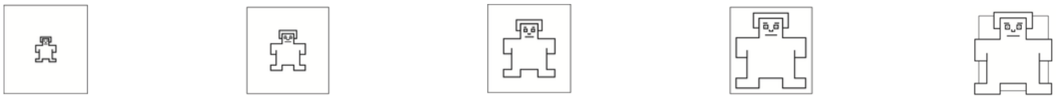

	1	2	3	4	5	6	7	8	9	
**No control**	○	○	○	○	○	○	○	○	○	**Completely in control**

Risk Assessment

After seeing this news post, how much anxiety do you feel in regards to the COVID-19 pandemic?*

	1	2	3	4	5	
**No anxiety at all**	○	○	○	○	○	**Very high anxiety**

After seeing the news post, how big of a risk do you think the COVID-19 pandemic poses to you or your family?*

	1	2	3	4	5	
**No risk at all**	○	○	○	○	○	**Very high risk**

Where 'dread' means to be in terror of, or fear intensely, how much do you dread the COVID-19 pandemic after seeing this news post?
*

	1	2	3	4	5	
**No dread at all**	○	○	○	○	○	**Very high dread**

After seeing the news post, what do you think is your likelihood of infection?*

	1	2	3	4	5	
**Very unlikely**	○	○	○	○	○	**Very likely**

After seeing the news post, how harmful do you think it would be if you got infected?*

	1	2	3	4	5	
**Not harmful at all**	○	○	○	○	○	**Very harmful**

Engagement

Are you likely to… (check where applicable)? *

- ☐ Share the post
- ☐ Read the post
- ☐ Comment on the post
- ☐ Ignore the post

Credibility

... can be trusted *

	1	2	3	4	5	
**Strongly disagree**	○	○	○	○	○	**Strongly agree**

...separates facts from opinion *

	1	2	3	4	5	
**Strongly disagree**	○	○	○	○	○	**Strongly agree**

...is factual *

	1	2	3	4	5	
**Strongly disagree**	○	○	○	○	○	**Strongly agree**

...tells the whole strory *

	1	2	3	4	5	
**Strongly disagree**	○	○	○	○	○	**Strongly agree**

...is accurate *

	1	2	3	4	5	
**Strongly disagree**	○	○	○	○	○	**Strongly agree**

...is unbiased *

	1	2	3	4	5	
**Strongly disagree**	○	○	○	○	○	**Strongly agree**

...is fair *

	1	2	3	4	5	
**Strongly disagree**	○	○	○	○	○	**Strongly agree**

... demonstrates concern about public interest *

	1	2	3	4	5	
**Strongly disagree**	○	○	○	○	○	**Strongly agree**

...is sensationalized *

	1	2	3	4	5	
**Strongly disagree**	○	○	○	○	○	**Strongly agree**

...is immoral *

	1	2	3	4	5	
**Strongly disagree**	○	○	○	○	○	**Strongly agree**

Image/Post

Please have a detailed look at the following image/post and tick 'I am done' when you finish:
*

☐ I am done

Attention test

For what amount of time did the picture advice you to wash hands? *

- 10 seconds
- 20 seconds
- 40 seconds
- 60 seconds

Feelings

Rate how positive or negative this post made you feel, ranging from **'sad'** to **'happy'**:

*

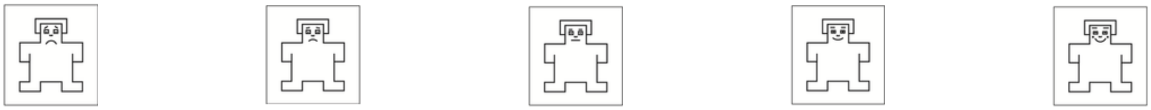

	1	2	3	4	5	6	7	8	9	
**Sad**	○	○	○	○	○	○	○	○	○	**Happy**

Rate how energetic this post made you feel, ranging from **'sleepy/apathetic'** to **'excited/energetic'** **:** *

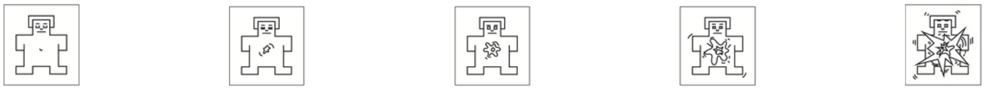

	1	2	3	4	5	6	7	8	9	
**Sleepy/Apathetic**	○	○	○	○	○	○	○	○	○	**Excited**

Rate how in control of the situation this post made you feel, ranging from **'no control'** to **'completely in control'**: *

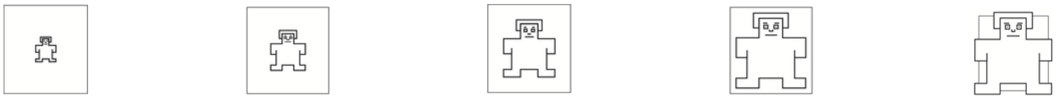

	1	2	3	4	5	6	7	8	9	
**No control**	○	○	○	○	○	○	○	○	○	**Completely in control**

Risk Assessment

After seeing this news post, how much anxiety do you feel in regards to the COVID-19 pandemic?*

	1	2	3	4	5	
**No anxiety at all**	○	○	○	○	○	**Very high anxiety**

After seeing the news post, how big of a risk do you think the COVID-19 pandemic poses to you or your family?*

	1	2	3	4	5	
**No risk at all**	○	○	○	○	○	**Very high risk**

Where 'dread' means to be in terror of, or fear intensely, how much do you dread the COVID-19 pandemic after seeing this news post?
*

	1	2	3	4	5	
**No dread at all**	○	○	○	○	○	**Very high dread**

After seeing the news post, what do you think is your likelihood of infection?*

	1	2	3	4	5	
**Very unlikely**	○	○	○	○	○	**Very likely**

After seeing the news post, how harmful do you think it would be if you got infected?*

	1	2	3	4	5	
**Not harmful at all**	○	○	○	○	○	**Very harmful**

Engagement

Are you likely to… (check where applicable)?*

- ☐ Share the post
- ☐ Read the post
- ☐ Comment on the post
- ☐ Ignore the post

Credibility

... can be trusted *

	1	2	3	4	5	
**Strongly disagree**	○	○	○	○	○	**Strongly agree**

...separates facts from opinion *

	1	2	3	4	5	
**Strongly disagree**	○	○	○	○	○	**Strongly agree**

...is factual *

	1	2	3	4	5	
**Strongly disagree**	○	○	○	○	○	**Strongly agree**

...tells the whole strory *

	1	2	3	4	5	
**Strongly disagree**	○	○	○	○	○	**Strongly agree**

...is accurate *

	1	2	3	4	5	
**Strongly disagree**	○	○	○	○	○	**Strongly agree**

...is unbiased *

	1	2	3	4	5	
**Strongly disagree**	○	○	○	○	○	**Strongly agree**

...is fair *

	1	2	3	4	5	
**Strongly disagree**	○	○	○	○	○	**Strongly agree**

... demonstrates concern about public interest *

	1	2	3	4	5	
**Strongly disagree**	○	○	○	○	○	**Strongly agree**

...is sensationalized *

	1	2	3	4	5	
**Strongly disagree**	○	○	○	○	○	**Strongly agree**

...is immoral *

	1	2	3	4	5	
**Strongly disagree**	○	○	○	○	○	**Strongly agree**

Image/Post

Please have a detailed look at the following image/post and tick 'I am done' when you finish:
*

☐ I am done

Attention test

For which part of the United Kingdom the recommendations/guidance were given in the last picture? *

○ England

○ Wales

○ Scotland

Feelings

Rate how positive or negative this post made you feel, ranging from **'sad'** to **'happy'**:

*

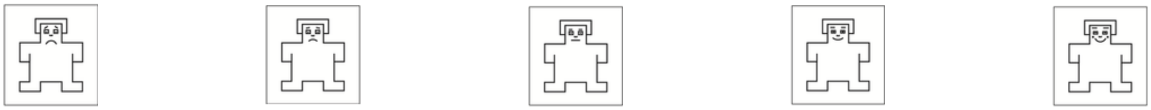

	1	2	3	4	5	6	7	8	9	
**Sad**	○	○	○	○	○	○	○	○	○	**Happy**

Rate how energetic this post made you feel, ranging from **'sleepy/apathetic'** to **'excited/energetic' :** *

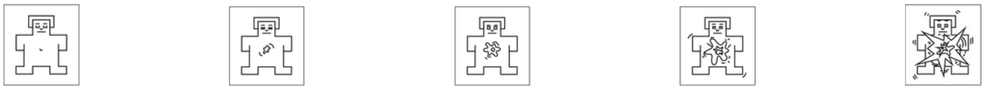

	1	2	3	4	5	6	7	8	9	
**Sleepy/Apathetic**	○	○	○	○	○	○	○	○	○	**Excited**

Rate how in control of the situation this post made you feel, ranging from **'no control'** to **'completely in control':** *

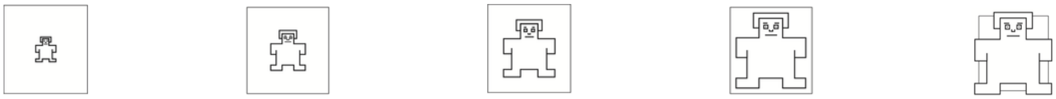

	1	2	3	4	5	6	7	8	9	
**No control**	○	○	○	○	○	○	○	○	○	**Completely in control**

Risk Assessment

After seeing this news post, how much anxiety do you feel in regards to the COVID-19 pandemic?*

	1	2	3	4	5	
**No anxiety at all**	○	○	○	○	○	**Very high anxiety**

After seeing the news post, how big of a risk do you think the COVID-19 pandemic poses to you or your family?*

	1	2	3	4	5	
**No risk at all**	○	○	○	○	○	**Very high risk**

Where ‘dread’ means to be in terror of, or fear intensely, how much do you dread the COVID-19 pandemic after seeing this news post?
*

	1	2	3	4	5	
**No dread at all**	○	○	○	○	○	**Very high dread**

After seeing the news post, what do you think is your likelihood of infection?*

	1	2	3	4	5	
**Very unlikely**	○	○	○	○	○	**Very likely**

After seeing the news post, how harmful do you think it would be if you got infected?*

	1	2	3	4	5	
**Not harmful at all**	○	○	○	○	○	**Very harmful**

Engagement

Are you likely to… (check where applicable)?*

- ☐ Share the post
- ☐ Read the post
- ☐ Comment on the post
- ☐ Ignore the post

Credibility

... can be trusted *

	1	2	3	4	5	
**Strongly disagree**	○	○	○	○	○	**Strongly agree**

...separates facts from opinion *

	1	2	3	4	5	
**Strongly disagree**	○	○	○	○	○	**Strongly agree**

...is factual *

	1	2	3	4	5	
**Strongly disagree**	○	○	○	○	○	**Strongly agree**

...tells the whole strory *

	1	2	3	4	5	
**Strongly disagree**	○	○	○	○	○	**Strongly agree**

...is accurate *

	1	2	3	4	5	
**Strongly disagree**	○	○	○	○	○	**Strongly agree**

...is unbiased *

	1	2	3	4	5	
**Strongly disagree**	○	○	○	○	○	**Strongly agree**

...is fair *

	1	2	3	4	5	
**Strongly disagree**	○	○	○	○	○	**Strongly agree**

... demonstrates concern about public interest *

	1	2	3	4	5	
**Strongly disagree**	○	○	○	○	○	**Strongly agree**

...is sensationalized *

	1	2	3	4	5	
**Strongly disagree**	○	○	○	○	○	**Strongly agree**

...is immoral *

	1	2	3	4	5	
**Strongly disagree**	○	○	○	○	○	**Strongly agree**

Image/Post

Please have a detailed look at the following image/post and tick 'I am done' when you finish:

*

☐ I am done

Attention test

What was the rightmost recommendation in the last picture?

○ Wash your hands

○ Keep your distance

○ Wear a face covering

Feelings

Rate how positive or negative this post made you feel, ranging from **'sad'** to **'happy'**:

*

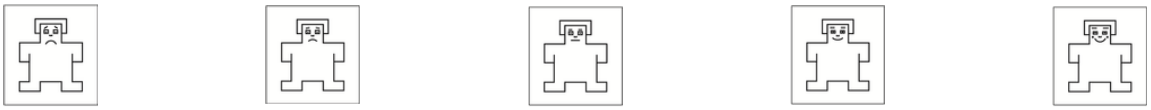

	1	2	3	4	5	6	7	8	9	
**Sad**	○	○	○	○	○	○	○	○	○	**Happy**

Rate how energetic this post made you feel, ranging from **'sleepy/apathetic'** to **'excited/energetic' :** *

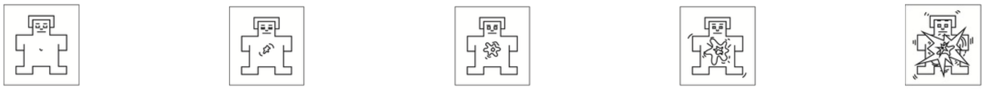

	1	2	3	4	5	6	7	8	9	
**Sleepy/Apathetic**	○	○	○	○	○	○	○	○	○	**Excited**

Rate how in control of the situation this post made you feel, ranging from **'no control'** to **'completely in control':** *

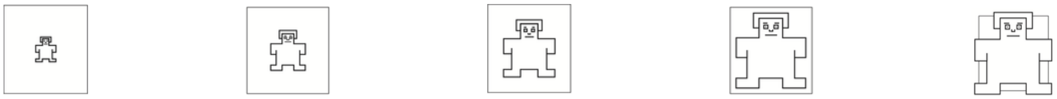

	1	2	3	4	5	6	7	8	9	
**No control**	○	○	○	○	○	○	○	○	○	**Completely in control**

Risk Assessment

After seeing this news post, how much anxiety do you feel in regards to the COVID-19 pandemic?*

	1	2	3	4	5	
**No anxiety at all**	○	○	○	○	○	**Very high anxiety**

After seeing the news post, how big of a risk do you think the COVID-19 pandemic poses to you or your family?*

	1	2	3	4	5	
**No risk at all**	○	○	○	○	○	**Very high risk**

Where 'dread' means to be in terror of, or fear intensely, how much do you dread the COVID-19 pandemic after seeing this news post?
*

	1	2	3	4	5	
**No dread at all**	○	○	○	○	○	**Very high dread**

After seeing the news post, what do you think is your likelihood of infection?*

	1	2	3	4	5	
**Very unlikely**	○	○	○	○	○	**Very likely**

After seeing the news post, how harmful do you think it would be if you got infected?*

	1	2	3	4	5	
**Not harmful at all**	○	○	○	○	○	**Very harmful**

Engagement

Are you likely to… (check where applicable)?*

- ☐ Share the post
- ☐ Read the post
- ☐ Comment on the post
- ☐ Ignore the post

Credibility

... can be trusted *

	1	2	3	4	5	
**Strongly disagree**	○	○	○	○	○	**Strongly agree**

...separates facts from opinion *

	1	2	3	4	5	
**Strongly disagree**	○	○	○	○	○	**Strongly agree**

...is factual *

	1	2	3	4	5	
**Strongly disagree**	○	○	○	○	○	**Strongly agree**

...tells the whole strory *

	1	2	3	4	5	
**Strongly disagree**	○	○	○	○	○	**Strongly agree**

...is accurate *

	1	2	3	4	5	
**Strongly disagree**	○	○	○	○	○	**Strongly agree**

...is unbiased *

	1	2	3	4	5	
**Strongly disagree**	○	○	○	○	○	**Strongly agree**

...is fair *

	1	2	3	4	5	
**Strongly disagree**	○	○	○	○	○	**Strongly agree**

... demonstrates concern about public interest *

	1	2	3	4	5	
**Strongly disagree**	○	○	○	○	○	**Strongly agree**

...is sensationalized *

	1	2	3	4	5	
**Strongly disagree**	○	○	○	○	○	**Strongly agree**

...is immoral *

	1	2	3	4	5	
**Strongly disagree**	○	○	○	○	○	**Strongly agree**

Image/Post

Please have a detailed look at the following image/post and tick 'I am done' when you finish:
*

- [ ] I am done

Attention test

Which months were represented by the bar chart in the last picture? *

- February and April
- October and December
- January and September

Feelings

Rate how positive or negative this post made you feel, ranging from **'sad'** to **'happy'**:

*

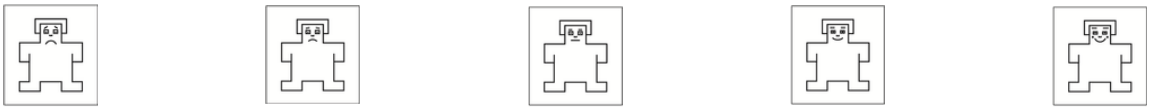

	1	2	3	4	5	6	7	8	9	
**Sad**	○	○	○	○	○	○	○	○	○	**Happy**

Rate how energetic this post made you feel, ranging from **'sleepy/apathetic'** to **'excited/energetic' :** *

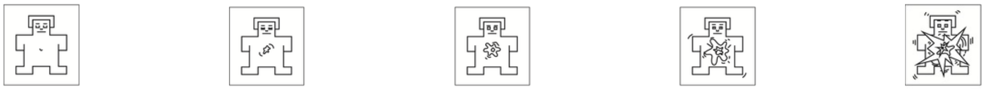

	1	2	3	4	5	6	7	8	9	
**Sleepy/Apathetic**	○	○	○	○	○	○	○	○	○	**Excited**

Rate how in control of the situation this post made you feel, ranging from **'no control'** to **'completely in control':** *

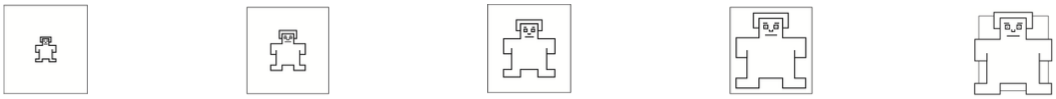

	1	2	3	4	5	6	7	8	9	
**No control**	○	○	○	○	○	○	○	○	○	**Completely in control**

Risk Assessment

After seeing this news post, how much anxiety do you feel in regards to the COVID-19 pandemic?*

	1	2	3	4	5	
**No anxiety at all**	○	○	○	○	○	**Very high anxiety**

After seeing the news post, how big of a risk do you think the COVID-19 pandemic poses to you or your family?*

	1	2	3	4	5	
**No risk at all**	○	○	○	○	○	**Very high risk**

Where 'dread' means to be in terror of, or fear intensely, how much do you dread the COVID-19 pandemic after seeing this news post?
*

	1	2	3	4	5	
**No dread at all**	○	○	○	○	○	**Very high dread**

After seeing the news post, what do you think is your likelihood of infection?*

	1	2	3	4	5	
**Very unlikely**	○	○	○	○	○	**Very likely**

After seeing the news post, how harmful do you think it would be if you got infected?*

	1	2	3	4	5	
**Not harmful at all**	○	○	○	○	○	**Very harmful**

Engagement

Are you likely to… (check where applicable)? *

- ☐ Share the post
- ☐ Read the post
- ☐ Comment on the post
- ☐ Ignore the post

Credibility

... can be trusted *

	1	2	3	4	5	
**Strongly disagree**	○	○	○	○	○	**Strongly agree**

...separates facts from opinion *

	1	2	3	4	5	
**Strongly disagree**	○	○	○	○	○	**Strongly agree**

...is factual *

	1	2	3	4	5	
**Strongly disagree**	○	○	○	○	○	**Strongly agree**

...tells the whole strory *

	1	2	3	4	5	
**Strongly disagree**	○	○	○	○	○	**Strongly agree**

...is accurate *

	1	2	3	4	5	
**Strongly disagree**	○	○	○	○	○	**Strongly agree**

...is unbiased *

	1	2	3	4	5	
**Strongly disagree**	◯	◯	◯	◯	◯	**Strongly agree**

...is fair *

	1	2	3	4	5	
**Strongly disagree**	◯	◯	◯	◯	◯	**Strongly agree**

... demonstrates concern about public interest *

	1	2	3	4	5	
**Strongly disagree**	◯	◯	◯	◯	◯	**Strongly agree**

...is sensationalized *

	1	2	3	4	5	
**Strongly disagree**	◯	◯	◯	◯	◯	**Strongly agree**

...is immoral *

	1	2	3	4	5	
**Strongly disagree**	◯	◯	◯	◯	◯	**Strongly agree**

Image/Post

Please have a detailed look at the following image/post and tick 'I am done' when you finish:
*

☐ I am done

Attention test

Which country was referred to by the chart in the last picture? *

- UK
- US
- Australia

Feelings

Rate how positive or negative this post made you feel, ranging from **'sad'** to **'happy'**:

*

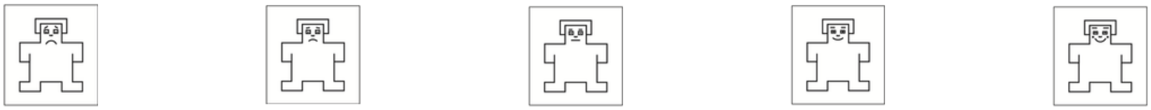

	1	2	3	4	5	6	7	8	9	
**Sad**	○	○	○	○	○	○	○	○	○	**Happy**

Rate how energetic this post made you feel, ranging from **'sleepy/apathetic'** to **'excited/energetic' :** *

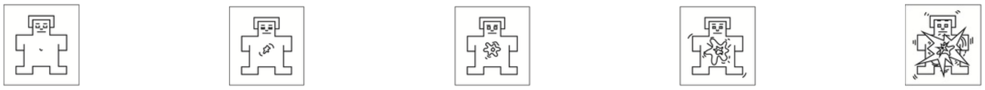

	1	2	3	4	5	6	7	8	9	
**Sleepy/Apathetic**	○	○	○	○	○	○	○	○	○	**Excited**

Rate how in control of the situation this post made you feel, ranging from **'no control'** to **'completely in control':** *

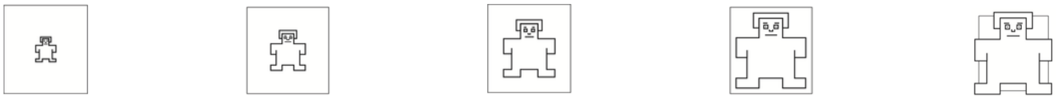

	1	2	3	4	5	6	7	8	9	
**No control**	○	○	○	○	○	○	○	○	○	**Completely in control**

Risk Assessment

After seeing this news post, how much anxiety do you feel in regards to the COVID-19 pandemic?*

	1	2	3	4	5	
**No anxiety at all**	○	○	○	○	○	**Very high anxiety**

After seeing the news post, how big of a risk do you think the COVID-19 pandemic poses to you or your family?*

	1	2	3	4	5	
**No risk at all**	○	○	○	○	○	**Very high risk**

Where ‘dread’ means to be in terror of, or fear intensely, how much do you dread the COVID-19 pandemic after seeing this news post?
*

	1	2	3	4	5	
**No dread at all**	○	○	○	○	○	**Very high dread**

After seeing the news post, what do you think is your likelihood of infection?*

	1	2	3	4	5	
**Very unlikely**	○	○	○	○	○	**Very likely**

After seeing the news post, how harmful do you think it would be if you got infected?*

	1	2	3	4	5	
**Not harmful at all**	○	○	○	○	○	**Very harmful**

Engagement

Are you likely to… (check where applicable)?*

- ☐ Share the post
- ☐ Read the post
- ☐ Comment on the post
- ☐ Ignore the post

Credibility

... can be trusted *

	1	2	3	4	5	
**Strongly disagree**	○	○	○	○	○	**Strongly agree**

...separates facts from opinion *

	1	2	3	4	5	
**Strongly disagree**	○	○	○	○	○	**Strongly agree**

...is factual *

	1	2	3	4	5	
**Strongly disagree**	○	○	○	○	○	**Strongly agree**

...tells the whole strory *

	1	2	3	4	5	
**Strongly disagree**	○	○	○	○	○	**Strongly agree**

...is accurate *

	1	2	3	4	5	
**Strongly disagree**	○	○	○	○	○	**Strongly agree**

...is unbiased *

	1	2	3	4	5	
**Strongly disagree**	○	○	○	○	○	**Strongly agree**

...is fair *

	1	2	3	4	5	
**Strongly disagree**	○	○	○	○	○	**Strongly agree**

... demonstrates concern about public interest *

	1	2	3	4	5	
**Strongly disagree**	○	○	○	○	○	**Strongly agree**

...is sensationalized *

	1	2	3	4	5	
**Strongly disagree**	○	○	○	○	○	**Strongly agree**

...is immoral *

	1	2	3	4	5	
**Strongly disagree**	○	○	○	○	○	**Strongly agree**

Image/Post

Please have a detailed look at the following image/post and tick 'I am done' when you finish:
*

- [ ] I am done

Attention test

What was represented on the left and right sides of the chart in the last picture? *

- ○ Boys and Girls
- ○ Males and Females
- ○ Educated and Uneducated

Feelings

Rate how positive or negative this post made you feel, ranging from **'sad'** to **'happy'**:

*

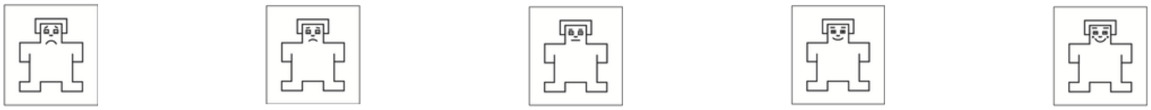

	1	2	3	4	5	6	7	8	9	
**Sad**	○	○	○	○	○	○	○	○	○	**Happy**

Rate how energetic this post made you feel, ranging from **'sleepy/apathetic'** to **'excited/energetic' :** *

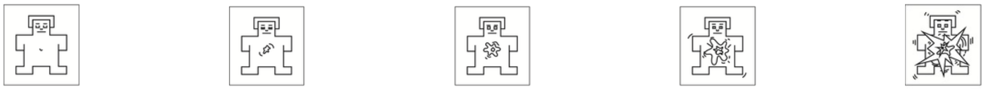

	1	2	3	4	5	6	7	8	9	
**Sleepy/Apathetic**	○	○	○	○	○	○	○	○	○	**Excited**

Rate how in control of the situation this post made you feel, ranging from **'no control'** to **'completely in control':** *

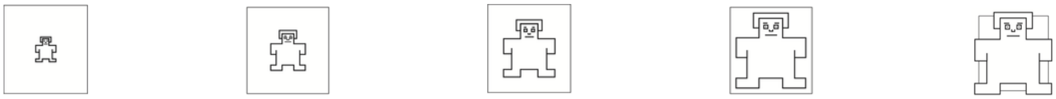

	1	2	3	4	5	6	7	8	9	
**No control**	○	○	○	○	○	○	○	○	○	**Completely in control**

Risk Assessment

After seeing this news post, how much anxiety do you feel in regards to the COVID-19 pandemic?*

	1	2	3	4	5	
**No anxiety at all**	○	○	○	○	○	**Very high anxiety**

After seeing the news post, how big of a risk do you think the COVID-19 pandemic poses to you or your family?*

	1	2	3	4	5	
**No risk at all**	○	○	○	○	○	**Very high risk**

Where ‘dread’ means to be in terror of, or fear intensely, how much do you dread the COVID-19 pandemic after seeing this news post?
*

	1	2	3	4	5	
**No dread at all**	○	○	○	○	○	**Very high dread**

After seeing the news post, what do you think is your likelihood of infection?*

	1	2	3	4	5	
**Very unlikely**	○	○	○	○	○	**Very likely**

After seeing the news post, how harmful do you think it would be if you got infected?*

	1	2	3	4	5	
**Not harmful at all**	○	○	○	○	○	**Very harmful**

Engagement

Are you likely to… (check where applicable)?*

- ☐ Share the post
- ☐ Read the post
- ☐ Comment on the post
- ☐ Ignore the post

Credibility

... can be trusted *

	1	2	3	4	5	
**Strongly disagree**	○	○	○	○	○	**Strongly agree**

...separates facts from opinion *

	1	2	3	4	5	
**Strongly disagree**	○	○	○	○	○	**Strongly agree**

...is factual *

	1	2	3	4	5	
**Strongly disagree**	○	○	○	○	○	**Strongly agree**

...tells the whole strory *

	1	2	3	4	5	
**Strongly disagree**	○	○	○	○	○	**Strongly agree**

...is accurate *

	1	2	3	4	5	
**Strongly disagree**	○	○	○	○	○	**Strongly agree**

...is unbiased *

	1	2	3	4	5	
**Strongly disagree**	○	○	○	○	○	**Strongly agree**

...is fair *

	1	2	3	4	5	
**Strongly disagree**	○	○	○	○	○	**Strongly agree**

... demonstrates concern about public interest *

	1	2	3	4	5	
**Strongly disagree**	○	○	○	○	○	**Strongly agree**

...is sensationalized *

	1	2	3	4	5	
**Strongly disagree**	○	○	○	○	○	**Strongly agree**

...is immoral *

	1	2	3	4	5	
**Strongly disagree**	○	○	○	○	○	**Strongly agree**

Demographic Information and Internet Usage Habits

Your Age: *

Your Gender: *

- ○ Male
- ○ Female

What is the highest degree or education level you have completed: *

- ○ High School
- ○ Bachelor's degree
- ○ Master's degree
- ○ Ph.D or higher
- ○ Prefer not to say
- ○ None

Which of the following social media do you use? *

- ☐ Facebook
- ☐ Instagram
- ☐ Twitter
- ☐ YouTube
- ☐ LinkedIn
- ☐ Other - Write In (Required)

__________ *

How many hours per day do you spend on social media? *

____

What is your main source of news about the world? *

- ○ Television
- ○ News websites
- ○ Social media
- ○ Newspaper
- ○ Podcasts
- ○ Radio
- ○ Other - Write In (Required)

____

How active are you on social media through commenting, sharing, liking or posting content? *

	1	2	3	4	5	
**Not active at all**	○	○	○	○	○	**Extremely active**

Thank You!

Thank you for taking our survey. Your response is very important to us.

Here is your completion code: [question('value'), id='301']
