# Supplementary figures and images for: Effect of visual imagery in COVID-19 social media posts on users’ perception
Source: PeerJ Comput Sci. 2022 Nov 15;8:e1153. doi: 10.7717/peerj-cs.1153 (PMC9680878; doi:10.7717/peerj-cs.1153)

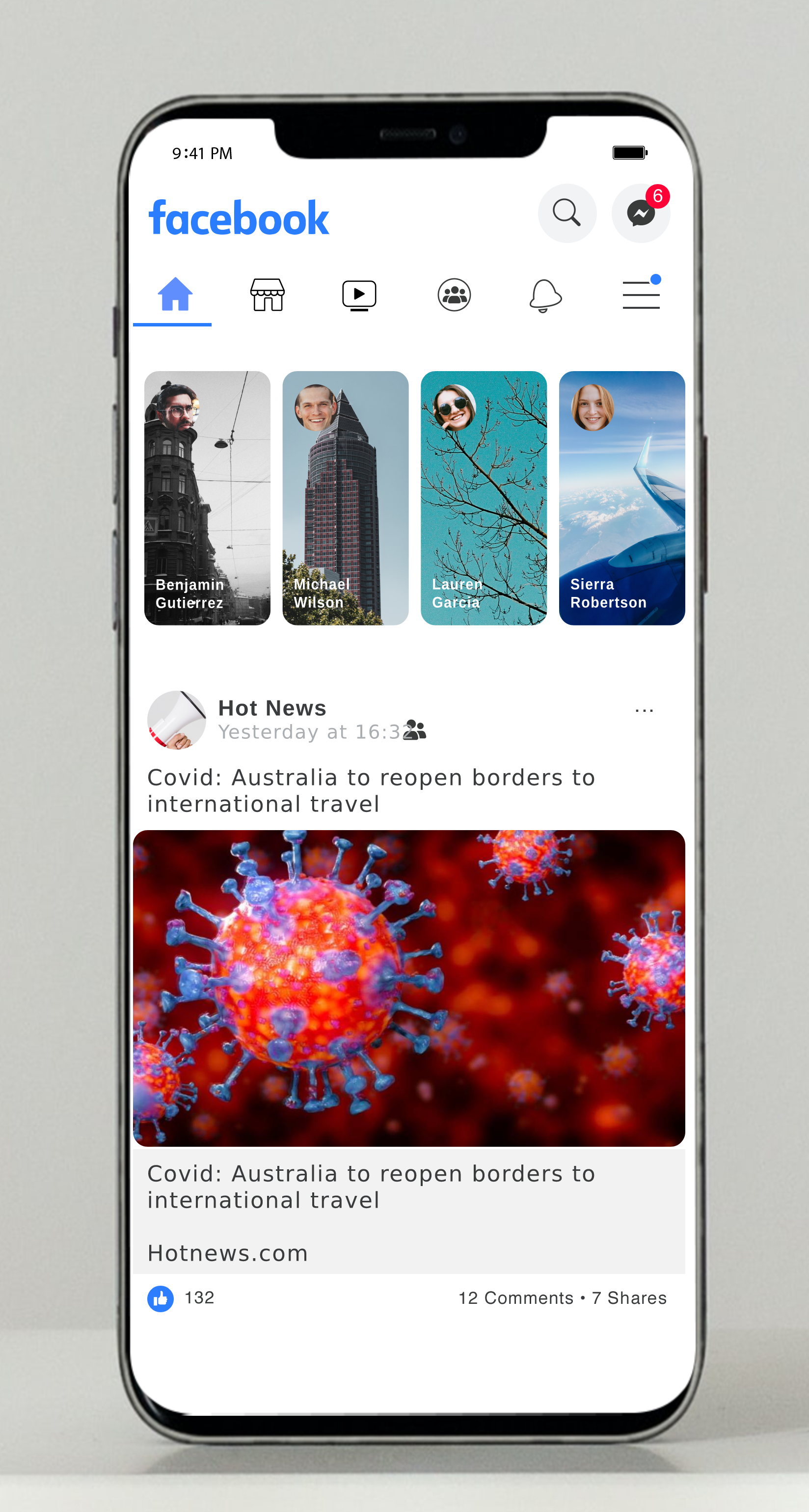

Supplement: Supplemental Information 4 [file peerj-cs-08-1153-s004.zip › PS1_Survey+Stimuli/H1_B1.png]

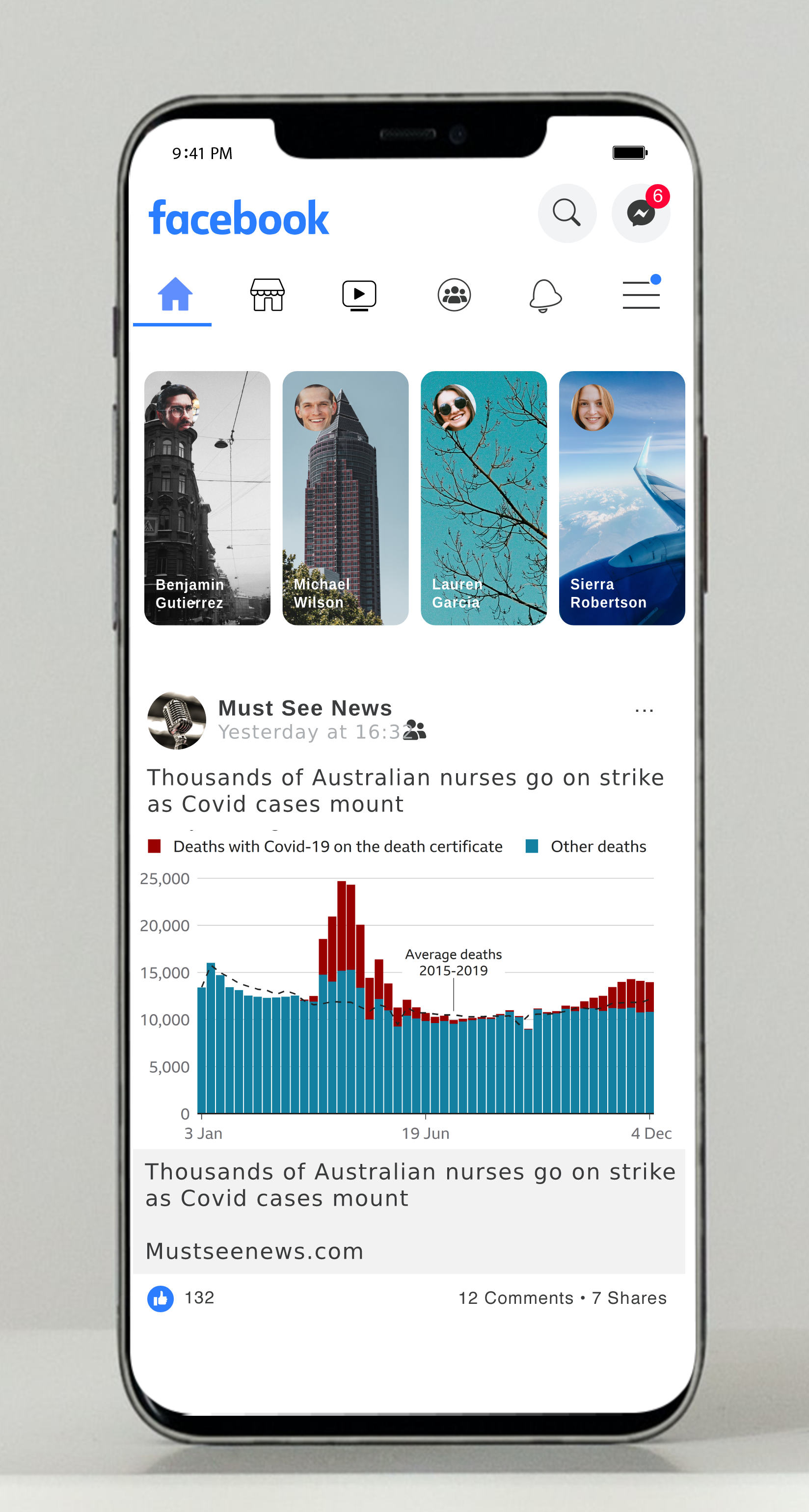

Supplement: Supplemental Information 4 [file peerj-cs-08-1153-s004.zip › PS1_Survey+Stimuli/H10_V2.png]

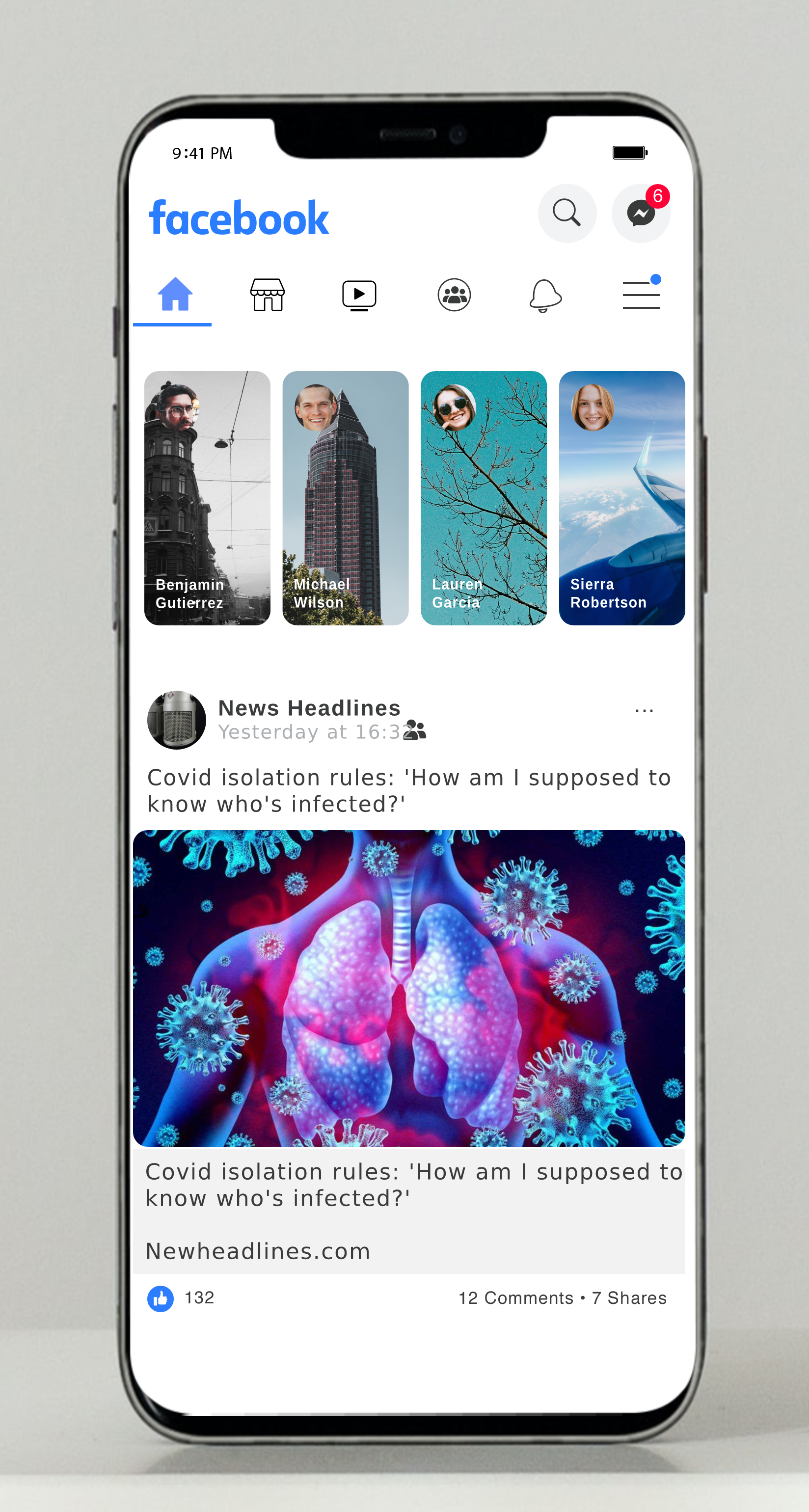

Supplement: Supplemental Information 4 [file peerj-cs-08-1153-s004.zip › PS1_Survey+Stimuli/H11_B2.png]

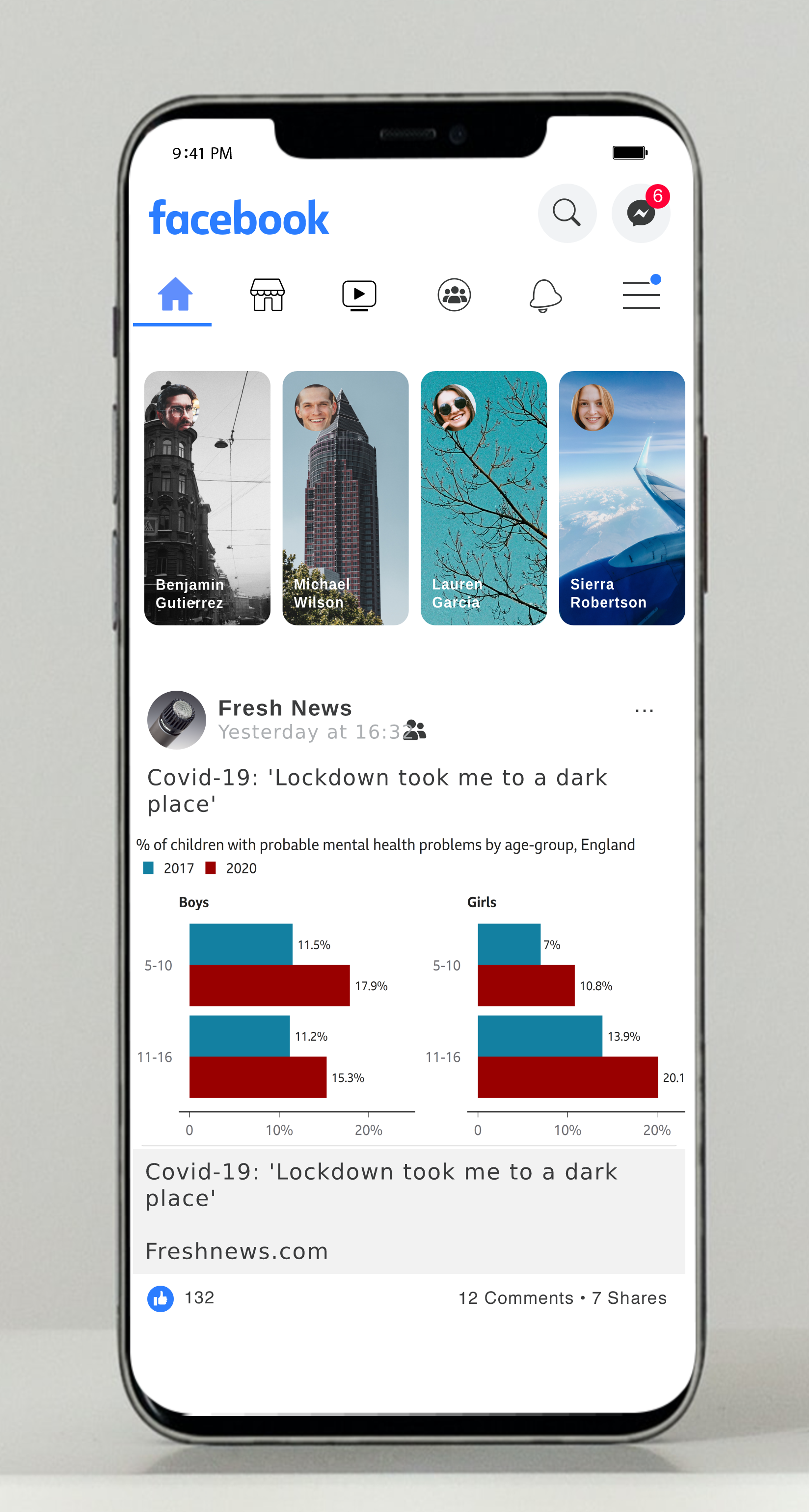

Supplement: Supplemental Information 4 [file peerj-cs-08-1153-s004.zip › PS1_Survey+Stimuli/H3_V4.png]

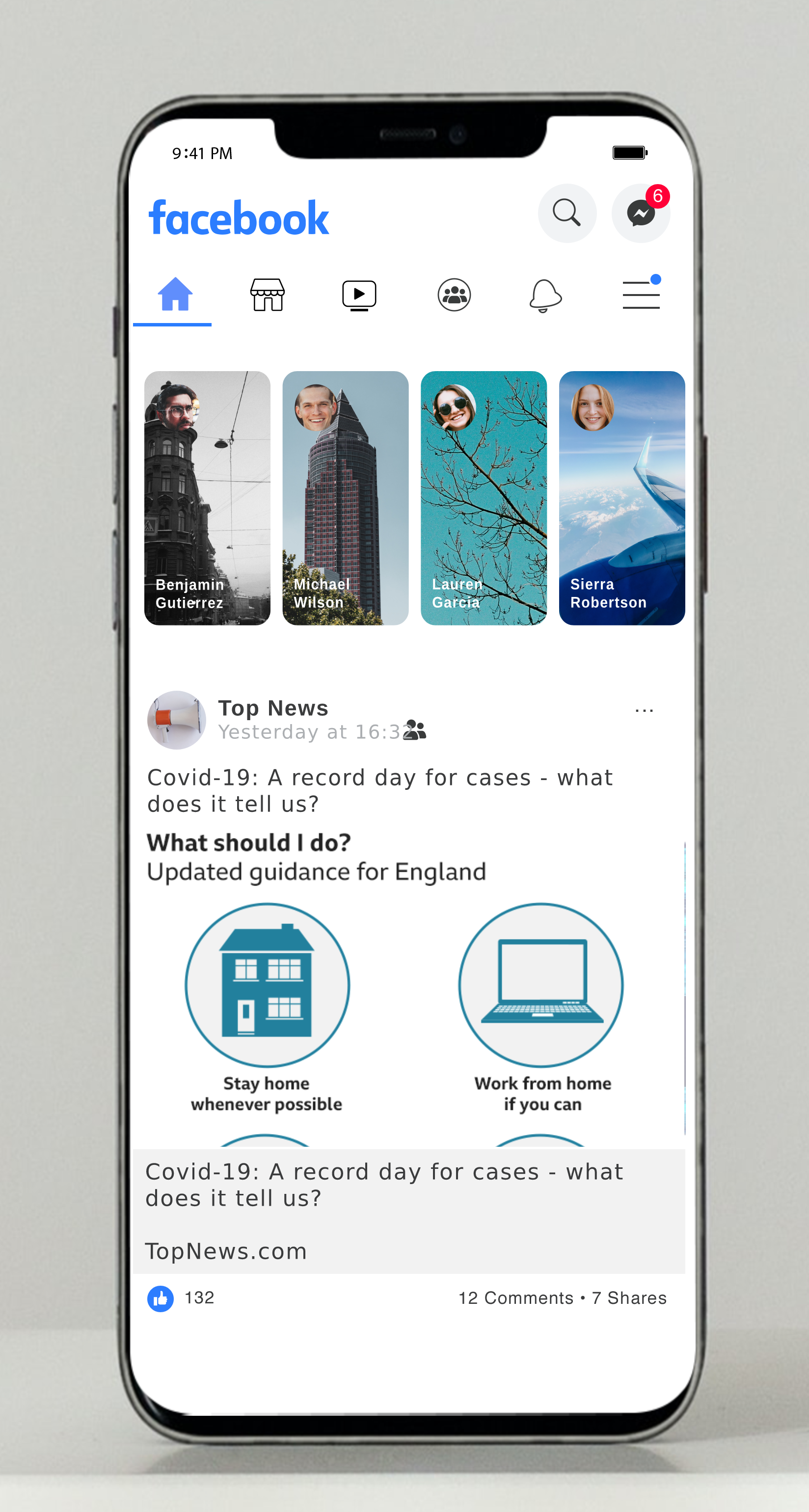

Supplement: Supplemental Information 4 [file peerj-cs-08-1153-s004.zip › PS1_Survey+Stimuli/H5_A2.png]

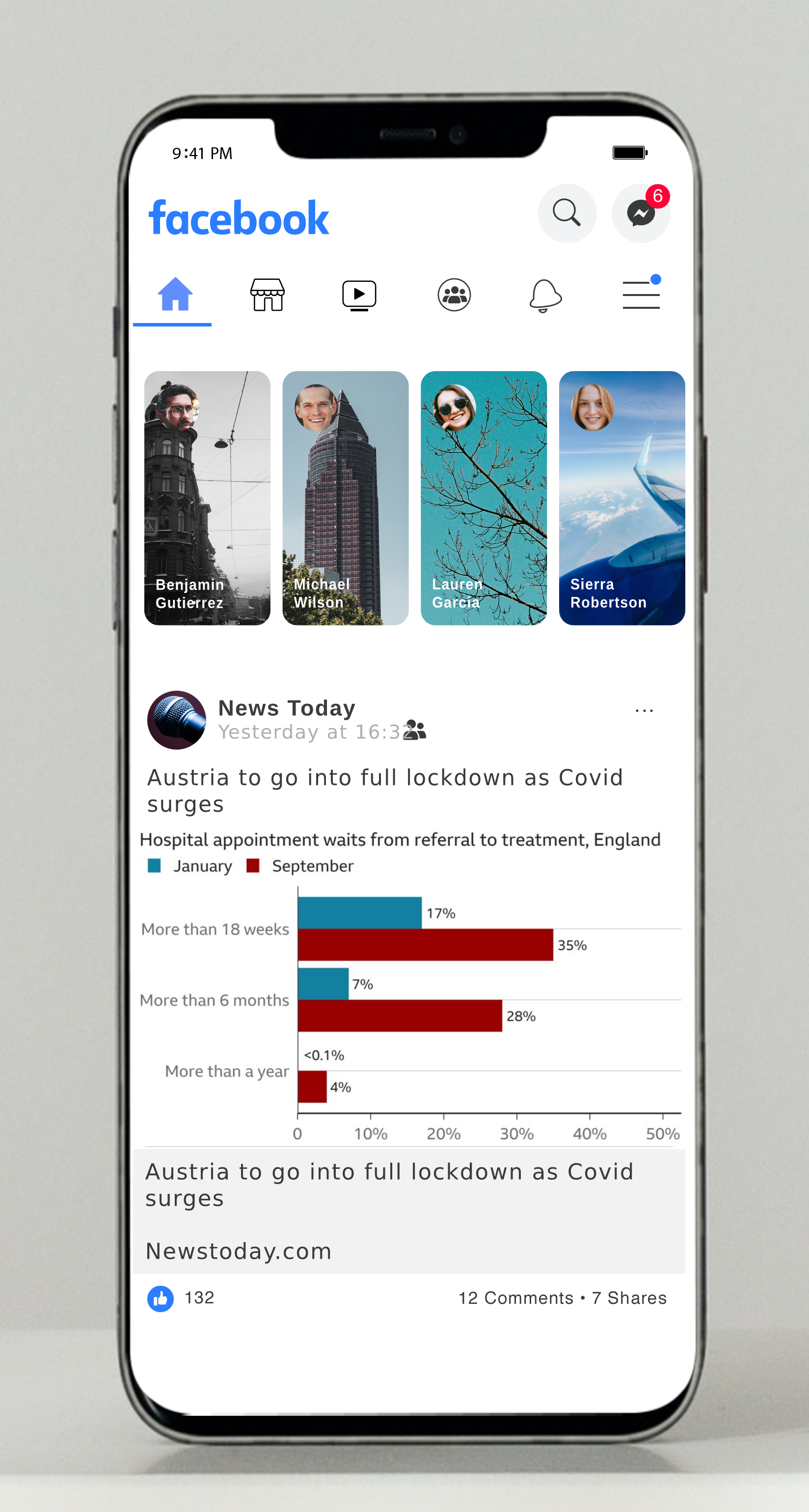

Supplement: Supplemental Information 4 [file peerj-cs-08-1153-s004.zip › PS1_Survey+Stimuli/H6_V1.png]

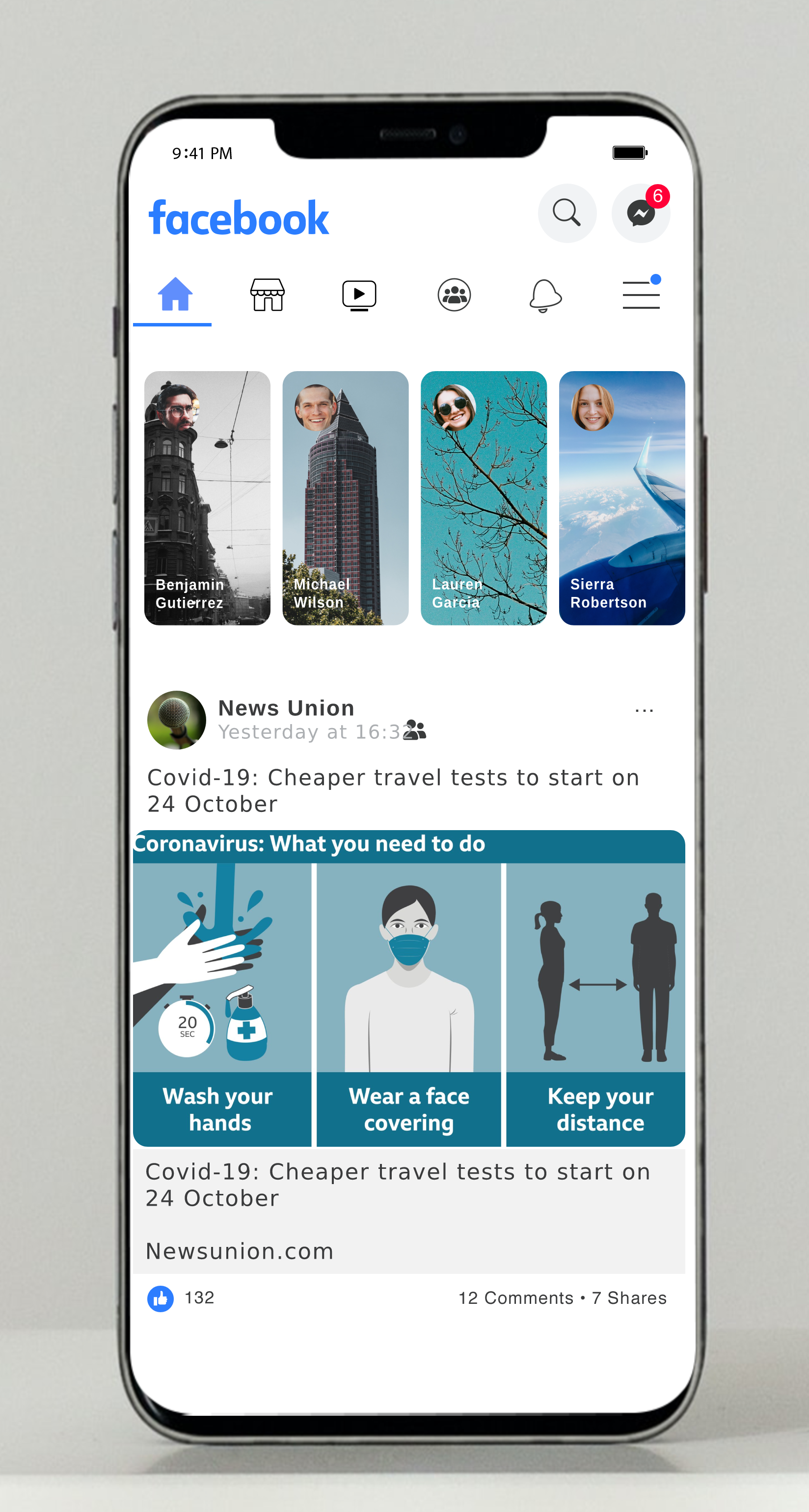

Supplement: Supplemental Information 4 [file peerj-cs-08-1153-s004.zip › PS1_Survey+Stimuli/H7_A4.png]

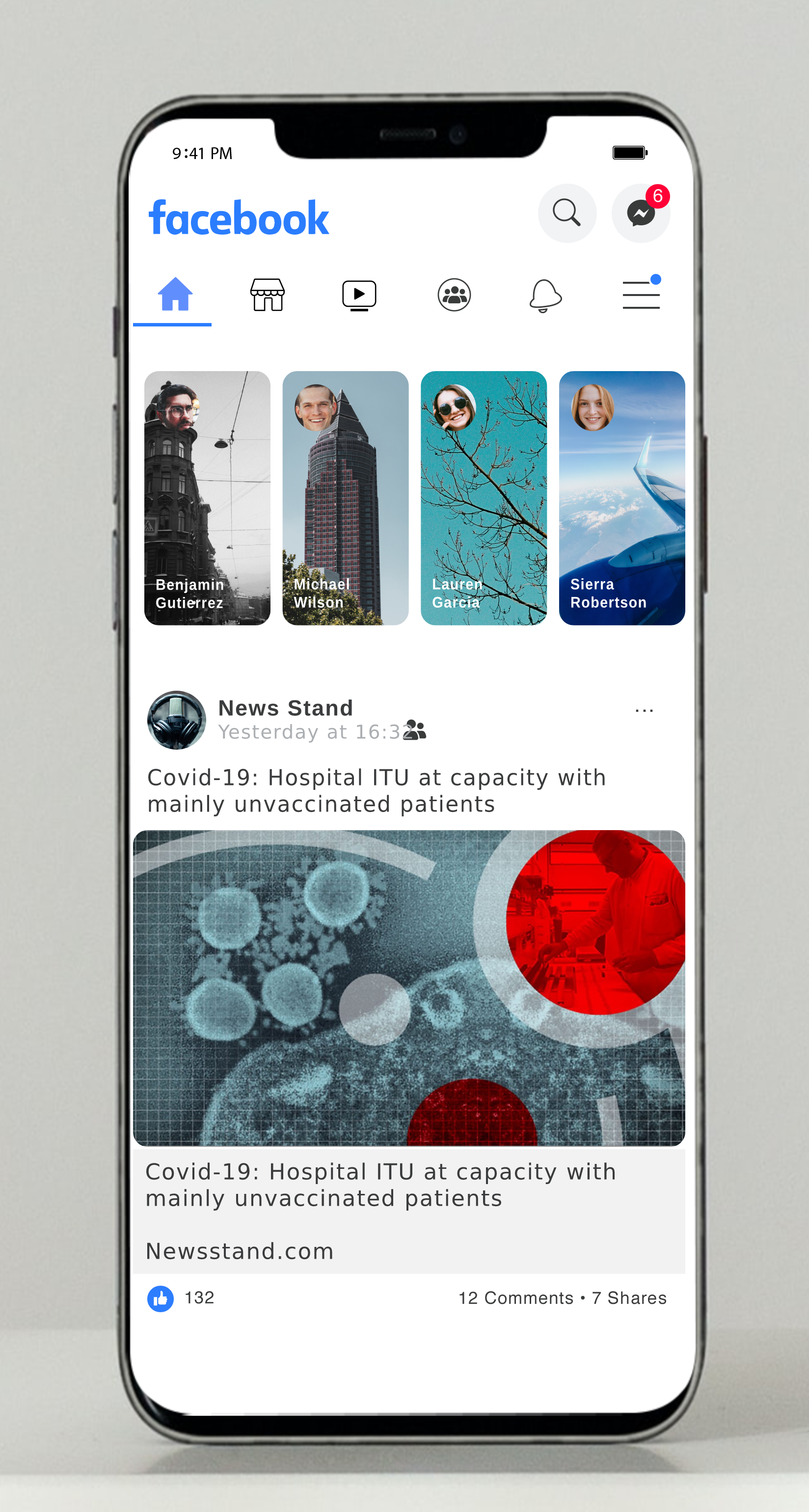

Supplement: Supplemental Information 4 [file peerj-cs-08-1153-s004.zip › PS1_Survey+Stimuli/H8_B4.png]

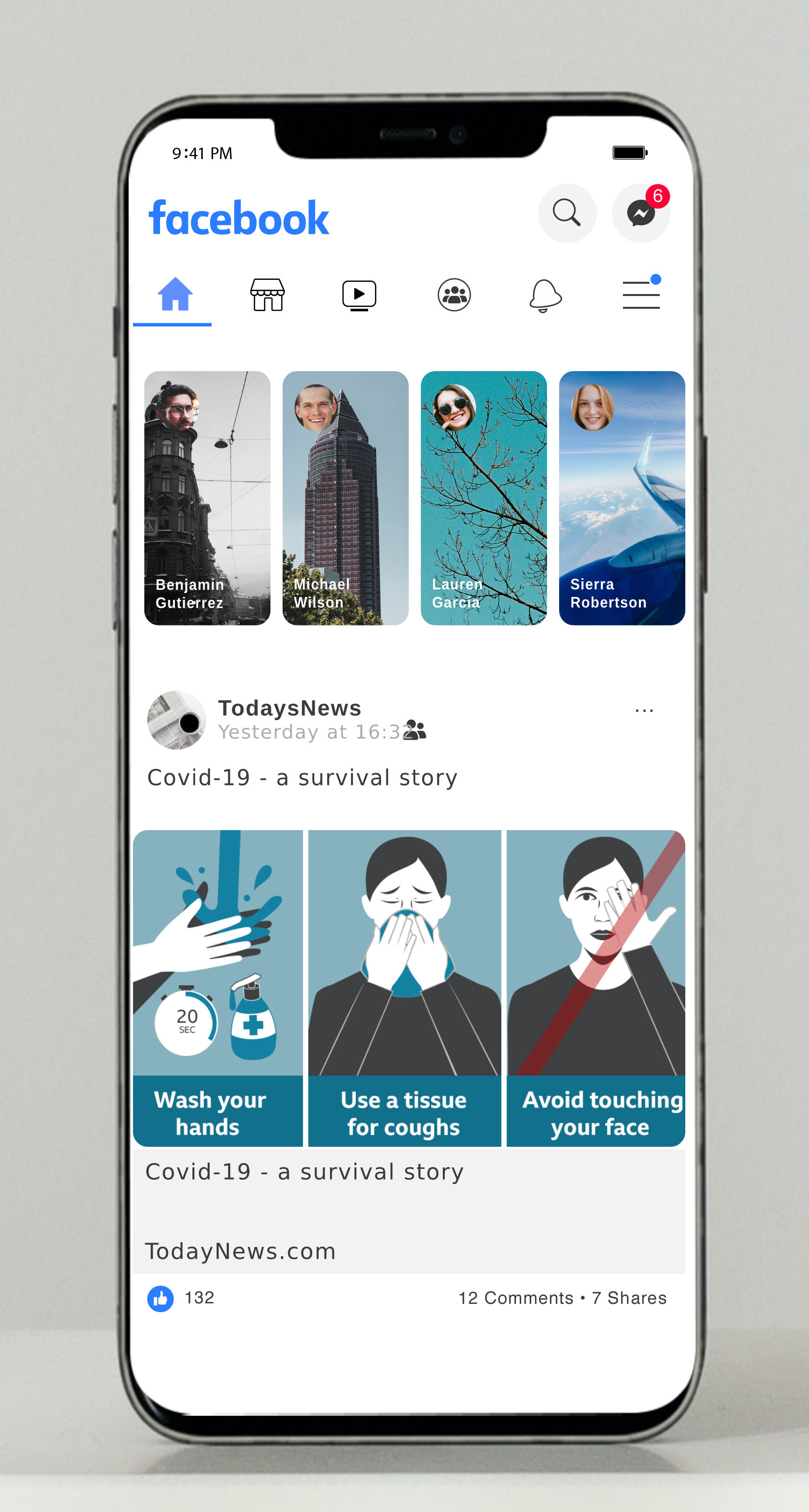

Supplement: Supplemental Information 4 [file peerj-cs-08-1153-s004.zip › PS1_Survey+Stimuli/H9_A1.png]

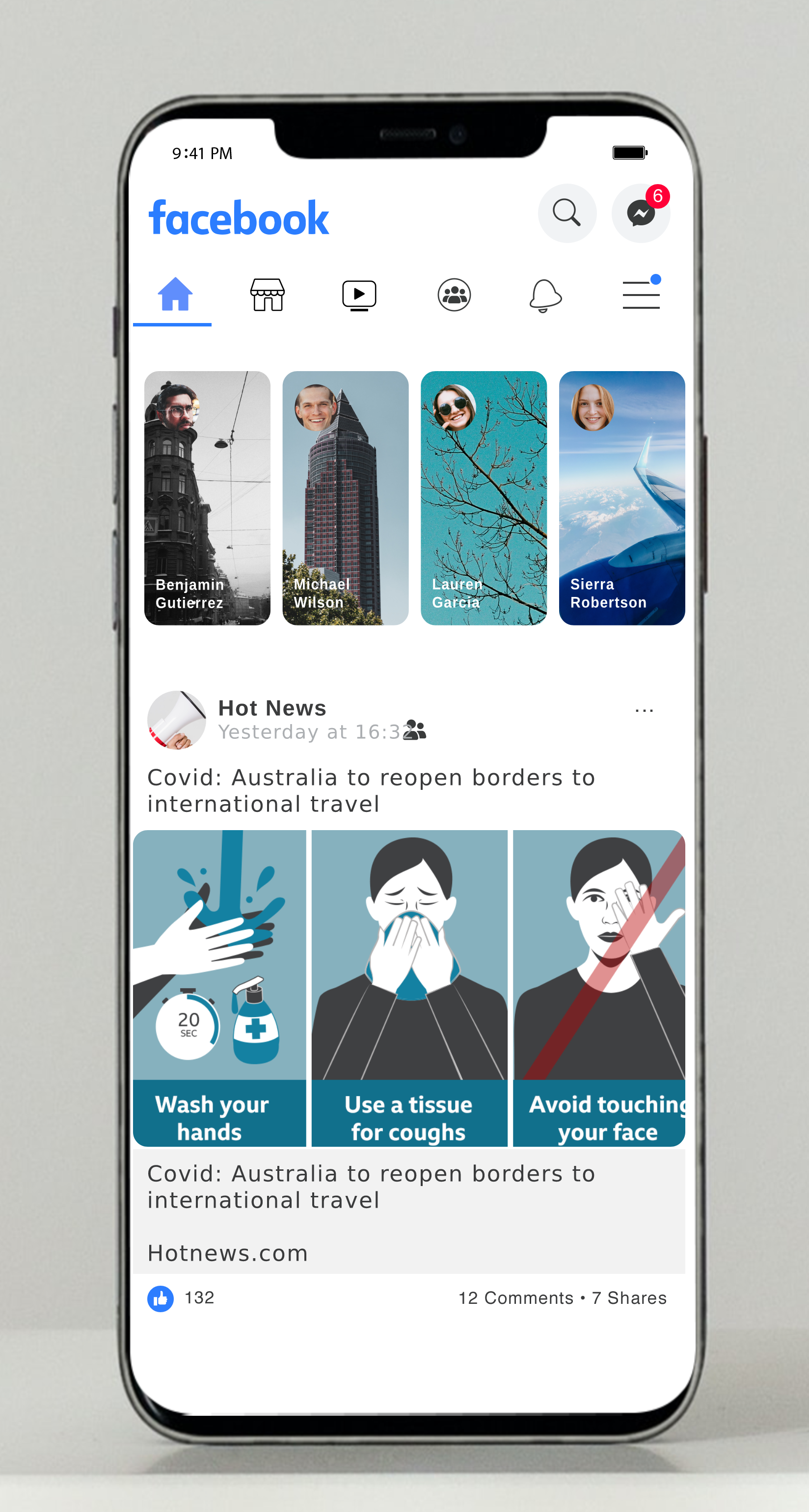

Supplement: Supplemental Information 5 [file peerj-cs-08-1153-s005.zip › PS2_Survey+stimuli/H1_A1.png]

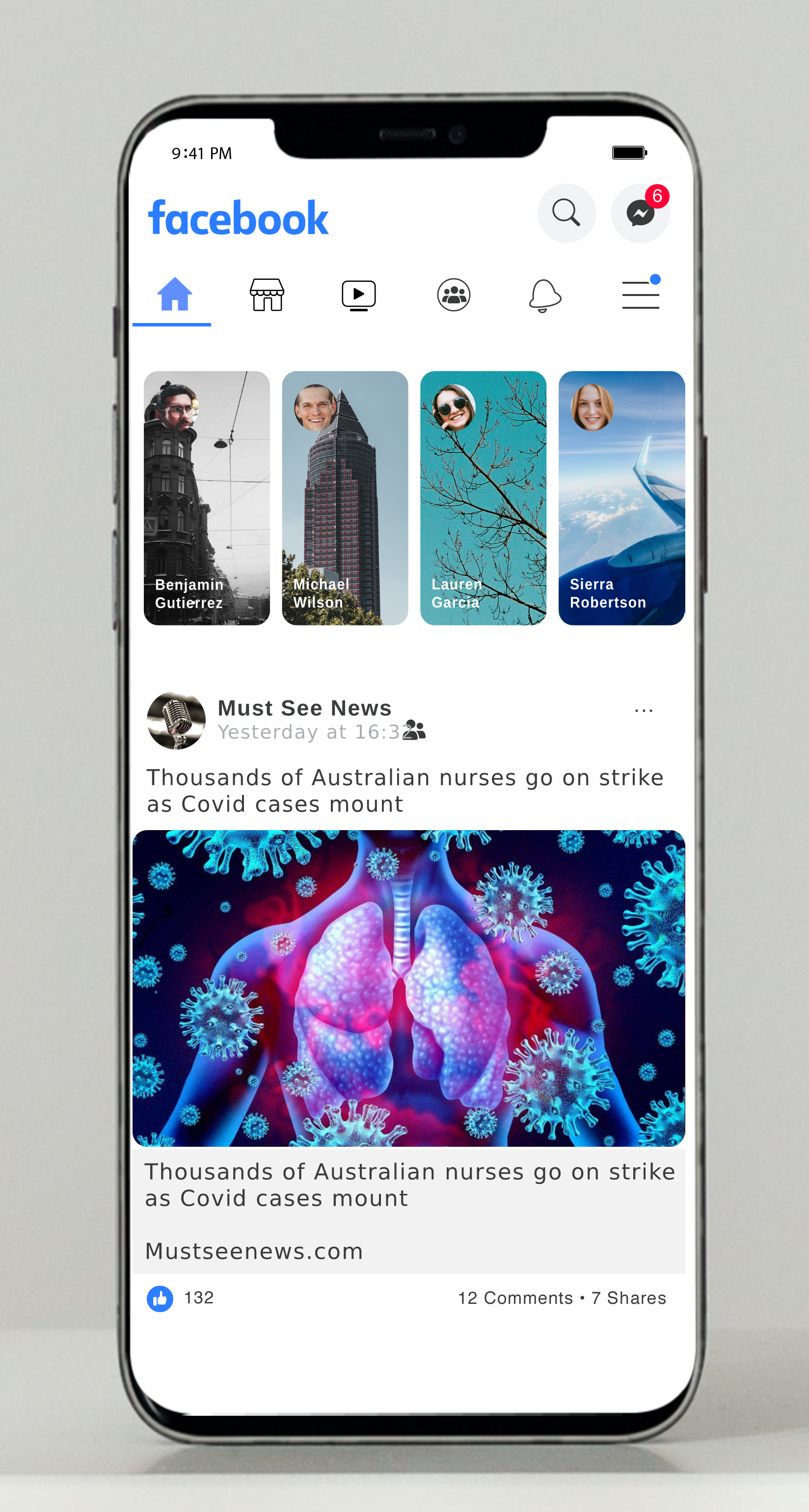

Supplement: Supplemental Information 5 [file peerj-cs-08-1153-s005.zip › PS2_Survey+stimuli/H10_B2.png]

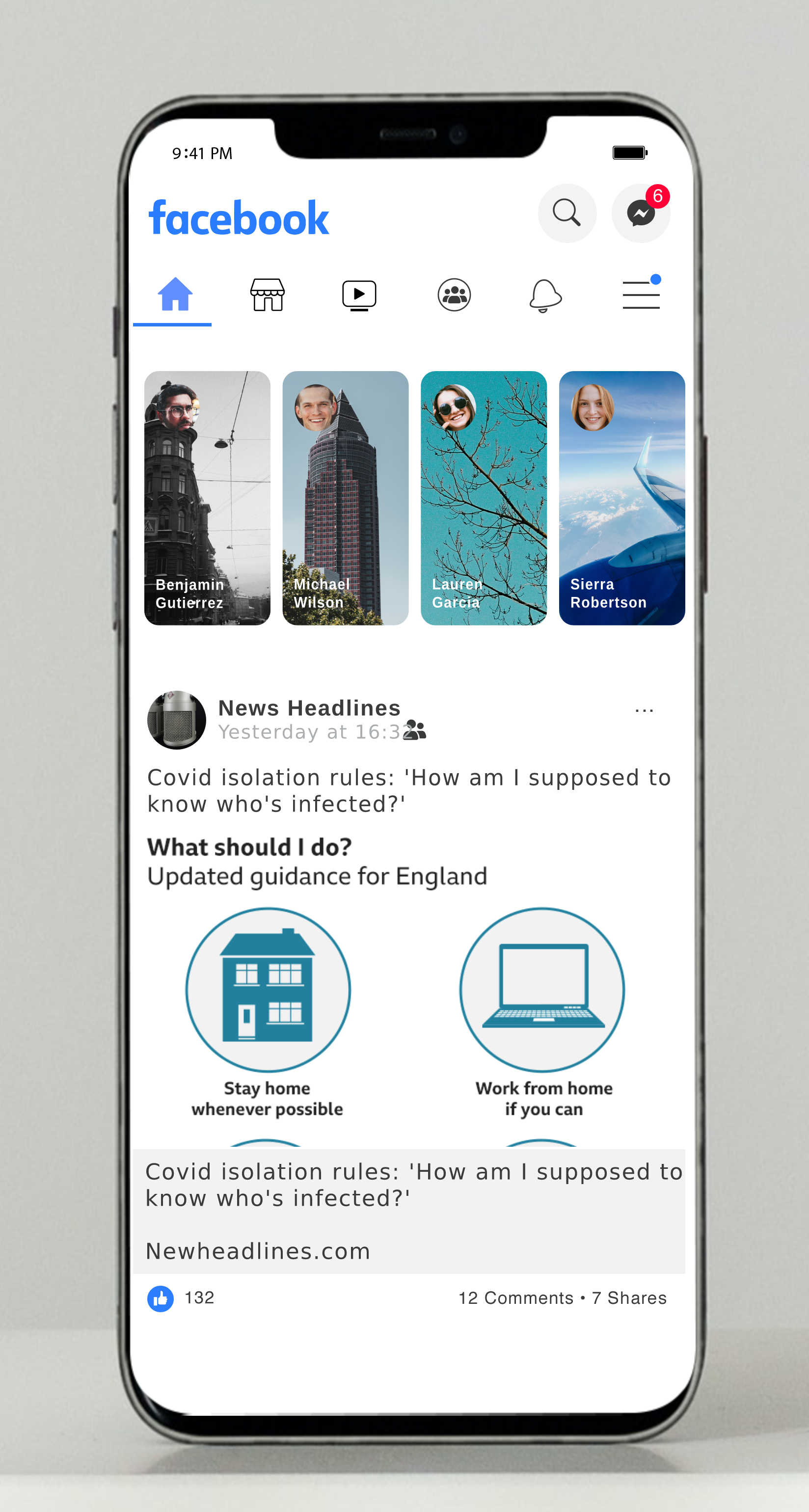

Supplement: Supplemental Information 5 [file peerj-cs-08-1153-s005.zip › PS2_Survey+stimuli/H11_A2.png]

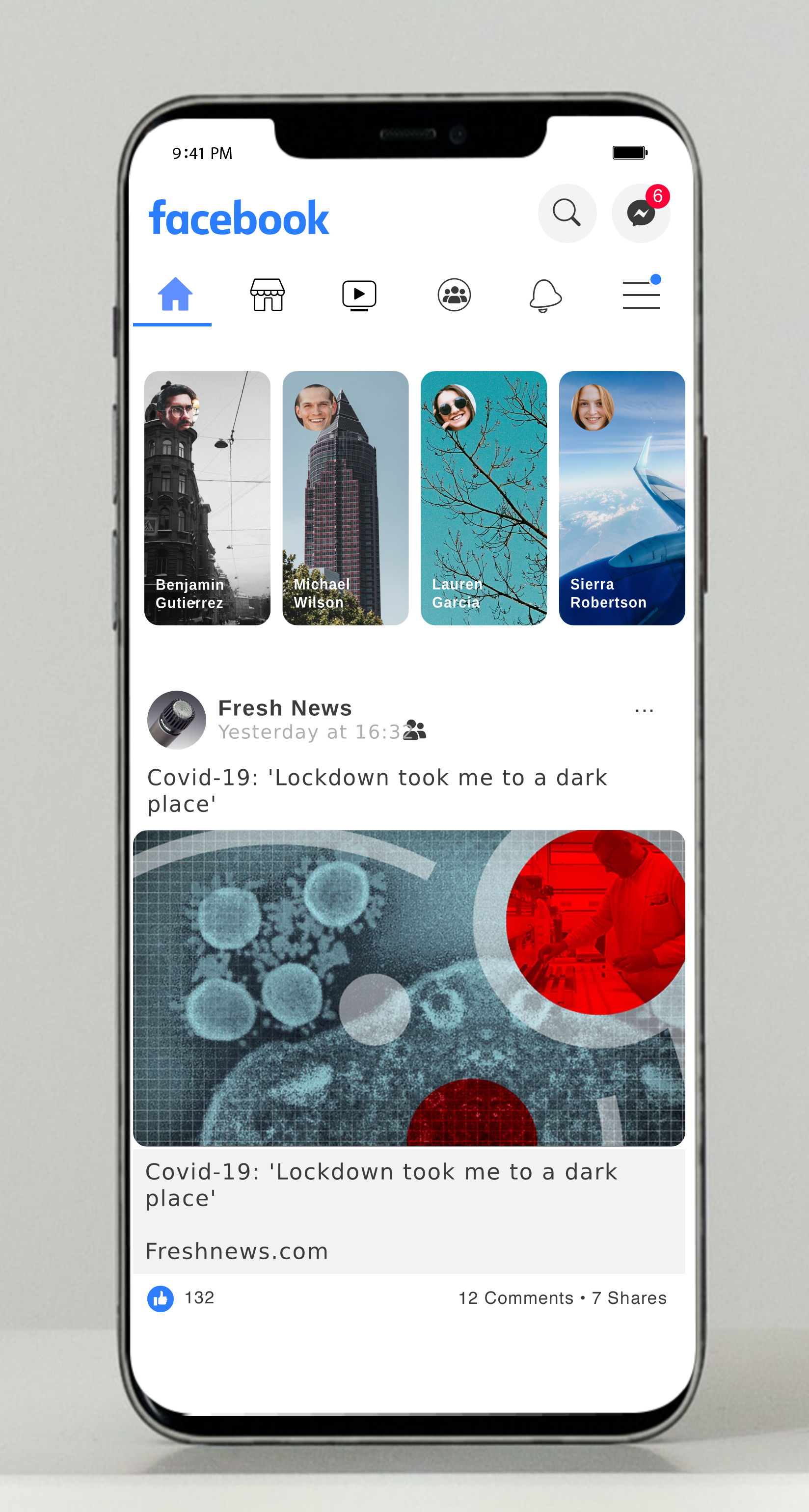

Supplement: Supplemental Information 5 [file peerj-cs-08-1153-s005.zip › PS2_Survey+stimuli/H3_B4.png]

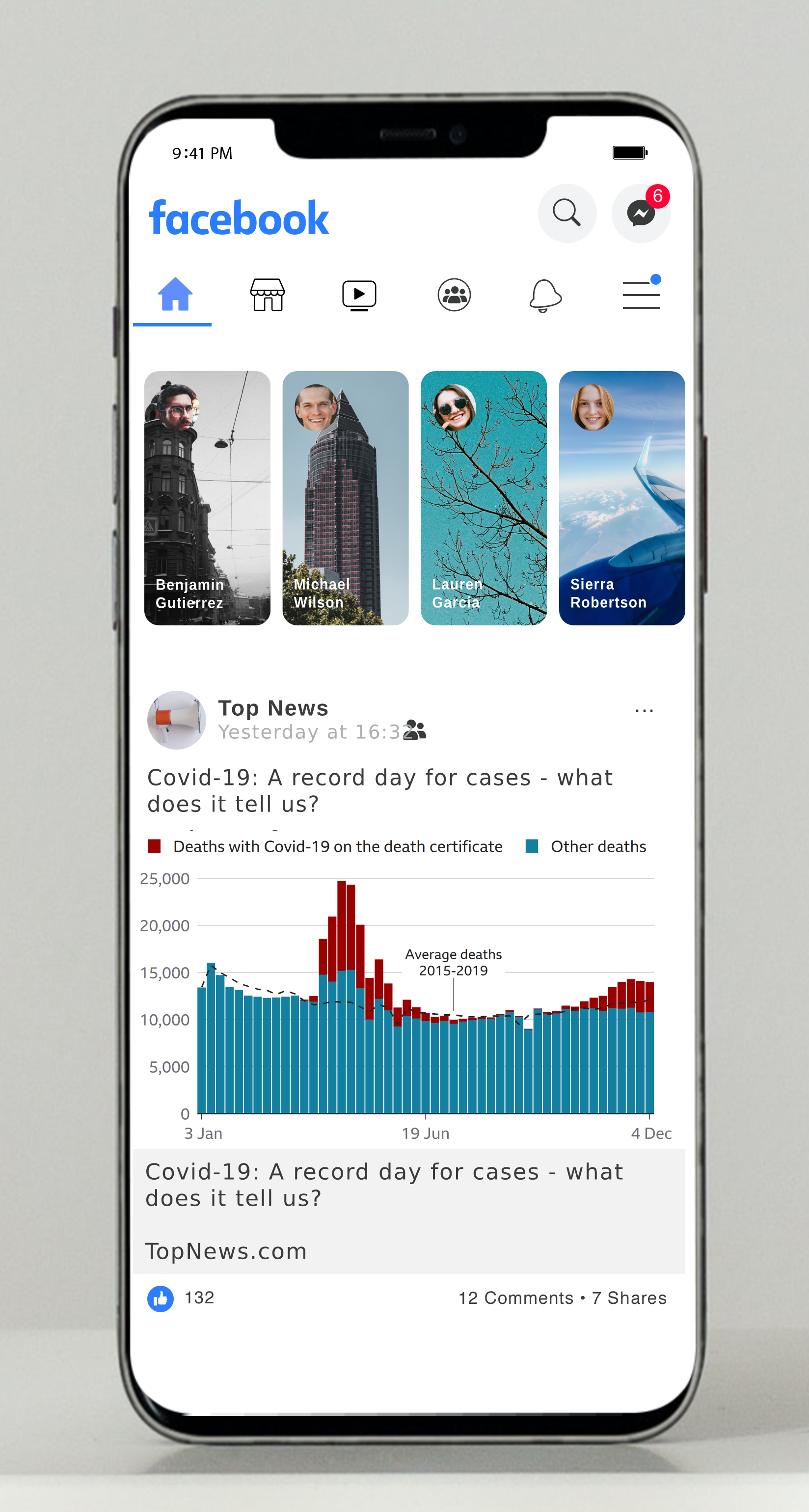

Supplement: Supplemental Information 5 [file peerj-cs-08-1153-s005.zip › PS2_Survey+stimuli/H5_V2.png]

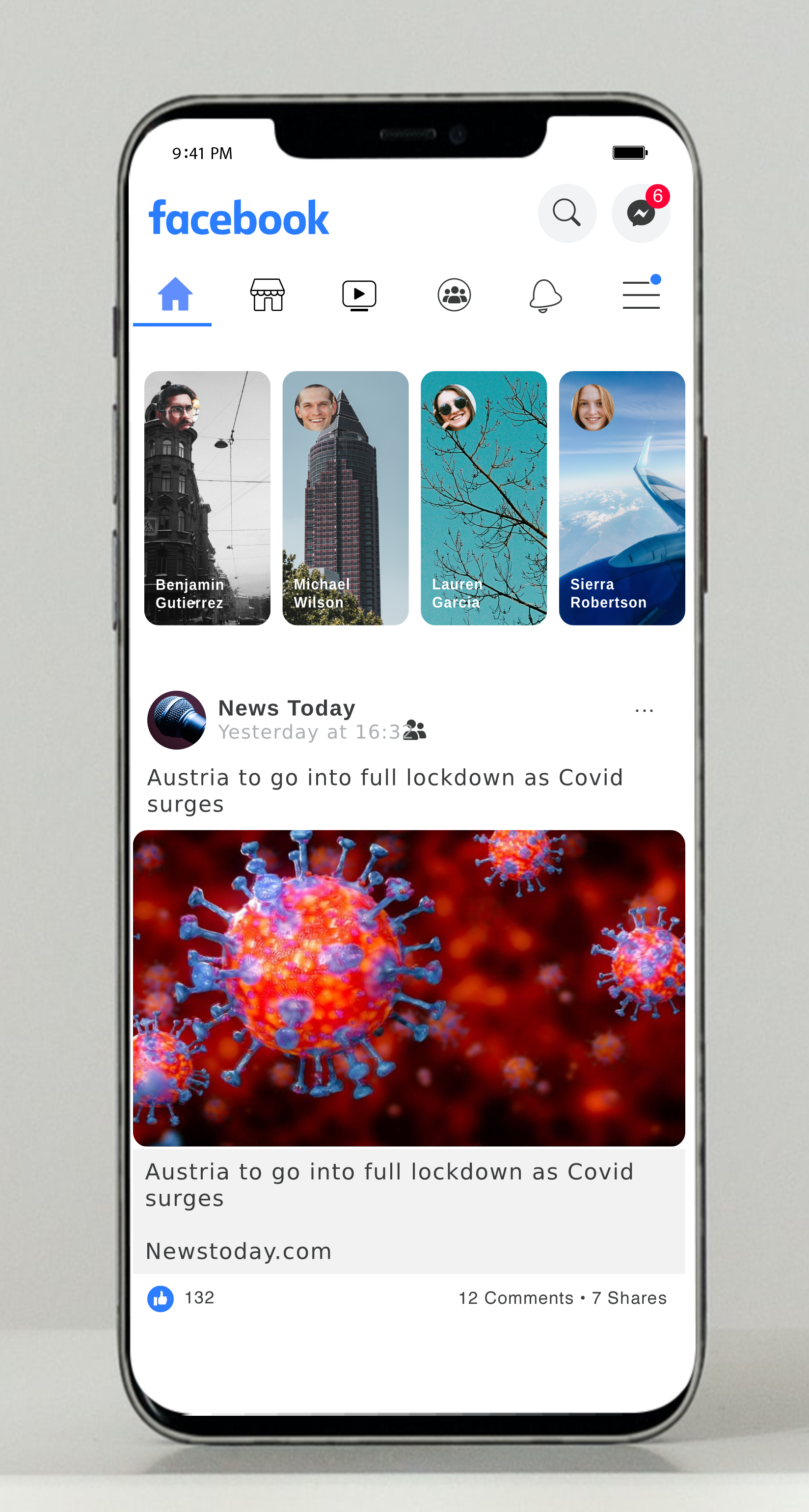

Supplement: Supplemental Information 5 [file peerj-cs-08-1153-s005.zip › PS2_Survey+stimuli/H6_B1.png]

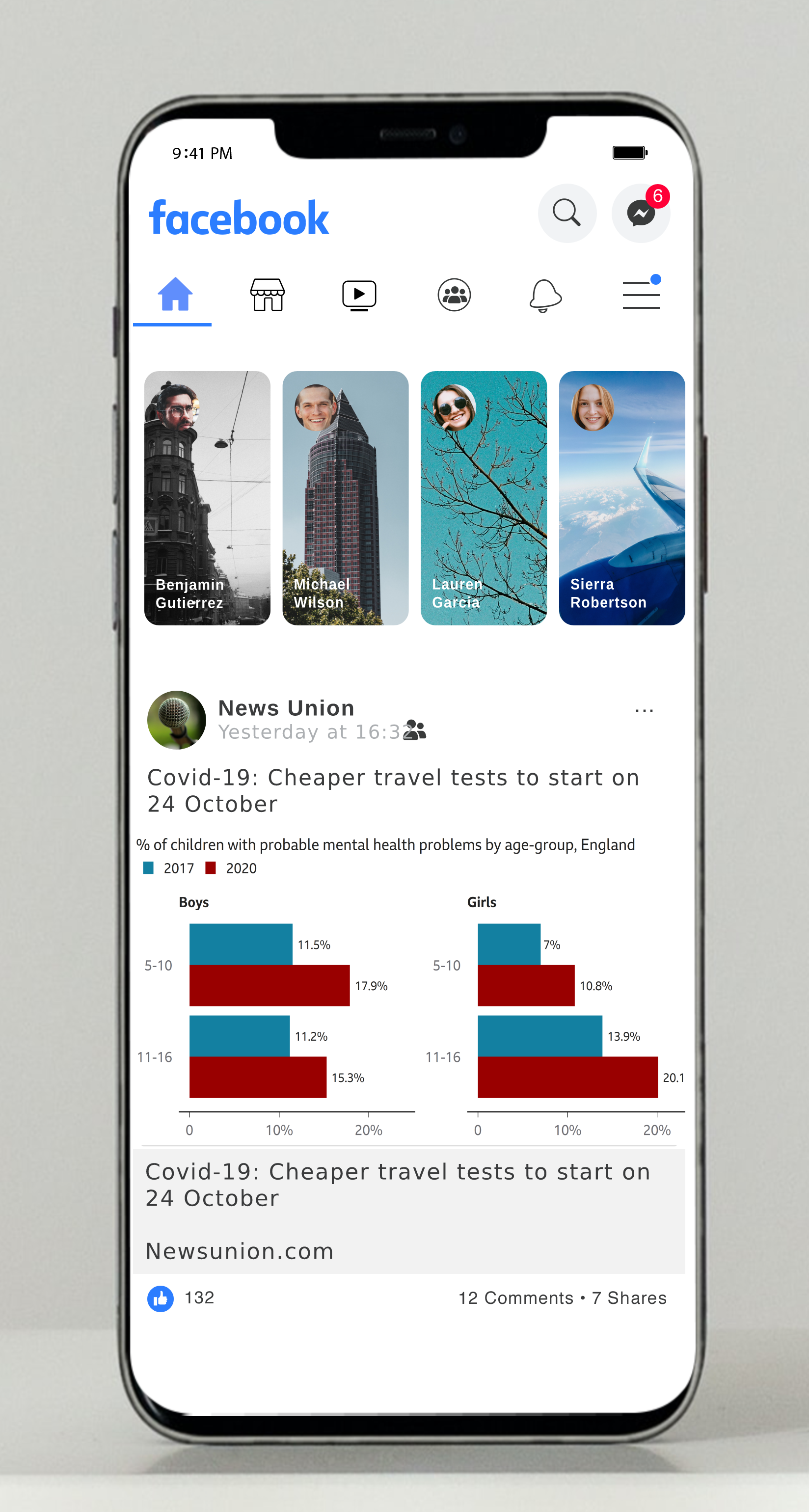

Supplement: Supplemental Information 5 [file peerj-cs-08-1153-s005.zip › PS2_Survey+stimuli/H7_V4.png]

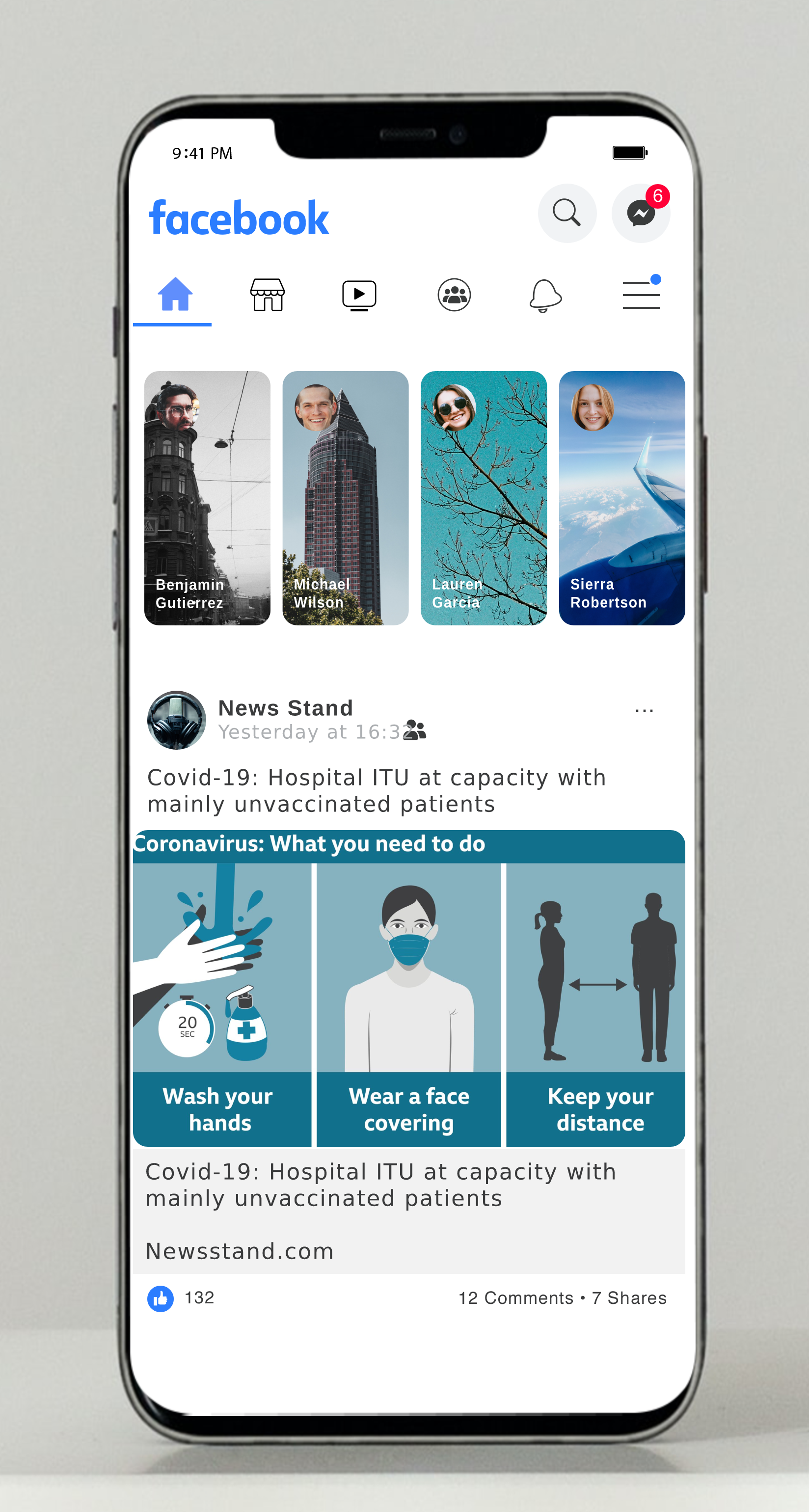

Supplement: Supplemental Information 5 [file peerj-cs-08-1153-s005.zip › PS2_Survey+stimuli/H8_A4.png]

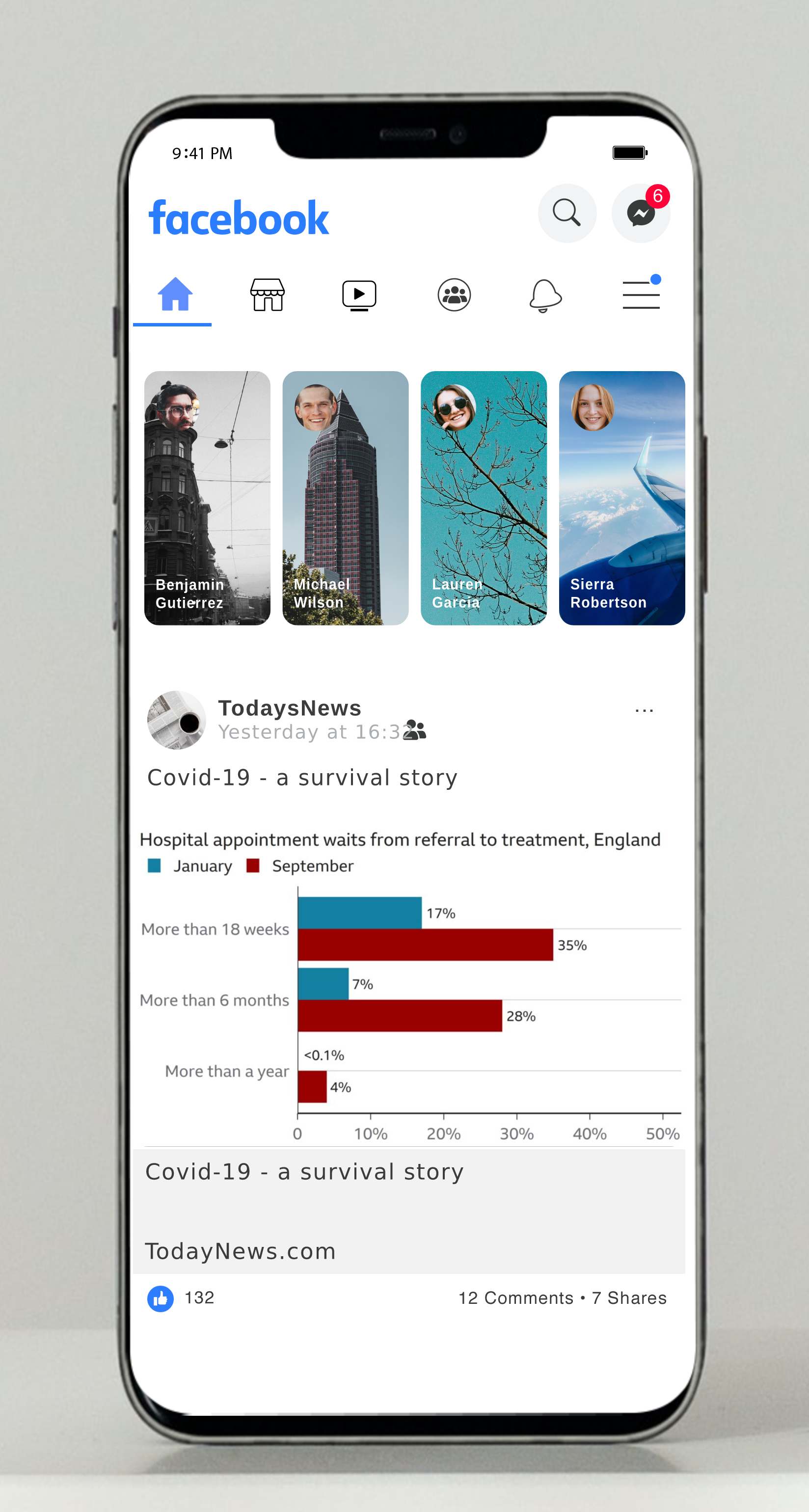

Supplement: Supplemental Information 5 [file peerj-cs-08-1153-s005.zip › PS2_Survey+stimuli/H9_V1.png]

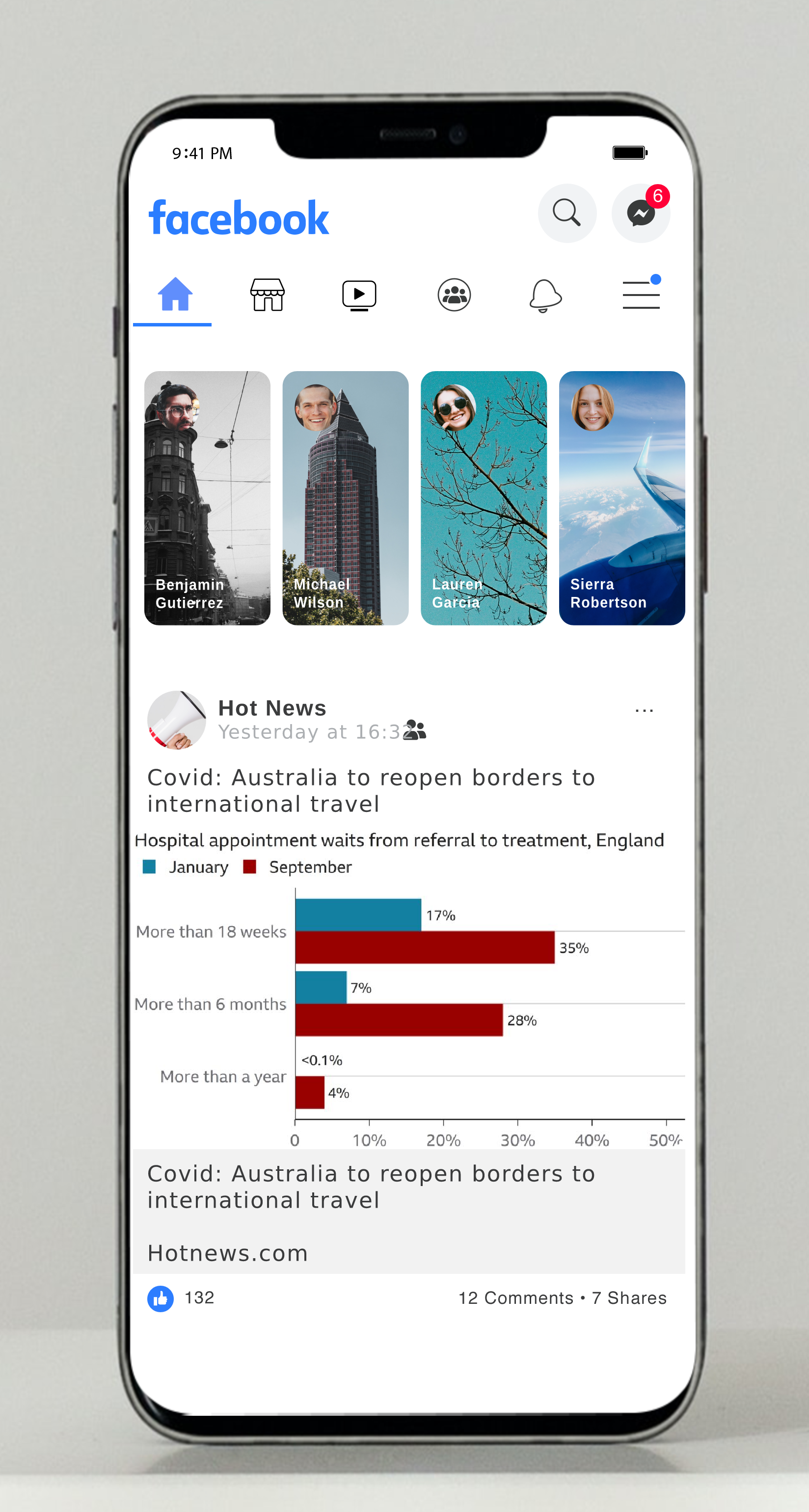

Supplement: Supplemental Information 6 [file peerj-cs-08-1153-s006.zip › PS3_Survey+stimuli/H1_V1.png]

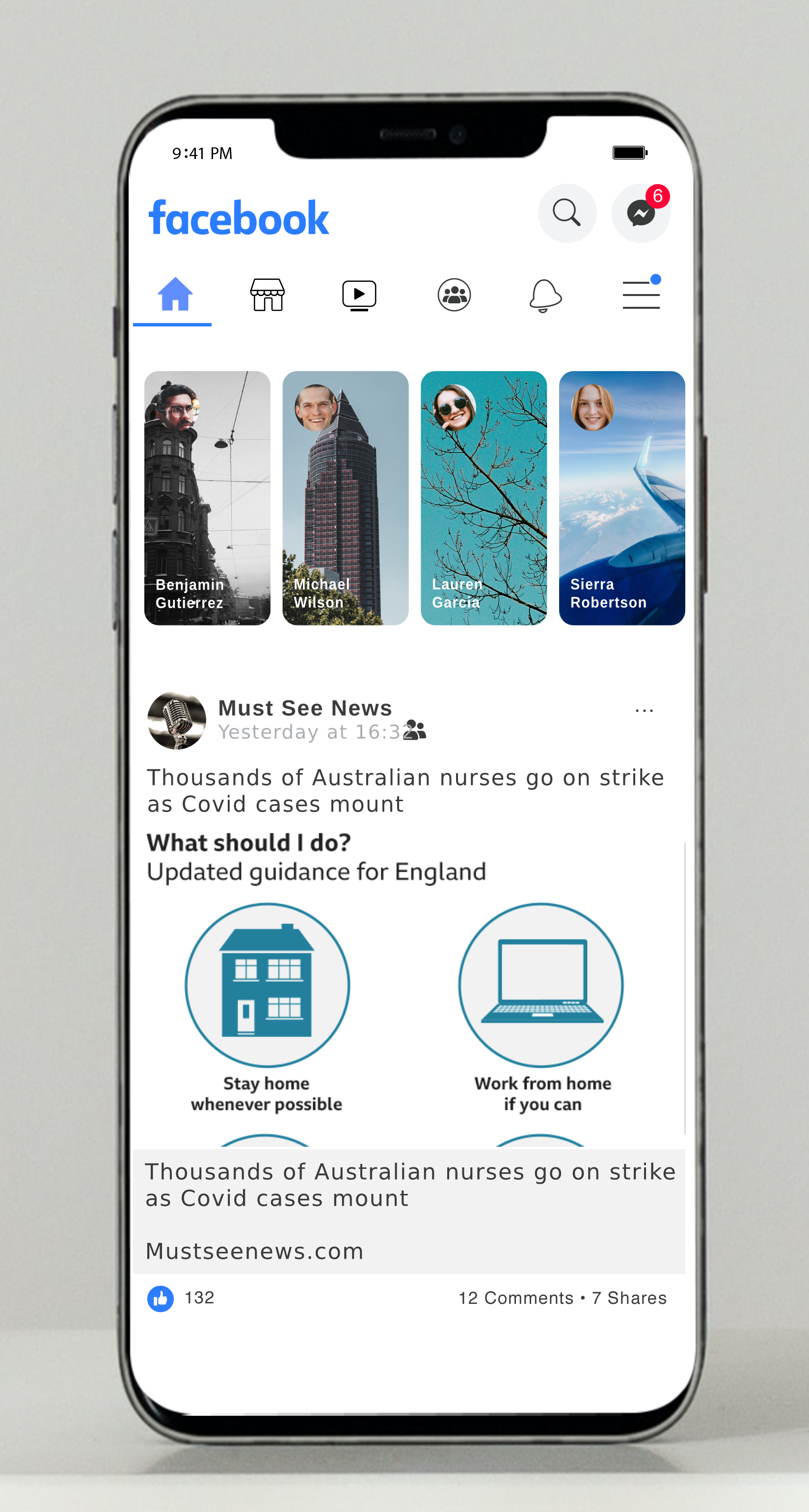

Supplement: Supplemental Information 6 [file peerj-cs-08-1153-s006.zip › PS3_Survey+stimuli/H10_A2.png]

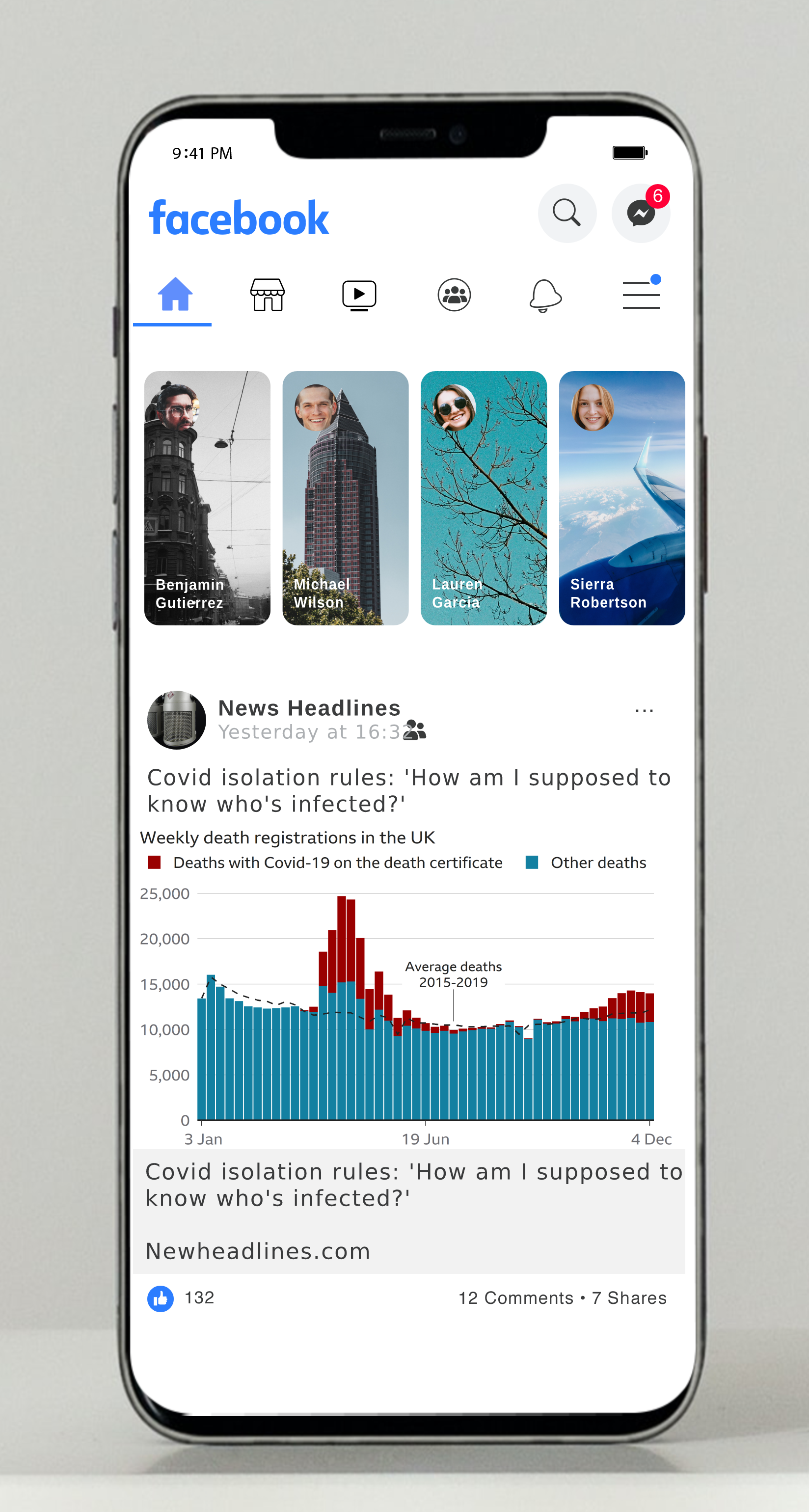

Supplement: Supplemental Information 6 [file peerj-cs-08-1153-s006.zip › PS3_Survey+stimuli/H11_V2.png]

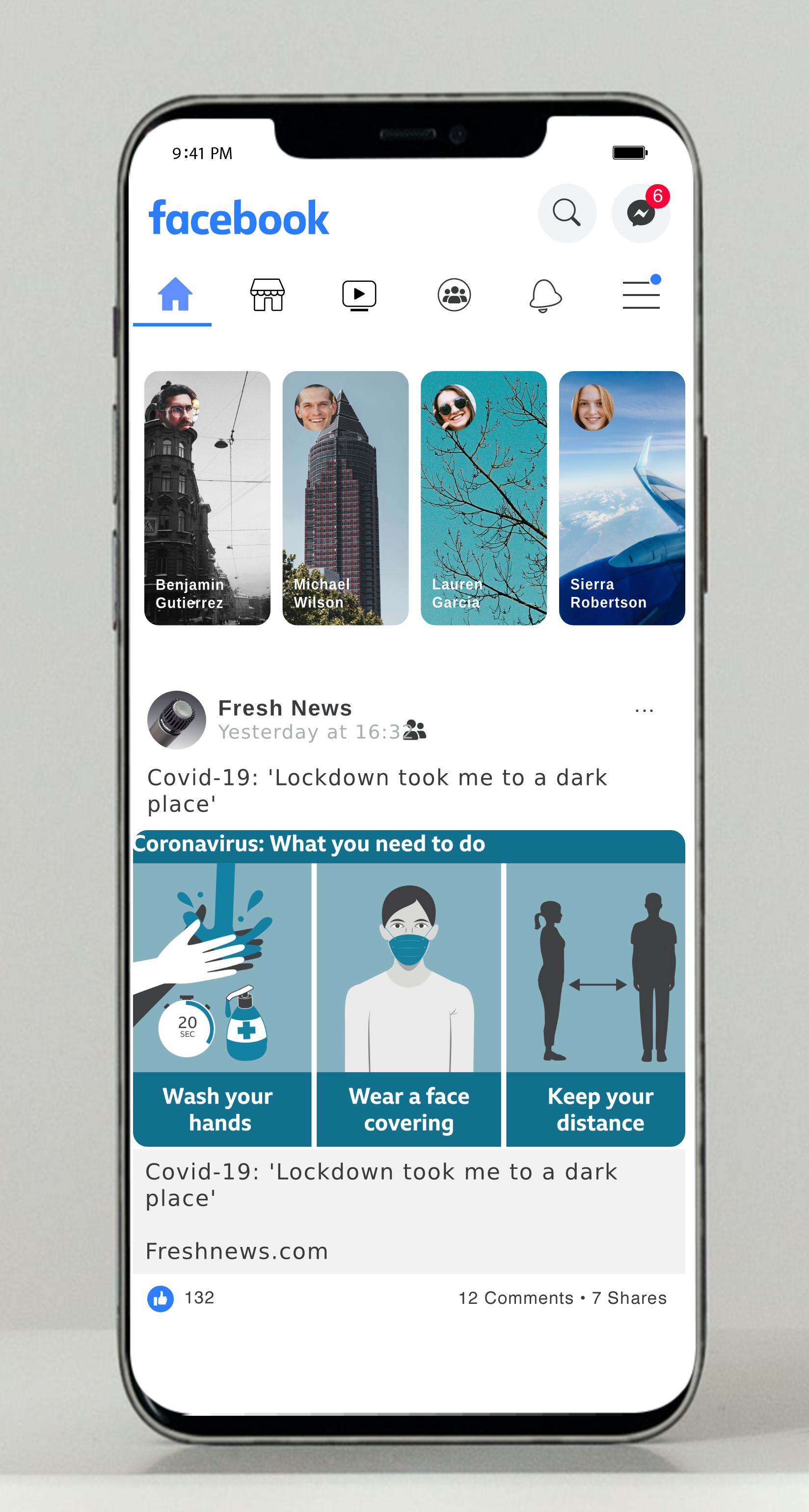

Supplement: Supplemental Information 6 [file peerj-cs-08-1153-s006.zip › PS3_Survey+stimuli/H3_A4.png]

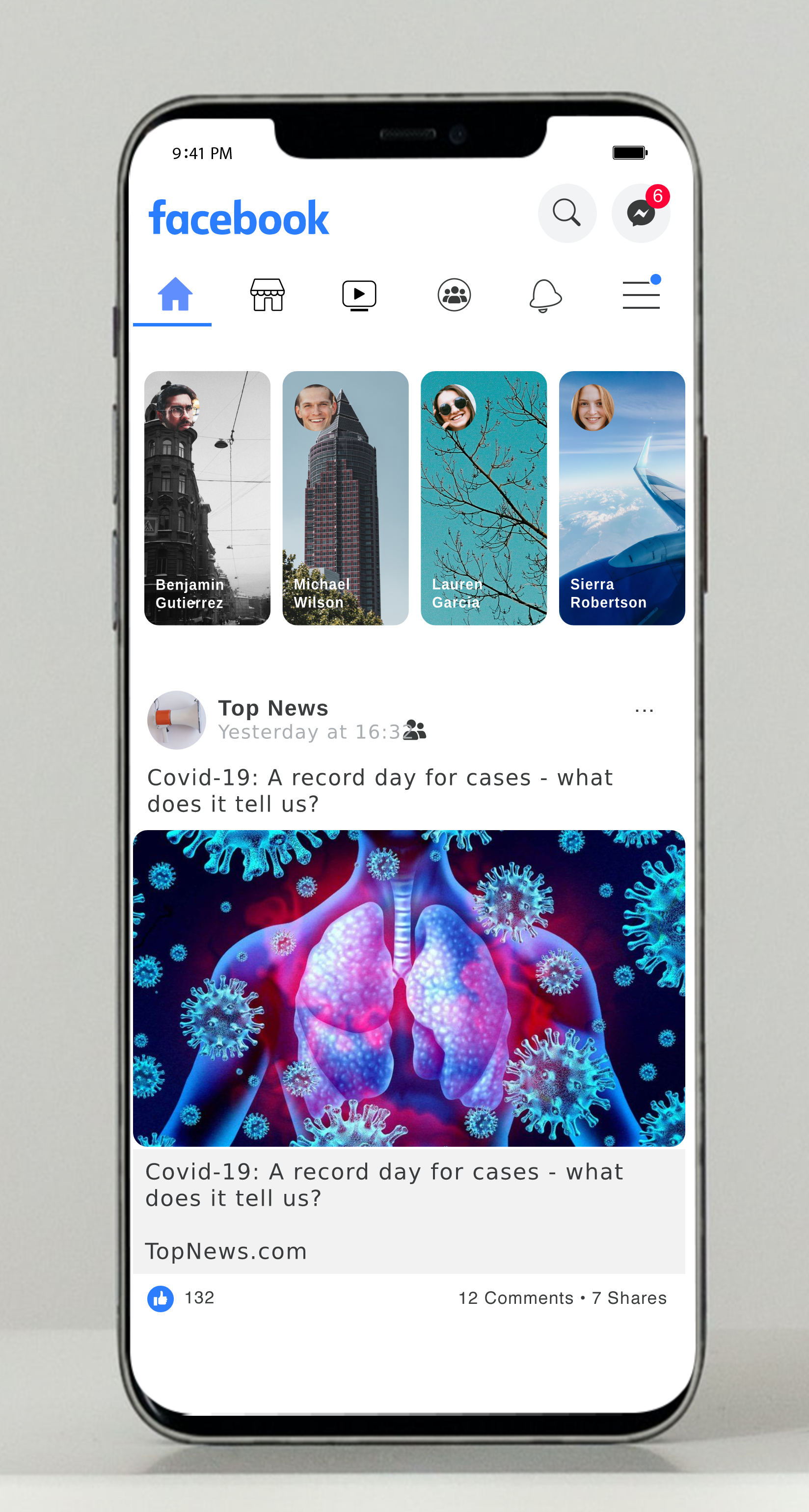

Supplement: Supplemental Information 6 [file peerj-cs-08-1153-s006.zip › PS3_Survey+stimuli/H5_B2.png]

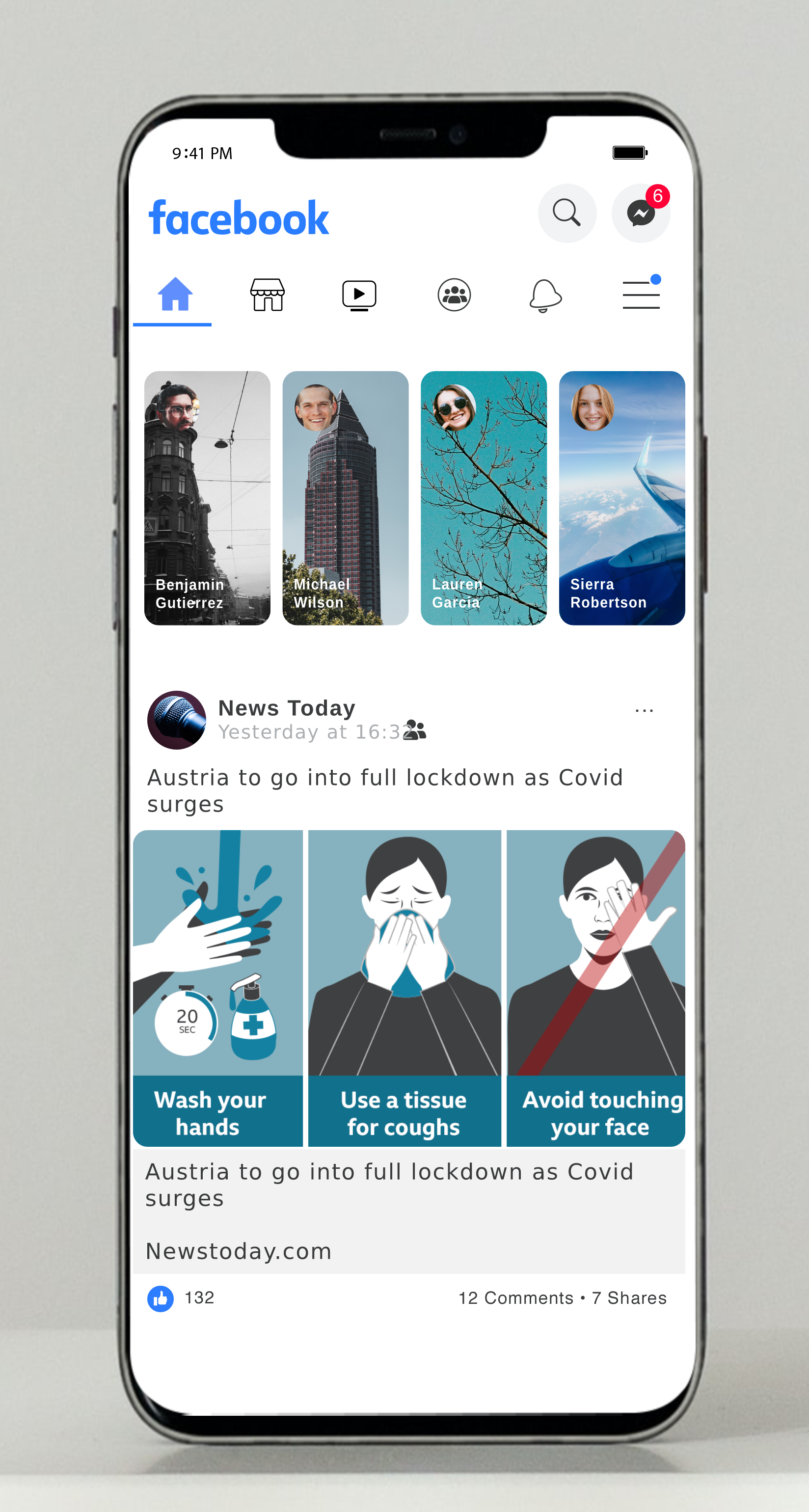

Supplement: Supplemental Information 6 [file peerj-cs-08-1153-s006.zip › PS3_Survey+stimuli/H6_A1.png]

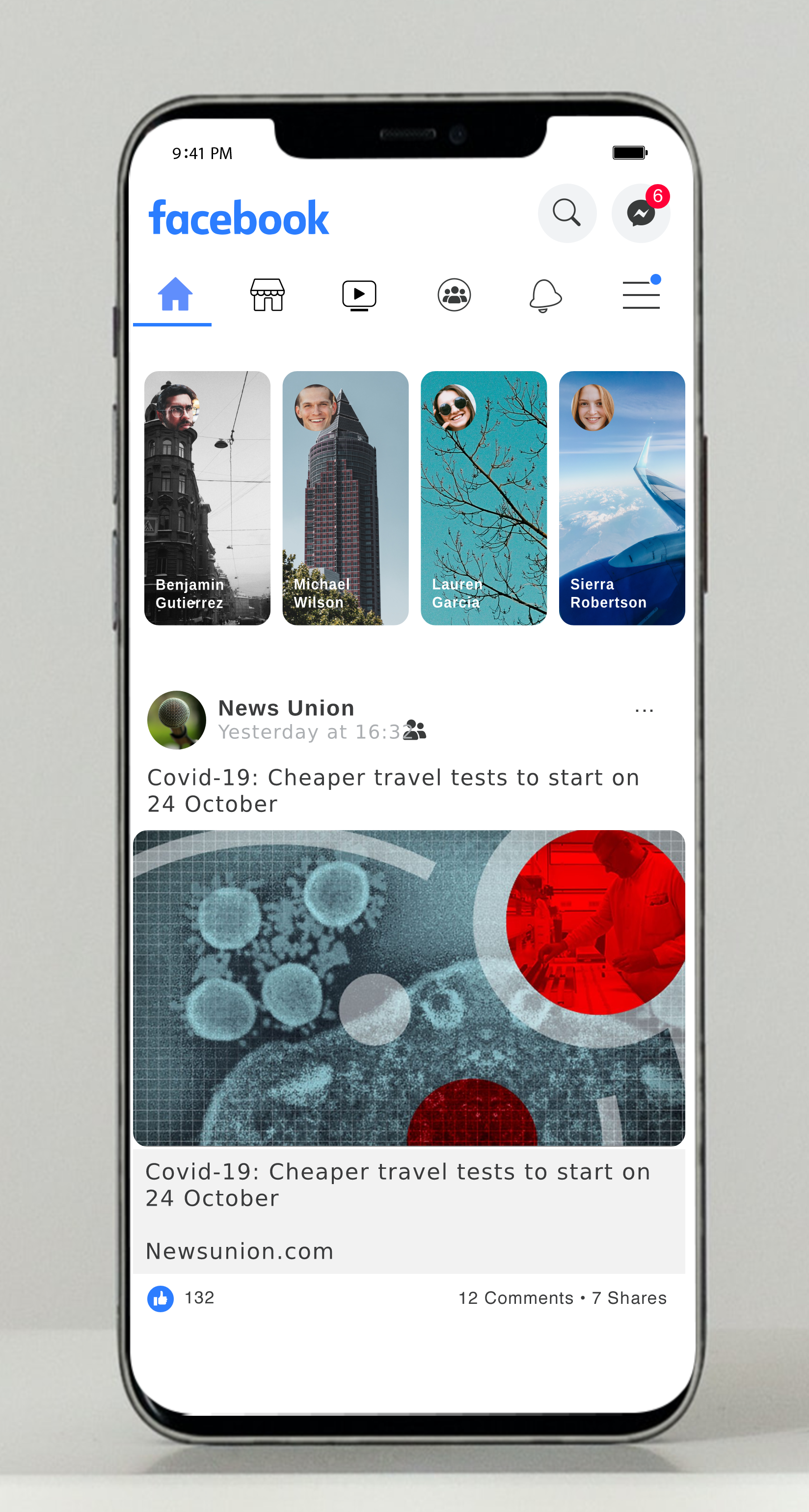

Supplement: Supplemental Information 6 [file peerj-cs-08-1153-s006.zip › PS3_Survey+stimuli/H7_B4.png]

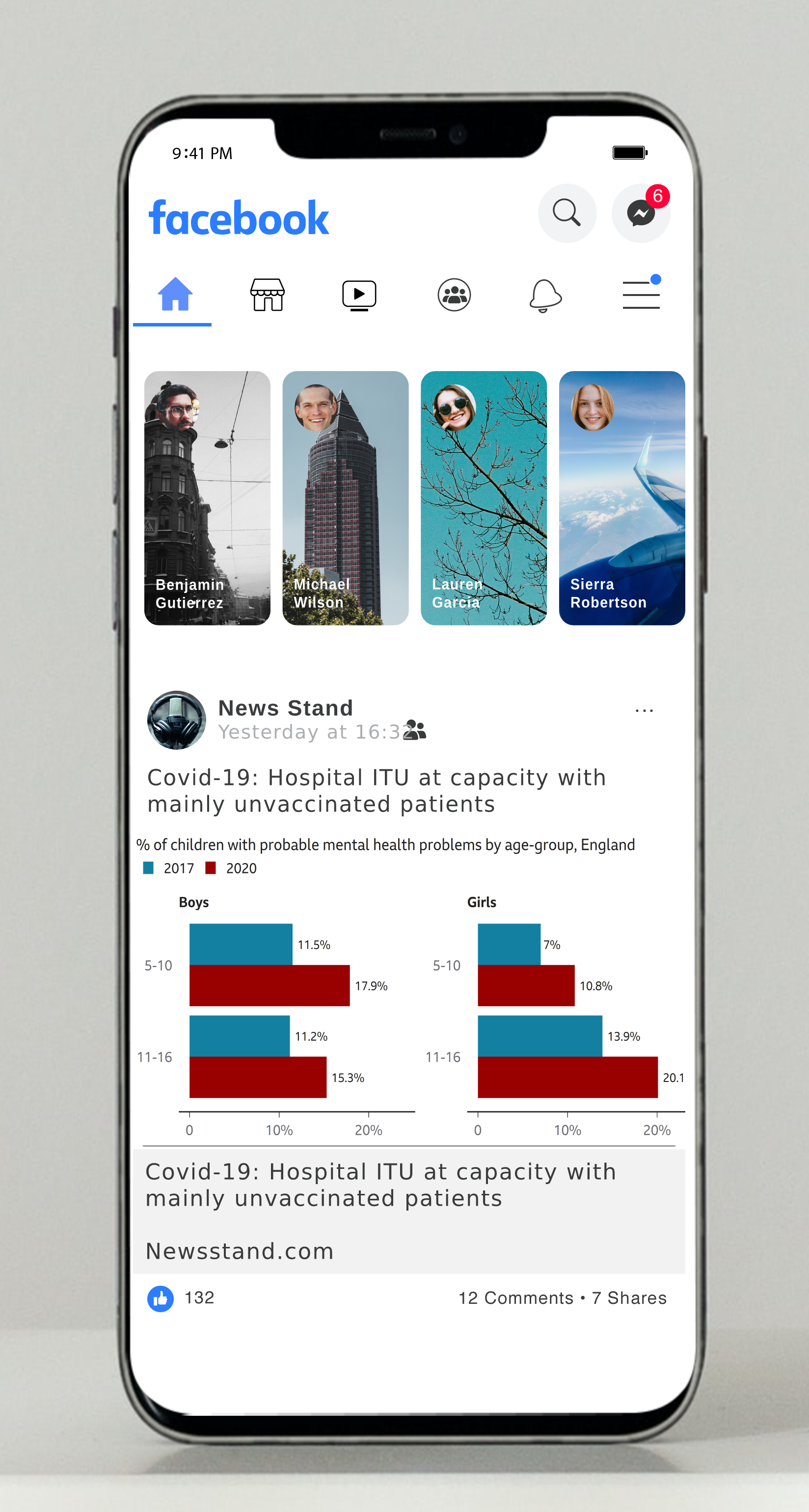

Supplement: Supplemental Information 6 [file peerj-cs-08-1153-s006.zip › PS3_Survey+stimuli/H8_V4.png]

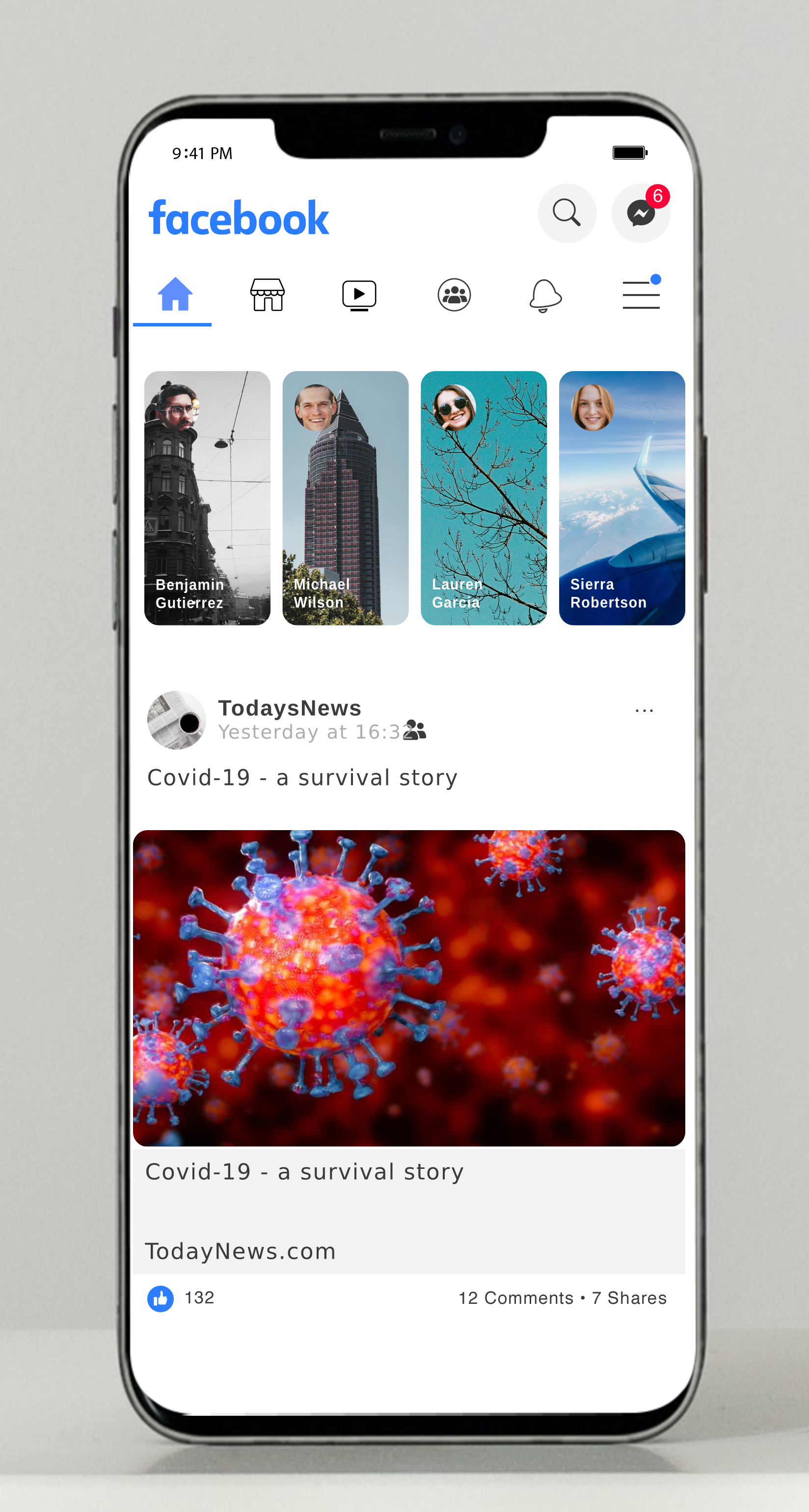

Supplement: Supplemental Information 6 [file peerj-cs-08-1153-s006.zip › PS3_Survey+stimuli/H9_B1.png]
