# Supplementary material for: Effect of visual imagery in COVID-19 social media posts on users’ perception
Source: PeerJ Comput Sci. 2022 Nov 15;8:e1153. doi: 10.7717/peerj-cs.1153 (PMC9680878; doi:10.7717/peerj-cs.1153)
Supplement: Supplemental Information 5 [file peerj-cs-08-1153-s005.zip › PS2_Survey+stimuli/survey_90443627_PS2.pdf]

SocialMediaRiskPerception-2

Survey Map

This survey is collecting data from you concerning the personal risk associated with browsing mobile-size social media posts about Covid-19. All questions are in the form of multiple-choice**.**

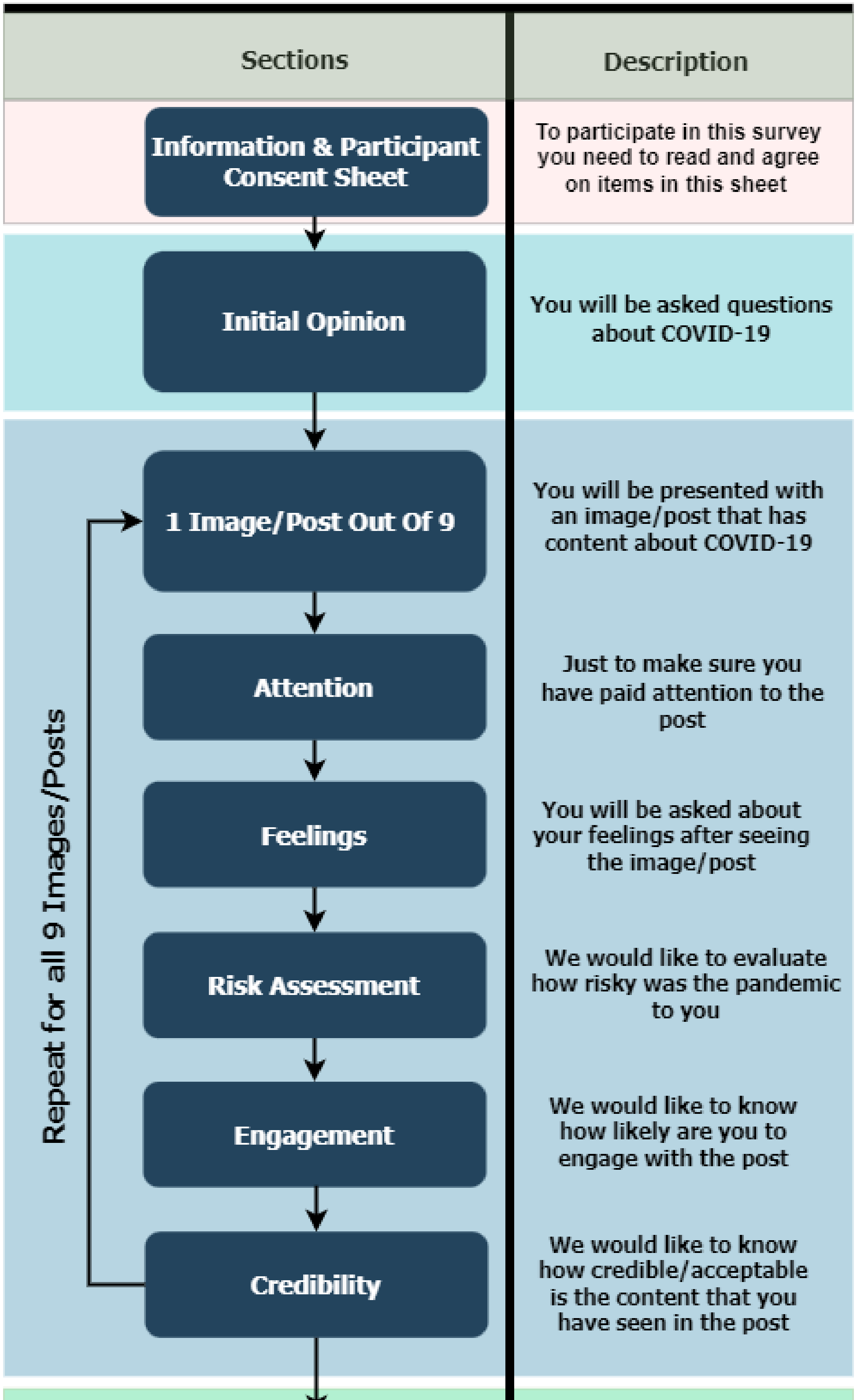

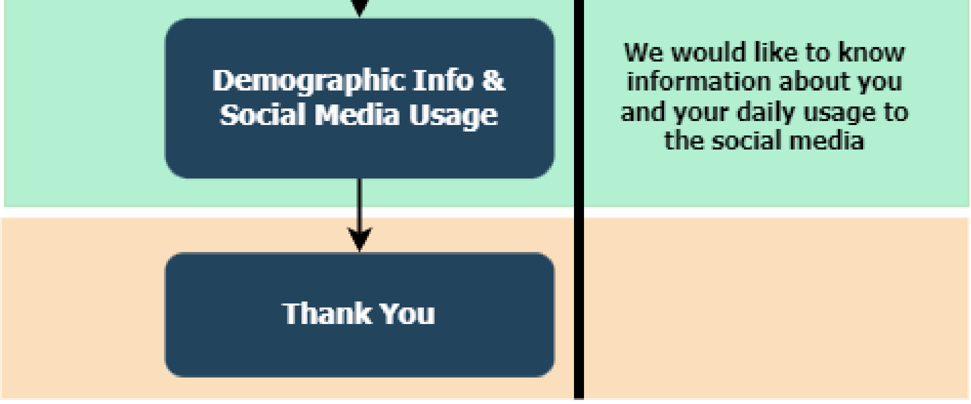

Information and Participant Consent Sheet

Please tick each point when you read it and agree on it: *

- ☐ **Purpose of the research:** Evaluating the associated personal risk when browsing mobile-size social media posts about Covid-19
- ☐ **Your role in this research:** if you decide to participate, you will complete a questionnaire based on your experience during the pandemic of Covid-19
- ☐ **Time Required:** The survey will take approximately 15 - 20 minutes to complete
- ☐ **Confidentiality:** Your responses will be kept anonymous. When research results are reported, responses will be aggregated (added together) and described in summary, with no single individual identifiable
- ☐ **Participation and Withdrawal:** Your participation would be highly appreciated. if you decide to participate, your participation is completely voluntary. You may refuse to participate or quite at any time and it is completely your choice
- ☐ **Repetition:** You may find some of the questions repetitive. However, due to the purpose of the research all questions must be answered
- ☐ **Attention:** You will be presented with 9 images/posts. Your attention and having a detailed look are required on each image/post before you proceed with the subsequent pages.

To contact the researcher: if you have any question or concerns about this research, please contact: Waleed Alnuwaiser, Email: waleednw@gmail.com

Initial Opinion Assessment

Do you find the COVID-19 pandemic important? *

	1	2	3	4	5	6	7	
**Not at all**	○	○	○	○	○	○	○	**Extremley**

What is your opinion on news' reportage on COVID-19? *

	-3	-2	-1	0	+1	+2	+3	
**Extremely Negative**	○	○	○	○	○	○	○	**Extremely Positive**

Have you been vaccinated against COVID-19? *

Yes	No
○	○

Image/Post

Please have a detailed look at the following image/post and tick 'I am done' when you finish:
*

☐ I am done

Attention test

Which human organ was presented in the last picture? *

- ○ Lungs
- ○ Heart
- ○ Stomach
- ○ Kidneys

Feelings

Rate how positive or negative this post made you feel, ranging from **'sad'** to **'happy'**:

*

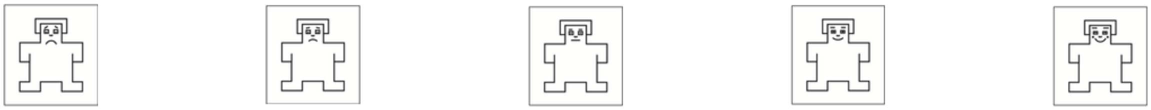

	1	2	3	4	5	6	7	8	9	
**Sad**	○	○	○	○	○	○	○	○	○	**Happy**

Rate how energetic this post made you feel, ranging from **'sleepy/apathetic'** to **'excited/energetic' :** *

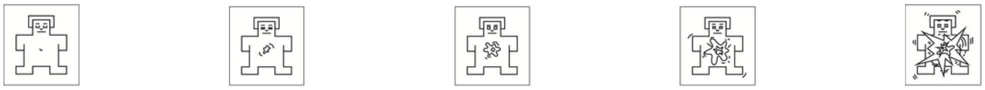

	1	2	3	4	5	6	7	8	9	
**Sleepy/Apathetic**	○	○	○	○	○	○	○	○	○	**Excited**

Rate how in control of the situation this post made you feel, ranging from **'no control'** to **'completely in control':** *

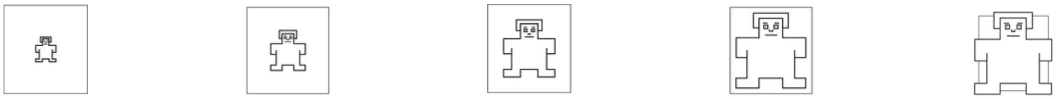

	1	2	3	4	5	6	7	8	9	
**No control**	○	○	○	○	○	○	○	○	○	**Completely in control**

Risk Assessment

After seeing this news post, how much anxiety do you feel in regards to the COVID-19 pandemic?*

	1	2	3	4	5	
**No anxiety at all**	○	○	○	○	○	**Very high anxiety**

After seeing the news post, how big of a risk do you think the COVID-19 pandemic poses to you or your family?*

	1	2	3	4	5	
**No risk at all**	○	○	○	○	○	**Very high risk**

Where 'dread' means to be in terror of, or fear intensely, how much do you dread the COVID-19 pandemic after seeing this news post?
*

	1	2	3	4	5	
**No dread at all**	○	○	○	○	○	**Very high dread**

After seeing the news post, what do you think is your likelihood of infection?

	1	2	3	4	5	
**Very unlikely**	○	○	○	○	○	**Very likely**

After seeing the news post, how harmful do you think it would be if you got infected?*

	1	2	3	4	5	
**Not harmful at all**	○	○	○	○	○	**Very harmful**

Engagement

Are you likely to… (check where applicable)?*

- ☐ Share the post
- ☐ Read the post
- ☐ Comment on the post
- ☐ Ignore the post

Credibility

... can be trusted *

	1	2	3	4	5	
**Strongly disagree**	○	○	○	○	○	**Strongly agree**

...separates facts from opinion *

	1	2	3	4	5	
**Strongly disagree**	○	○	○	○	○	**Strongly agree**

...is factual *

	1	2	3	4	5	
**Strongly disagree**	○	○	○	○	○	**Strongly agree**

...tells the whole strory *

	1	2	3	4	5	
**Strongly disagree**	○	○	○	○	○	**Strongly agree**

...is accurate *

	1	2	3	4	5	
**Strongly disagree**	○	○	○	○	○	**Strongly agree**

...is unbiased *

	1	2	3	4	5	
**Strongly disagree**	○	○	○	○	○	**Strongly agree**

...is fair *

	1	2	3	4	5	
**Strongly disagree**	○	○	○	○	○	**Strongly agree**

... demonstrates concern about public interest *

	1	2	3	4	5	
**Strongly disagree**	○	○	○	○	○	**Strongly agree**

...is sensationalized *

	1	2	3	4	5	
**Strongly disagree**	○	○	○	○	○	**Strongly agree**

...is immoral *

	1	2	3	4	5	
**Strongly disagree**	○	○	○	○	○	**Strongly agree**

Image/Post

Please have a detailed look at the following image/post and tick 'I am done' when you finish:
*

☐ I am done

Attention test

Which colors were presented in the last picture? *

- ○ Red and Blue/Gey
- ○ Yellow and Red
- ○ Black and White
- ○ Purple and Pink

Feelings

Rate how positive or negative this post made you feel, ranging from **'sad'** to **'happy'**:

*

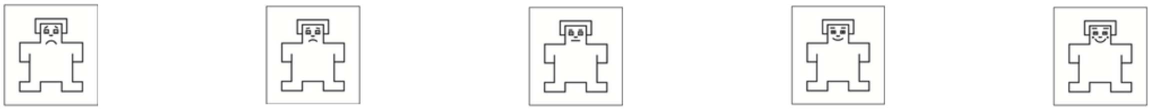

	1	2	3	4	5	6	7	8	9	
**Sad**	○	○	○	○	○	○	○	○	○	**Happy**

Rate how energetic this post made you feel, ranging from **'sleepy/apathetic'** to **'excited/energetic' :** *

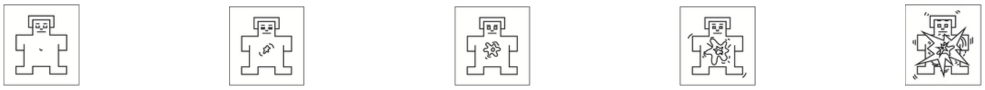

	1	2	3	4	5	6	7	8	9	
**Sleepy/Apathetic**	○	○	○	○	○	○	○	○	○	**Excited**

Rate how in control of the situation this post made you feel, ranging from **'no control'** to **'completely in control'**: *

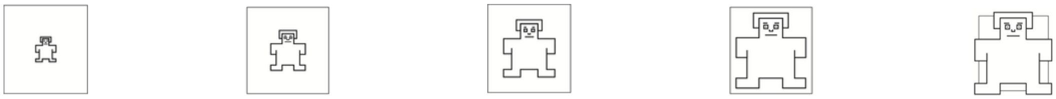

	1	2	3	4	5	6	7	8	9	
**No control**	○	○	○	○	○	○	○	○	○	**Completely in control**

Risk Assessment

After seeing this news post, how much anxiety do you feel in regards to the COVID-19 pandemic?*

	1	2	3	4	5	
**No anxiety at all**	○	○	○	○	○	**Very high anxiety**

After seeing the news post, how big of a risk do you think the COVID-19 pandemic poses to you or your family?*

	1	2	3	4	5	
**No risk at all**	○	○	○	○	○	**Very high risk**

Where 'dread' means to be in terror of, or fear intensely, how much do you dread the COVID-19 pandemic after seeing this news post?
*

	1	2	3	4	5	
**No dread at all**	○	○	○	○	○	**Very high dread**

After seeing the news post, what do you think is your likelihood of infection?*

	1	2	3	4	5	
**Very unlikely**	○	○	○	○	○	**Very likely**

After seeing the news post, how harmful do you think it would be if you got infected?*

	1	2	3	4	5	
**Not harmful at all**	○	○	○	○	○	**Very harmful**

Engagement

Are you likely to… (check where applicable)? *

- [ ] Share the post
- [ ] Read the post
- [ ] Comment on the post
- [ ] Ignore the post

Credibility

... can be trusted *

	1	2	3	4	5	
**Strongly disagree**	○	○	○	○	○	**Strongly agree**

...separates facts from opinion *

	1	2	3	4	5	
**Strongly disagree**	○	○	○	○	○	**Strongly agree**

...is factual *

	1	2	3	4	5	
**Strongly disagree**	○	○	○	○	○	**Strongly agree**

...tells the whole strory *

	1	2	3	4	5	
**Strongly disagree**	○	○	○	○	○	**Strongly agree**

...is accurate *

	1	2	3	4	5	
**Strongly disagree**	○	○	○	○	○	**Strongly agree**

...is unbiased *

	1	2	3	4	5	
**Strongly disagree**	○	○	○	○	○	**Strongly agree**

...is fair *

	1	2	3	4	5	
**Strongly disagree**	○	○	○	○	○	**Strongly agree**

... demonstrates concern about public interest *

	1	2	3	4	5	
**Strongly disagree**	○	○	○	○	○	**Strongly agree**

...is sensationalized *

	1	2	3	4	5	
**Strongly disagree**	○	○	○	○	○	**Strongly agree**

...is immoral *

	1	2	3	4	5	
**Strongly disagree**	○	○	○	○	○	**Strongly agree**

Image/Post

Please have a detailed look at the following image/post and tick 'I am done' when you finish:
*

☐ I am done

Attention test

What was the color of the spikes in the last picture? *

- Green
- Yellow
- Blue
- Black

Feelings

Rate how positive or negative this post made you feel, ranging from **'sad'** to **'happy'**:

*

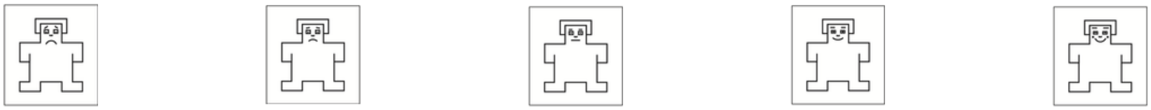

	1	2	3	4	5	6	7	8	9	
**Sad**										**Happy**

Rate how energetic this post made you feel, ranging from **'sleepy/apathetic'** to **'excited/energetic'** : *

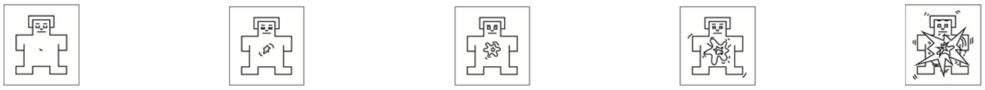

	1	2	3	4	5	6	7	8	9	
**Sleepy/Apathetic**										**Excited**

Rate how in control of the situation this post made you feel, ranging from **'no control'** to **'completely in control'**: *

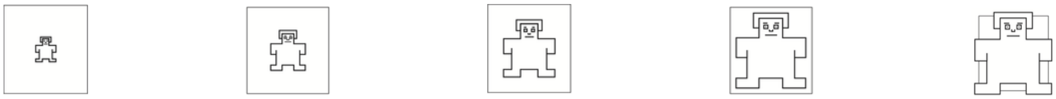

	1	2	3	4	5	6	7	8	9	
**No control**										**Completely in control**

Risk Assessment

After seeing this news post, how much anxiety do you feel in regards to the COVID-19 pandemic?*

	1	2	3	4	5	
**No anxiety at all**						**Very high anxiety**

After seeing the news post, how big of a risk do you think the COVID-19 pandemic poses to you or your family?*

	1	2	3	4	5	
**No risk at all**	○	○	○	○	○	**Very high risk**

Where 'dread' means to be in terror of, or fear intensely, how much do you dread the COVID-19 pandemic after seeing this news post?
*

	1	2	3	4	5	
**No dread at all**	○	○	○	○	○	**Very high dread**

After seeing the news post, what do you think is your likelihood of infection?*

	1	2	3	4	5	
**Very unlikely**	○	○	○	○	○	**Very likely**

After seeing the news post, how harmful do you think it would be if you got infected?*

	1	2	3	4	5	
**Not harmful at all**	○	○	○	○	○	**Very harmful**

Engagement

Are you likely to… (check where applicable)?*

- ☐ Share the post
- ☐ Read the post
- ☐ Comment on the post
- ☐ Ignore the post

Credibility

... can be trusted *

	1	2	3	4	5	
**Strongly disagree**	○	○	○	○	○	**Strongly agree**

...separates facts from opinion *

	1	2	3	4	5	
**Strongly disagree**	○	○	○	○	○	**Strongly agree**

...is factual *

	1	2	3	4	5	
**Strongly disagree**	○	○	○	○	○	**Strongly agree**

...tells the whole strory *

	1	2	3	4	5	
**Strongly disagree**	○	○	○	○	○	**Strongly agree**

...is accurate *

	1	2	3	4	5	
**Strongly disagree**	○	○	○	○	○	**Strongly agree**

...is unbiased *

	1	2	3	4	5	
**Strongly disagree**	○	○	○	○	○	**Strongly agree**

...is fair *

	1	2	3	4	5	
**Strongly disagree**	○	○	○	○	○	**Strongly agree**

... demonstrates concern about public interest *

	1	2	3	4	5	
**Strongly disagree**	○	○	○	○	○	**Strongly agree**

...is sensationalized *

	1	2	3	4	5	
**Strongly disagree**	○	○	○	○	○	**Strongly agree**

...is immoral *

	1	2	3	4	5	
**Strongly disagree**	○	○	○	○	○	**Strongly agree**

Image/Post

Please have a detailed look at the following image/post and tick 'I am done' when you finish:
*

- [ ] I am done

Attention test

For what amount of time did the picture advice you to wash hands? *

- ○ 10 seconds
- ○ 20 seconds
- ○ 40 seconds
- ○ 60 seconds

Feelings

Rate how positive or negative this post made you feel, ranging from **'sad'** to **'happy'**:

*

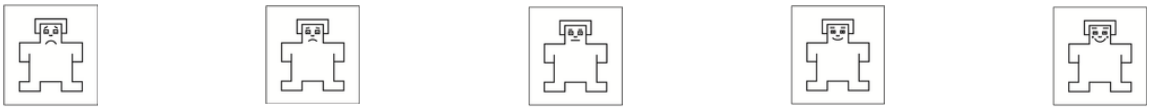

	1	2	3	4	5	6	7	8	9	
**Sad**	○	○	○	○	○	○	○	○	○	**Happy**

Rate how energetic this post made you feel, ranging from **'sleepy/apathetic'** to **'excited/energetic' :** *

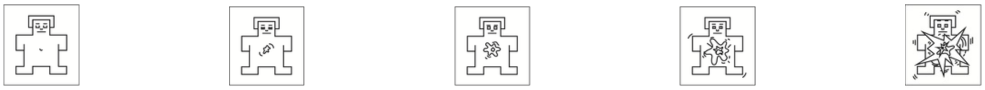

	1	2	3	4	5	6	7	8	9	
**Sleepy/Apathetic**	○	○	○	○	○	○	○	○	○	**Excited**

Rate how in control of the situation this post made you feel, ranging from **'no control'** to **'completely in control':** *

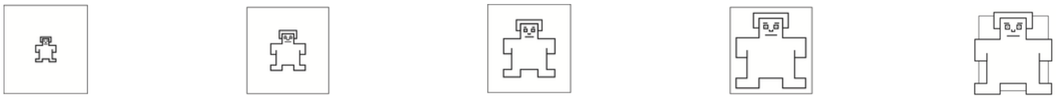

	1	2	3	4	5	6	7	8	9	
**No control**	○	○	○	○	○	○	○	○	○	**Completely in control**

Risk Assessment

After seeing this news post, how much anxiety do you feel in regards to the COVID-19 pandemic?*

	1	2	3	4	5	
**No anxiety at all**	○	○	○	○	○	**Very high anxiety**

After seeing the news post, how big of a risk do you think the COVID-19 pandemic poses to you or your family?*

	1	2	3	4	5	
**No risk at all**	○	○	○	○	○	**Very high risk**

Where ‘dread’ means to be in terror of, or fear intensely, how much do you dread the COVID-19 pandemic after seeing this news post?
*

	1	2	3	4	5	
**No dread at all**	○	○	○	○	○	**Very high dread**

After seeing the news post, what do you think is your likelihood of infection?*

	1	2	3	4	5	
**Very unlikely**	○	○	○	○	○	**Very likely**

After seeing the news post, how harmful do you think it would be if you got infected?*

	1	2	3	4	5	
**Not harmful at all**	○	○	○	○	○	**Very harmful**

Engagement

Are you likely to… (check where applicable)?*

- ☐ Share the post
- ☐ Read the post
- ☐ Comment on the post
- ☐ Ignore the post

Credibility

... can be trusted *

	1	2	3	4	5	
**Strongly disagree**	○	○	○	○	○	**Strongly agree**

...separates facts from opinion *

	1	2	3	4	5	
**Strongly disagree**	○	○	○	○	○	**Strongly agree**

...is factual *

	1	2	3	4	5	
**Strongly disagree**	○	○	○	○	○	**Strongly agree**

...tells the whole strory *

	1	2	3	4	5	
**Strongly disagree**	○	○	○	○	○	**Strongly agree**

...is accurate *

	1	2	3	4	5	
**Strongly disagree**	○	○	○	○	○	**Strongly agree**

...is unbiased *

	1	2	3	4	5	
**Strongly disagree**	○	○	○	○	○	**Strongly agree**

...is fair *

	1	2	3	4	5	
**Strongly disagree**	○	○	○	○	○	**Strongly agree**

... demonstrates concern about public interest *

	1	2	3	4	5	
**Strongly disagree**	○	○	○	○	○	**Strongly agree**

...is sensationalized *

	1	2	3	4	5	
**Strongly disagree**	○	○	○	○	○	**Strongly agree**

...is immoral *

	1	2	3	4	5	
**Strongly disagree**	○	○	○	○	○	**Strongly agree**

Image/Post

Please have a detailed look at the following image/post and tick 'I am done' when you finish:
*

☐ I am done

Attention test

For which part of the United Kingdom the recommendations/guidance were given in the last picture? *

○ England

○ Wales

○ Scotland

Feelings

Rate how positive or negative this post made you feel, ranging from **'sad'** to **'happy'**:

*

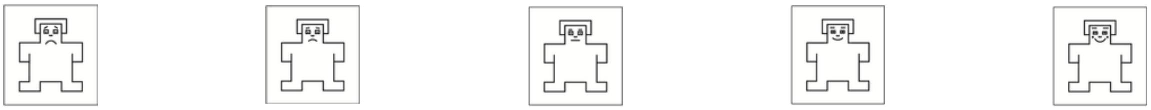

	1	2	3	4	5	6	7	8	9	
**Sad**	○	○	○	○	○	○	○	○	○	**Happy**

Rate how energetic this post made you feel, ranging from **'sleepy/apathetic'** to **'excited/energetic'** : *

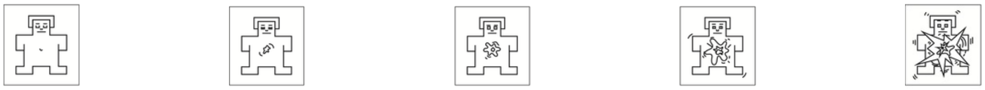

	1	2	3	4	5	6	7	8	9	
**Sleepy/Apathetic**	○	○	○	○	○	○	○	○	○	**Excited**

Rate how in control of the situation this post made you feel, ranging from **'no control'** to **'completely in control'**: *

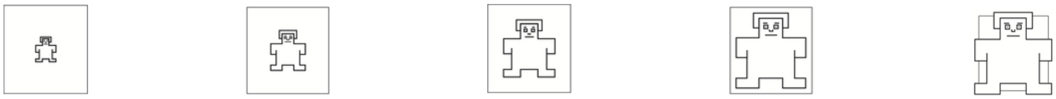

	1	2	3	4	5	6	7	8	9	
**No control**	○	○	○	○	○	○	○	○	○	**Completely in control**

Risk Assessment

After seeing this news post, how much anxiety do you feel in regards to the COVID-19 pandemic?*

	1	2	3	4	5	
**No anxiety at all**	○	○	○	○	○	**Very high anxiety**

After seeing the news post, how big of a risk do you think the COVID-19 pandemic poses to you or your family?*

	1	2	3	4	5	
**No risk at all**	○	○	○	○	○	**Very high risk**

Where ‘dread’ means to be in terror of, or fear intensely, how much do you dread the COVID-19 pandemic after seeing this news post?
*

	1	2	3	4	5	
**No dread at all**	○	○	○	○	○	**Very high dread**

After seeing the news post, what do you think is your likelihood of infection?*

	1	2	3	4	5	
**Very unlikely**	○	○	○	○	○	**Very likely**

After seeing the news post, how harmful do you think it would be if you got infected?*

	1	2	3	4	5	
**Not harmful at all**	○	○	○	○	○	**Very harmful**

Engagement

Are you likely to… (check where applicable)?*

- ☐ Share the post
- ☐ Read the post
- ☐ Comment on the post
- ☐ Ignore the post

Credibility

... can be trusted *

	1	2	3	4	5	
**Strongly disagree**	○	○	○	○	○	**Strongly agree**

...separates facts from opinion *

	1	2	3	4	5	
**Strongly disagree**	○	○	○	○	○	**Strongly agree**

...is factual *

	1	2	3	4	5	
**Strongly disagree**	○	○	○	○	○	**Strongly agree**

...tells the whole strory *

	1	2	3	4	5	
**Strongly disagree**	○	○	○	○	○	**Strongly agree**

...is accurate *

	1	2	3	4	5	
**Strongly disagree**	○	○	○	○	○	**Strongly agree**

...is unbiased *

	1	2	3	4	5	
**Strongly disagree**	○	○	○	○	○	**Strongly agree**

...is fair *

	1	2	3	4	5	
**Strongly disagree**	○	○	○	○	○	**Strongly agree**

... demonstrates concern about public interest *

	1	2	3	4	5	
**Strongly disagree**	○	○	○	○	○	**Strongly agree**

...is sensationalized *

	1	2	3	4	5	
**Strongly disagree**	○	○	○	○	○	**Strongly agree**

...is immoral *

	1	2	3	4	5	
**Strongly disagree**	○	○	○	○	○	**Strongly agree**

Image/Post

Please have a detailed look at the following image/post and tick 'I am done' when you finish:
*

- ☐ I am done

Attention test

What was the rightmost recommendation in the last picture?

- ○ Wash your hands
- ○ Keep your distance
- ○ Wear a face covering

Feelings

Rate how positive or negative this post made you feel, ranging from **'sad'** to **'happy'**:

*

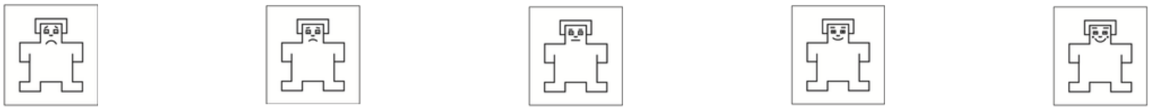

	1	2	3	4	5	6	7	8	9	
**Sad**	○	○	○	○	○	○	○	○	○	**Happy**

Rate how energetic this post made you feel, ranging from **'sleepy/apathetic'** to **'excited/energetic' :** *

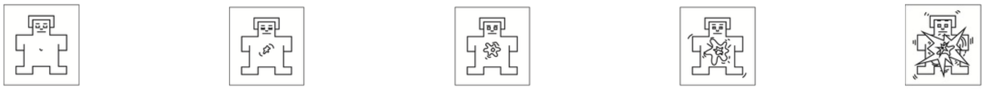

	1	2	3	4	5	6	7	8	9	
**Sleepy/Apathetic**	○	○	○	○	○	○	○	○	○	**Excited**

Rate how in control of the situation this post made you feel, ranging from **'no control'** to **'completely in control':** *

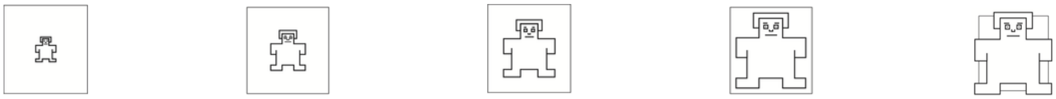

	1	2	3	4	5	6	7	8	9	
**No control**	○	○	○	○	○	○	○	○	○	**Completely in control**

Risk Assessment

After seeing this news post, how much anxiety do you feel in regards to the COVID-19 pandemic?*

	1	2	3	4	5	
**No anxiety at all**	○	○	○	○	○	**Very high anxiety**

After seeing the news post, how big of a risk do you think the COVID-19 pandemic poses to you or your family?*

	1	2	3	4	5	
**No risk at all**	○	○	○	○	○	**Very high risk**

Where 'dread' means to be in terror of, or fear intensely, how much do you dread the COVID-19 pandemic after seeing this news post?
*

	1	2	3	4	5	
**No dread at all**	○	○	○	○	○	**Very high dread**

After seeing the news post, what do you think is your likelihood of infection?*

	1	2	3	4	5	
**Very unlikely**	○	○	○	○	○	**Very likely**

After seeing the news post, how harmful do you think it would be if you got infected?*

	1	2	3	4	5	
**Not harmful at all**	○	○	○	○	○	**Very harmful**

Engagement

Are you likely to… (check where applicable)?*

- ☐ Share the post
- ☐ Read the post
- ☐ Comment on the post
- ☐ Ignore the post

Credibility

... can be trusted *

	1	2	3	4	5	
**Strongly disagree**	○	○	○	○	○	**Strongly agree**

...separates facts from opinion *

	1	2	3	4	5	
**Strongly disagree**	○	○	○	○	○	**Strongly agree**

...is factual *

	1	2	3	4	5	
**Strongly disagree**	○	○	○	○	○	**Strongly agree**

...tells the whole strory *

	1	2	3	4	5	
**Strongly disagree**	○	○	○	○	○	**Strongly agree**

...is accurate *

	1	2	3	4	5	
**Strongly disagree**	○	○	○	○	○	**Strongly agree**

...is unbiased *

	1	2	3	4	5	
**Strongly disagree**	○	○	○	○	○	**Strongly agree**

...is fair *

	1	2	3	4	5	
**Strongly disagree**	○	○	○	○	○	**Strongly agree**

... demonstrates concern about public interest *

	1	2	3	4	5	
**Strongly disagree**	○	○	○	○	○	**Strongly agree**

...is sensationalized *

	1	2	3	4	5	
**Strongly disagree**	○	○	○	○	○	**Strongly agree**

...is immoral *

	1	2	3	4	5	
**Strongly disagree**	○	○	○	○	○	**Strongly agree**

Image/Post

Please have a detailed look at the following image/post and tick 'I am done' when you finish:
*

☐ I am done

Attention test

Which months were represented by the bar chart in the last picture? *

- February and April
- October and December
- January and September

Feelings

Rate how positive or negative this post made you feel, ranging from **'sad'** to **'happy'**:

*

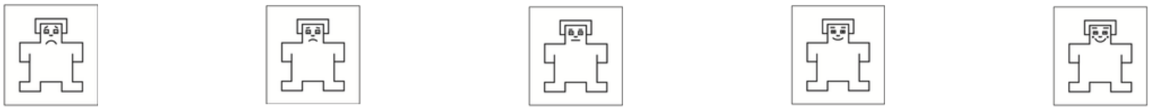

	1	2	3	4	5	6	7	8	9	
**Sad**	○	○	○	○	○	○	○	○	○	**Happy**

Rate how energetic this post made you feel, ranging from **'sleepy/apathetic'** to **'excited/energetic' :** *

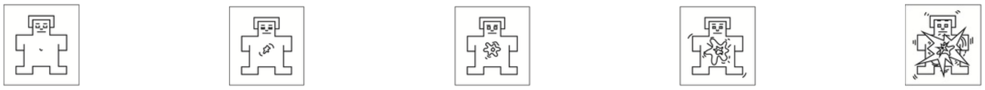

	1	2	3	4	5	6	7	8	9	
**Sleepy/Apathetic**	○	○	○	○	○	○	○	○	○	**Excited**

Rate how in control of the situation this post made you feel, ranging from **'no control'** to **'completely in control':** *

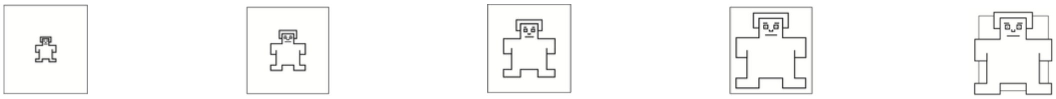

	1	2	3	4	5	6	7	8	9	
**No control**	○	○	○	○	○	○	○	○	○	**Completely in control**

Risk Assessment

After seeing this news post, how much anxiety do you feel in regards to the COVID-19 pandemic?*

	1	2	3	4	5	
**No anxiety at all**	○	○	○	○	○	**Very high anxiety**

After seeing the news post, how big of a risk do you think the COVID-19 pandemic poses to you or your family?*

	1	2	3	4	5	
**No risk at all**	○	○	○	○	○	**Very high risk**

Where ‘dread’ means to be in terror of, or fear intensely, how much do you dread the COVID-19 pandemic after seeing this news post?
*

	1	2	3	4	5	
**No dread at all**	○	○	○	○	○	**Very high dread**

After seeing the news post, what do you think is your likelihood of infection?*

	1	2	3	4	5	
**Very unlikely**	○	○	○	○	○	**Very likely**

After seeing the news post, how harmful do you think it would be if you got infected?*

	1	2	3	4	5	
**Not harmful at all**	○	○	○	○	○	**Very harmful**

Engagement

Are you likely to… (check where applicable)?*

- ☐ Share the post
- ☐ Read the post
- ☐ Comment on the post
- ☐ Ignore the post

Credibility

... can be trusted *

	1	2	3	4	5	
**Strongly disagree**	○	○	○	○	○	**Strongly agree**

...separates facts from opinion *

	1	2	3	4	5	
**Strongly disagree**	○	○	○	○	○	**Strongly agree**

...is factual *

	1	2	3	4	5	
**Strongly disagree**	○	○	○	○	○	**Strongly agree**

...tells the whole strory *

	1	2	3	4	5	
**Strongly disagree**	○	○	○	○	○	**Strongly agree**

...is accurate *

	1	2	3	4	5	
**Strongly disagree**	○	○	○	○	○	**Strongly agree**

...is unbiased *

	1	2	3	4	5	
**Strongly disagree**	○	○	○	○	○	**Strongly agree**

...is fair *

	1	2	3	4	5	
**Strongly disagree**	○	○	○	○	○	**Strongly agree**

... demonstrates concern about public interest *

	1	2	3	4	5	
**Strongly disagree**	○	○	○	○	○	**Strongly agree**

...is sensationalized *

	1	2	3	4	5	
**Strongly disagree**	○	○	○	○	○	**Strongly agree**

...is immoral *

	1	2	3	4	5	
**Strongly disagree**	○	○	○	○	○	**Strongly agree**

Image/Post

Please have a detailed look at the following image/post and tick 'I am done' when you finish:
*

- ☐ I am done

Attention test

What was the chart about in the last picture? *

- ○ Deaths with cancer and other deaths
- ○ Deaths with covid-19 and other deaths
- ○ Survivals from covid-19
- ○ Survivals from cancer

Feelings

Rate how positive or negative this post made you feel, ranging from **'sad'** to **'happy'**:

*

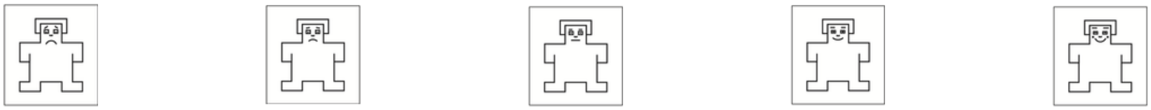

	1	2	3	4	5	6	7	8	9	
**Sad**	○	○	○	○	○	○	○	○	○	**Happy**

Rate how energetic this post made you feel, ranging from **'sleepy/apathetic'** to **'excited/energetic' :** *

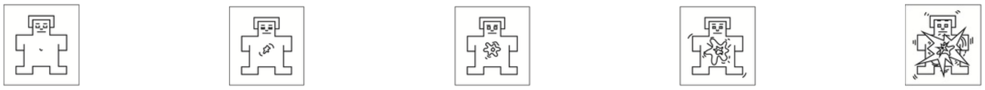

	1	2	3	4	5	6	7	8	9	
**Sleepy/Apathetic**	○	○	○	○	○	○	○	○	○	**Excited**

Rate how in control of the situation this post made you feel, ranging from **'no control'** to **'completely in control':** *

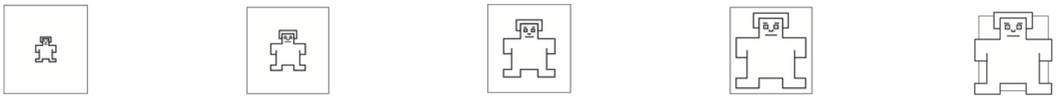

	1	2	3	4	5	6	7	8	9	
**No control**	○	○	○	○	○	○	○	○	○	**Completely in control**

Risk Assessment

After seeing this news post, how much anxiety do you feel in regards to the COVID-19 pandemic?*

	1	2	3	4	5	
**No anxiety at all**	○	○	○	○	○	**Very high anxiety**

After seeing the news post, how big of a risk do you think the COVID-19 pandemic poses to you or your family?*

	1	2	3	4	5	
**No risk at all**	○	○	○	○	○	**Very high risk**

Where ‘dread’ means to be in terror of, or fear intensely, how much do you dread the COVID-19 pandemic after seeing this news post?
*

	1	2	3	4	5	
**No dread at all**	○	○	○	○	○	**Very high dread**

After seeing the news post, what do you think is your likelihood of infection?*

	1	2	3	4	5	
**Very unlikely**	○	○	○	○	○	**Very likely**

After seeing the news post, how harmful do you think it would be if you got infected?*

	1	2	3	4	5	
**Not harmful at all**	○	○	○	○	○	**Very harmful**

Engagement

Are you likely to… (check where applicable)?*

- ☐ Share the post
- ☐ Read the post
- ☐ Comment on the post
- ☐ Ignore the post

Credibility

... can be trusted *

	1	2	3	4	5	
**Strongly disagree**	○	○	○	○	○	**Strongly agree**

...separates facts from opinion *

	1	2	3	4	5	
**Strongly disagree**	○	○	○	○	○	**Strongly agree**

...is factual *

	1	2	3	4	5	
**Strongly disagree**	○	○	○	○	○	**Strongly agree**

...tells the whole strory *

	1	2	3	4	5	
**Strongly disagree**	○	○	○	○	○	**Strongly agree**

...is accurate *

	1	2	3	4	5	
**Strongly disagree**	○	○	○	○	○	**Strongly agree**

...is unbiased *

	1	2	3	4	5	
**Strongly disagree**	○	○	○	○	○	**Strongly agree**

...is fair *

	1	2	3	4	5	
**Strongly disagree**	○	○	○	○	○	**Strongly agree**

... demonstrates concern about public interest *

	1	2	3	4	5	
**Strongly disagree**	○	○	○	○	○	**Strongly agree**

...is sensationalized *

	1	2	3	4	5	
**Strongly disagree**	○	○	○	○	○	**Strongly agree**

...is immoral *

	1	2	3	4	5	
**Strongly disagree**	○	○	○	○	○	**Strongly agree**

Image/Post

Please have a detailed look at the following image/post and tick 'I am done' when you finish:
*

- ☐ I am done

Attention test

What was represented on the left and right sides of the chart in the last picture? *

- ○ Boys and Girls
- ○ Males and Females
- ○ Educated and Uneducated

Feelings

Rate how positive or negative this post made you feel, ranging from **'sad'** to **'happy'**:

*

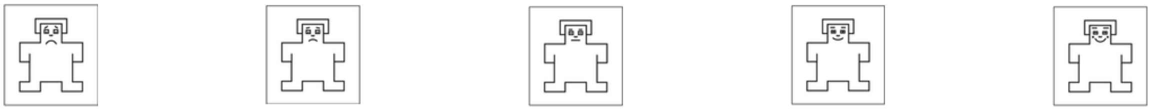

	1	2	3	4	5	6	7	8	9	
**Sad**	○	○	○	○	○	○	○	○	○	**Happy**

Rate how energetic this post made you feel, ranging from **'sleepy/apathetic'** to **'excited/energetic' :** *

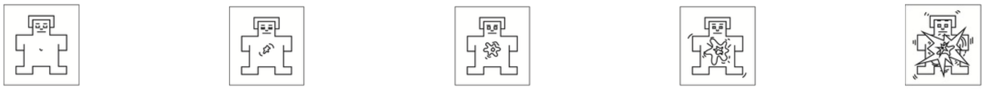

	1	2	3	4	5	6	7	8	9	
**Sleepy/Apathetic**	○	○	○	○	○	○	○	○	○	**Excited**

Rate how in control of the situation this post made you feel, ranging from **'no control'** to **'completely in control':** *

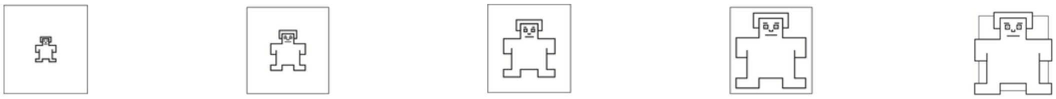

	1	2	3	4	5	6	7	8	9	
**No control**	○	○	○	○	○	○	○	○	○	**Completely in control**

Risk Assessment

After seeing this news post, how much anxiety do you feel in regards to the COVID-19 pandemic?*

	1	2	3	4	5	
**No anxiety at all**	○	○	○	○	○	**Very high anxiety**

After seeing the news post, how big of a risk do you think the COVID-19 pandemic poses to you or your family?*

	1	2	3	4	5	
**No risk at all**	○	○	○	○	○	**Very high risk**

Where 'dread' means to be in terror of, or fear intensely, how much do you dread the COVID-19 pandemic after seeing this news post?
*

	1	2	3	4	5	
**No dread at all**	○	○	○	○	○	**Very high dread**

After seeing the news post, what do you think is your likelihood of infection?*

	1	2	3	4	5	
**Very unlikely**	○	○	○	○	○	**Very likely**

After seeing the news post, how harmful do you think it would be if you got infected?*

	1	2	3	4	5	
**Not harmful at all**	○	○	○	○	○	**Very harmful**

Engagement

Are you likely to… (check where applicable)?*

- ☐ Share the post
- ☐ Read the post
- ☐ Comment on the post
- ☐ Ignore the post

Credibility

... can be trusted *

	1	2	3	4	5	
**Strongly disagree**	○	○	○	○	○	**Strongly agree**

...separates facts from opinion *

	1	2	3	4	5	
**Strongly disagree**	○	○	○	○	○	**Strongly agree**

...is factual *

	1	2	3	4	5	
**Strongly disagree**	○	○	○	○	○	**Strongly agree**

...tells the whole strory *

	1	2	3	4	5	
**Strongly disagree**	◯	◯	◯	◯	◯	**Strongly agree**

...is accurate *

	1	2	3	4	5	
**Strongly disagree**	◯	◯	◯	◯	◯	**Strongly agree**

...is unbiased *

	1	2	3	4	5	
**Strongly disagree**	◯	◯	◯	◯	◯	**Strongly agree**

...is fair *

	1	2	3	4	5	
**Strongly disagree**	◯	◯	◯	◯	◯	**Strongly agree**

... demonstrates concern about public interest *

	1	2	3	4	5	
**Strongly disagree**	◯	◯	◯	◯	◯	**Strongly agree**

...is sensationalized *

	1	2	3	4	5	
**Strongly disagree**	◯	◯	◯	◯	◯	**Strongly agree**

...is immoral *

	1	2	3	4	5	
**Strongly disagree**	◯	◯	◯	◯	◯	**Strongly agree**

Demographic Information and Internet Usage Habits

Your Age: *

[ ]

Your Gender: *

- ○ Male
- ○ Female

What is the highest degree or education level you have completed:*

- ○ High School
- ○ Bachelor's degree
- ○ Master's degree
- ○ Ph.D or higher
- ○ Prefer not to say
- ○ None

Which of the following social media do you use? *

- ☐ Facebook
- ☐ Instagram
- ☐ Twitter
- ☐ YouTube
- ☐ LinkedIn
- ☐ Other - Write In (Required)

[ ] *

How many hours per day do you spend on social media? *

[ ]

What is your main source of news about the world? *

- Television
- News websites
- Social media
- Newspaper
- Podcasts
- Radio
- Other - Write In (Required)

How active are you on social media through commenting, sharing, liking or posting content? *

	1	2	3	4	5	
**Not active at all**						**Extremely active**

Thank You!

Thank you for taking our survey. Your response is very important to us.

Here is your completion code: [question('value'), id='301']
